# Supplementary material for: Exome Sequencing Identifies Genes and Gene Sets Contributing to Severe Childhood Obesity, Linking PHIP Variants to Repressed POMC Transcription
Source: Cell Metab. 2020 Jun 2;31(6):1107–1119.e12. doi: 10.1016/j.cmet.2020.05.007 (PMC7267775; doi:10.1016/j.cmet.2020.05.007)
Supplement: Document S1. Figures S1–S7 and Tables S3, S4, and S7 [file mmc1.pdf]

Cell Metabolism, Volume *31*

Supplemental Information

Exome Sequencing Identifies Genes and Gene Sets Contributing to Severe Childhood Obesity, Linking *PHIP* Variants to Repressed *POMC* Transcription

Gaëlle Marenne, Audrey E. Hendricks, Aliki Perdikari, Rebecca Bounds, Felicity Payne, Julia M. Keogh, Christopher J. Lelliott, Elana Henning, Saad Pathan, Sofie Ashford, Elena G. Bochukova, Vanisha Mistry, Allan Daly, Caroline Hayward, INTERVAL, UK10K Consortium, Nicholas J. Wareham, Stephen O'Rahilly, Claudia Langenberg, Eleanor Wheeler, Eleftheria Zeggini, I. Sadaf Farooqi, and Inês Barroso

Supplementary Figures and Tables

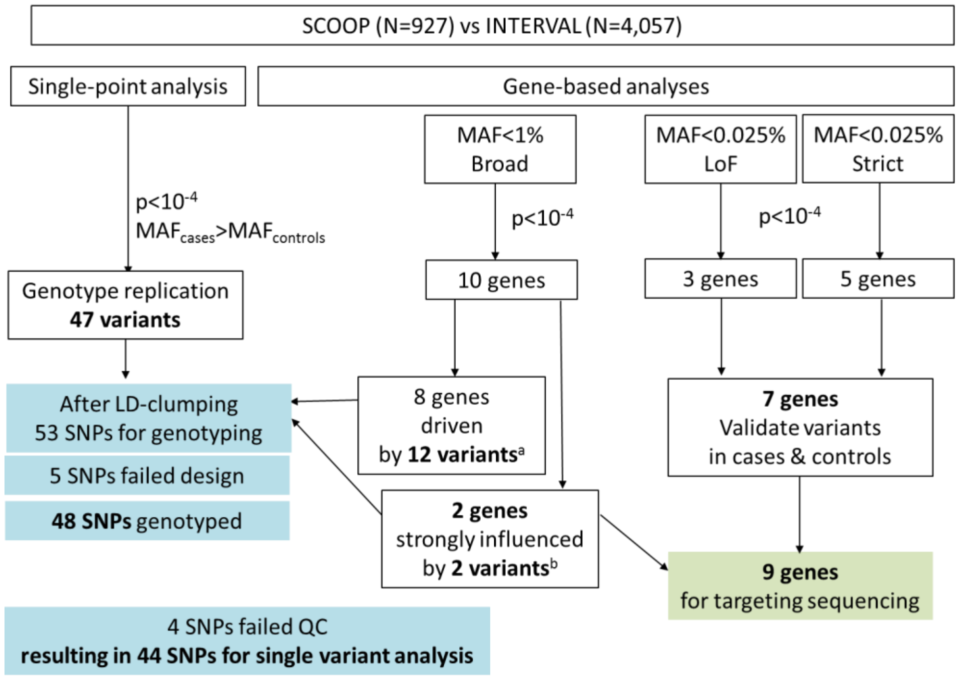

Supplementary Figure 1, Related to Figure 1 – Flow Diagram Single-point and Gene-based Analyses. Flow diagram showing the different analysis and main association results based on stage 1 analysis with 927 SCOOP cases and 4,057 INTERVAL participants as controls.

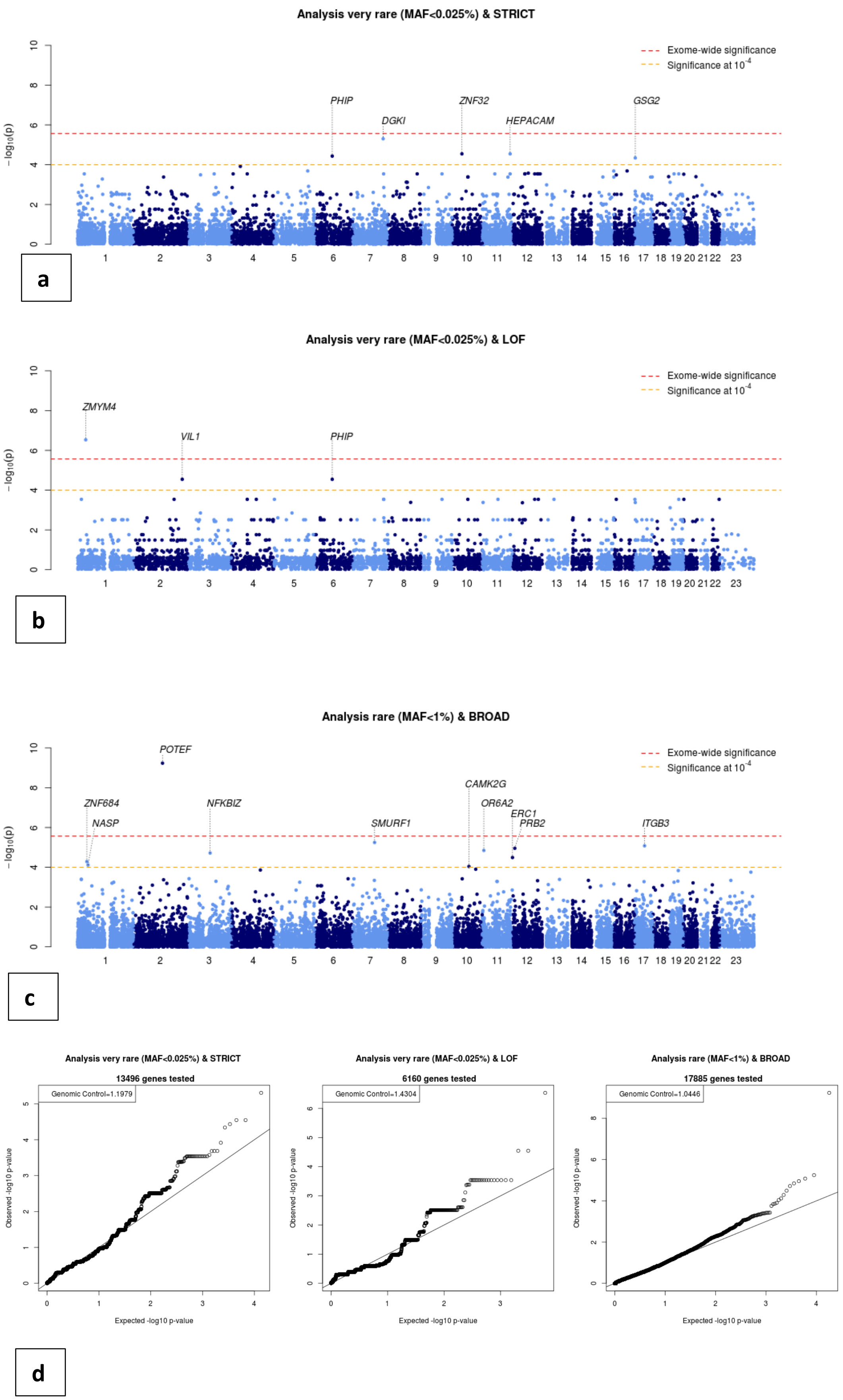
Analysis very rare (MAF<0.025%) & STRICT
Exome-wide significance
Significance at 10^{-4}
$-\log_{10}(p)$
PHIP
DGKI
ZNF32
HEPACAM
GSG2
1 2 3 4 5 6 7 8 9 10 11 12 13 14 15 16 17 18 19 20 21 22 23
a
Analysis very rare (MAF<0.025%) & LOF
Exome-wide significance
Significance at 10^{-4}
$-\log_{10}(p)$
ZMYM4
VIL1
PHIP
1 2 3 4 5 6 7 8 9 10 11 12 13 14 15 16 17 18 19 20 21 22 23
b
Analysis rare (MAF<1%) & BROAD
Exome-wide significance
Significance at 10^{-4}
$-\log_{10}(p)$
POTEF
ZNF684
NASP
NFKBIZ
SMURF1
CAMK2G
OR6A2
ERC1
PRB2
ITGB3
1 2 3 4 5 6 7 8 9 10 11 12 13 14 15 16 17 18 19 20 21 22 23
c
Analysis very rare (MAF<0.025%) & STRICT
13496 genes tested
Genomic Control=1.1979
Analysis very rare (MAF<0.025%) & LOF
6160 genes tested
Genomic Control=1.4304
Analysis rare (MAF<1%) & BROAD
17885 genes tested
Genomic Control=1.0446
Observed -log10 p-value
Expected -log10 p-value
d

Supplementary Figure 2, Related to STAR Methods – Manhattan and QQ plots of gene-based association results. Manhattan plot STRICT analysis (a), Manhattan plot LOF analysis (b) Manhattan plot BROAD analysis (c). Genes taken forward for validation in stage 2 analysis are annotated. QQ plots of association results for gene-based analysis (d): left (STRICT analysis), middle (LOF analysis), right (BROAD analysis).

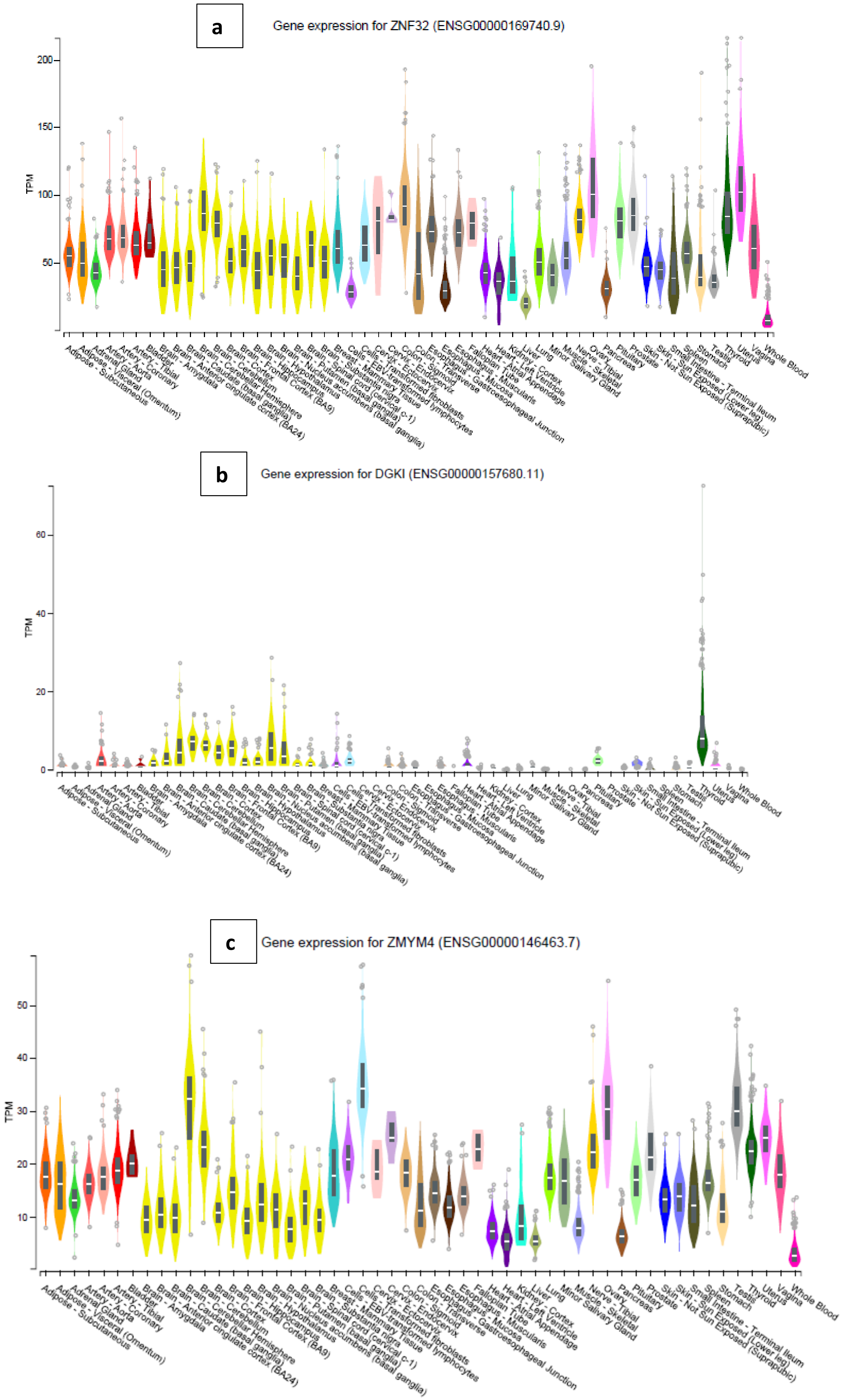
a
Gene expression for ZNF32 (ENSG00000169740.9)
TPM
200
150
100
50
b
Gene expression for DGKI (ENSG00000157680.11)
TPM
60
40
20
0
c
Gene expression for ZMYM4 (ENSG00000146463.7)
TPM
50
40
30
20
10
Adipose - Subcutaneous
Adipose - Visceral (Omentum)
Adrenal Gland
Artery - Aorta
Artery - Coronary
Artery - Tibial
Bladder
Brain - Amygdala
Brain - Anterior cingulate cortex (BA24)
Brain - Caudate (basal ganglia)
Brain - Cerebellar Hemisphere
Brain - Cerebellum
Brain - Cortex
Brain - Frontal Cortex (BA9)
Brain - Hippocampus
Brain - Hypothalamus
Brain - Nucleus accumbens (basal ganglia)
Brain - Putamen (basal ganglia)
Brain - Spinal cord (cervical c-1)
Brain - Substantia nigra
Breast - Mammary Tissue
Cells - EBV-transformed lymphocytes
Cells - Transformed fibroblasts
Cervix - Ectocervix
Cervix - Endocervix
Colon - Sigmoid
Colon - Transverse
Esophagus - Gastroesophageal Junction
Esophagus - Mucosa
Esophagus - Muscularis
Fallopian Tube
Heart - Atrial Appendage
Heart - Left Ventricle
Kidney - Cortex
Liver
Lung
Minor Salivary Gland
Muscle - Skeletal
Nerve - Tibial
Ovary
Pancreas
Pituitary
Prostate
Skin - Not Sun Exposed (Suprapubic)
Skin - Sun Exposed (Lower leg)
Small Intestine - Terminal Ileum
Spleen
Stomach
Testis
Thyroid
Uterus
Vagina
Whole Blood

Supplementary Figure 3, Related to STAR Methods – GTEX expression profiles of select genes.
ZNF32 (a), *DGKI* (b), *ZMYM4* (c).

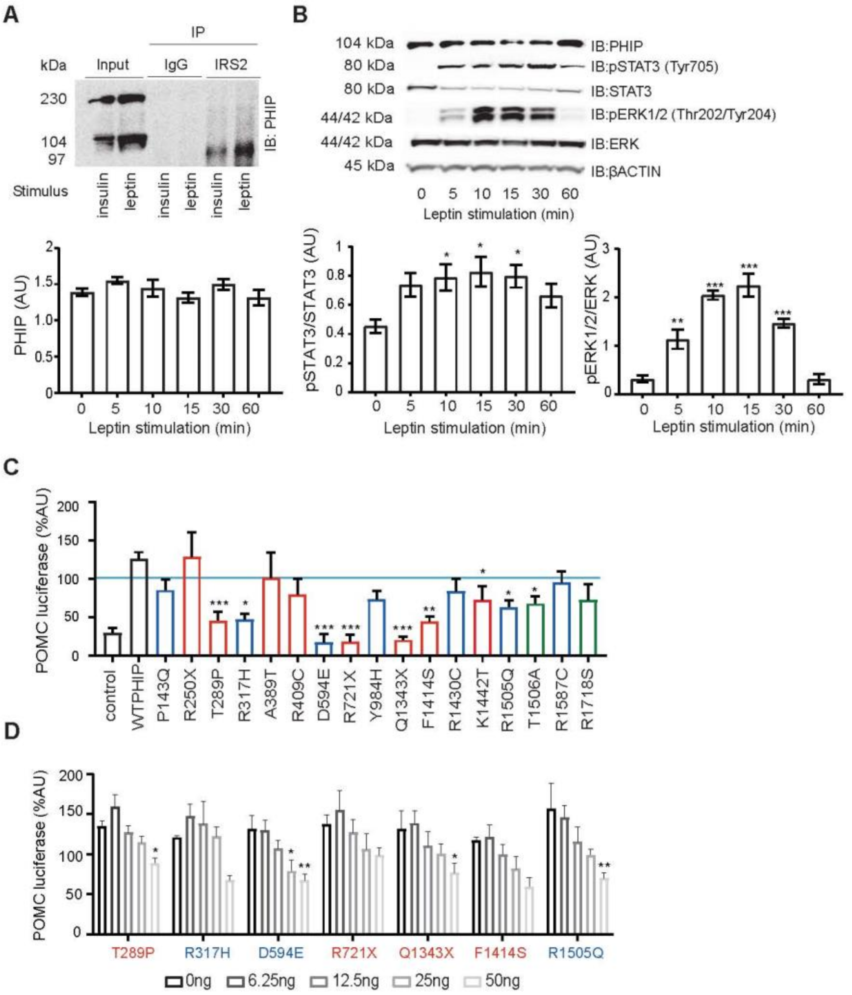

Supplementary Figure 4, Related to Figure 3 - Functional characterisation of WT and mutant forms of PHIP. A) Representative western blot depicting the interaction between endogenous PHIP and IRS2 upon insulin and leptin stimulation. The 230kDa and 104kDa endogenous PHIP isoforms were used as input as shown. B) Representative western blot of endogenous PHIP, pSTAT3 and pERK upon leptin stimulation and quantification of PHIP protein expression and STAT3 and ERK phosphorylation in different time points. n= 4, mean+/-SEM, ordinary one-way ANOVA multiple comparisons to time point 0min, *p<0.05, **p<0.01, ***p<0.001. C) Effect of human PHIP mutants on POMC transcription compared to WT in leptin stimulated conditions, n=4-6, mean + SEM, ordinary one-way ANOVA multiple comparisons to WT, *p<0.05, **p<0.01, ***p<0.001. Blue line : WT in basal conditions D) Dominant negative effect of human PHIP mutants in POMC transcription in leptin stimulated conditions, n=3, mean + SEM, ordinary one-way ANOVA multiple comparisons to 0ng *p<0.05, **p<0.01, ***p<0.001.

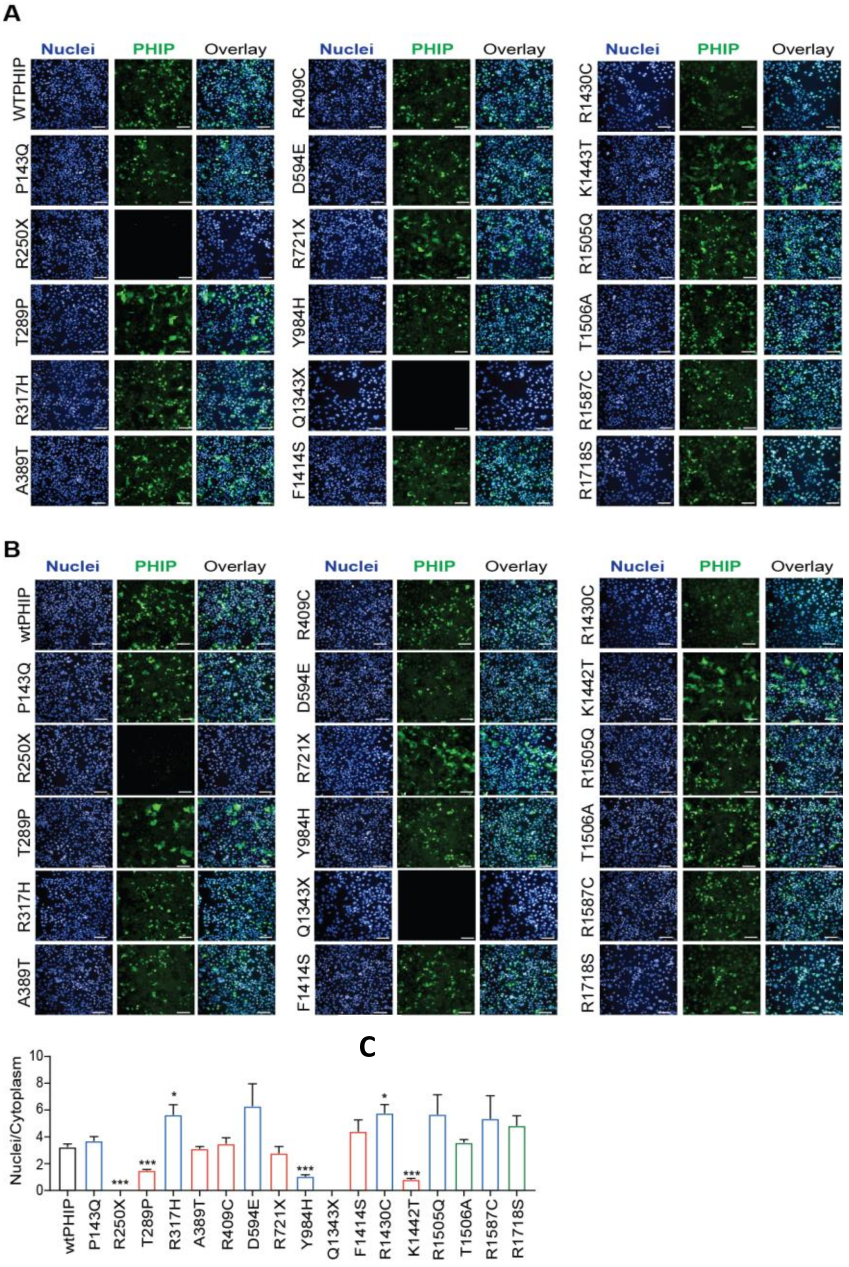
A
Nuclei
PHIP
Overlay
WTPHIP
P143Q
R250X
T289P
R317H
A389T
R409C
D594E
R721X
Y984H
Q1343X
F1414S
R1430C
K1443T
R1505Q
T1506A
R1587C
R1718S
B
Nuclei
PHIP
Overlay
wtPHIP
P143Q
R250X
T289P
R317H
A389T
R409C
D594E
R721X
Y984H
Q1343X
F1414S
R1430C
K1442T
R1505Q
T1506A
R1587C
R1718S
C
Nuclei/Cytoplasm
0
2
4
6
8
10
*
***
*
***
***
***
wtPHIP
P143Q
R250X
T289P
R317H
A389T
R409C
D594E
R721X
Y984H
Q1343X
F1414S
R1430C
K1442T
R1505Q
T1506A
R1587C
R1718S

Supplementary Figure 5, Related to Figure 3 - Cellular localisation of WT and mutant forms of PHIP. A-B) Representative confocal fluorescence microscopy images showing protein localization of PHIP mutants in basal (A) and leptin stimulated (B) conditions and (C) quantification of nuclei to cytoplasm ratio of PHIP subcellular localization for leptin stimulated conditions, n=3, mean + SEM, two-tailed unpaired T-test with Welch's correction, *p<0.05, **p<0.01, ***p<0.001.

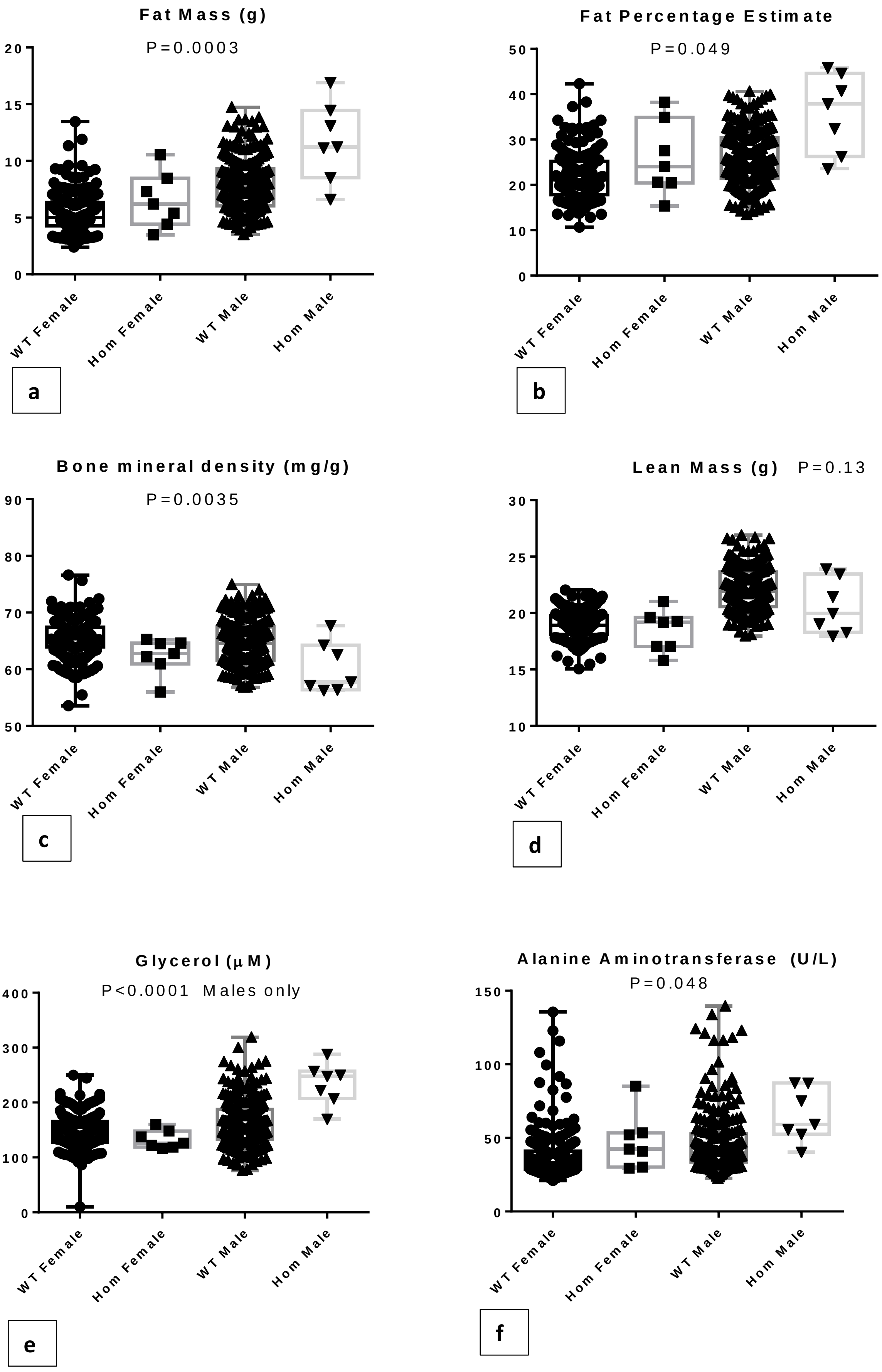
Fat Mass (g)
P=0.0003
a
Fat Percentage Estimate
P=0.049
b
Bone mineral density (mg/g)
P=0.0035
c
Lean Mass (g) P=0.13
d
Glycerol (µM)
P<0.0001 Males only
e
Alanine Aminotransferase (U/L)
P=0.048
f
WT Female
Hom Female
WT Male
Hom Male

Supplementary Figure 6, Related to STAR methods– Phenotyping results in wild-type and *Dgki*$^{em1(IMPC)Wtsi}$ homozygous mice. Boxplots showing fat mass (a), fat percentage (b), bone mineral density (c), lean mass (d), glycerol (e) and alanine aminotransferase (f). Wild-type (WT) and *Dgki*$^{em1(IMPC)Wtsi}$ homozygous mice (Hom) results are shown. P-values for genotype effect are shown. Numbers included in (a-d) are Males: WT n=229, Hom n=7; Females: WT n=225, Hom n=7, (e) M: WT n=228, Hom n=7; Females: WT n=223, Hom n=7; (f) Males: WT n=227, Hom n=7; Females: WT n=222, Hom n=7.

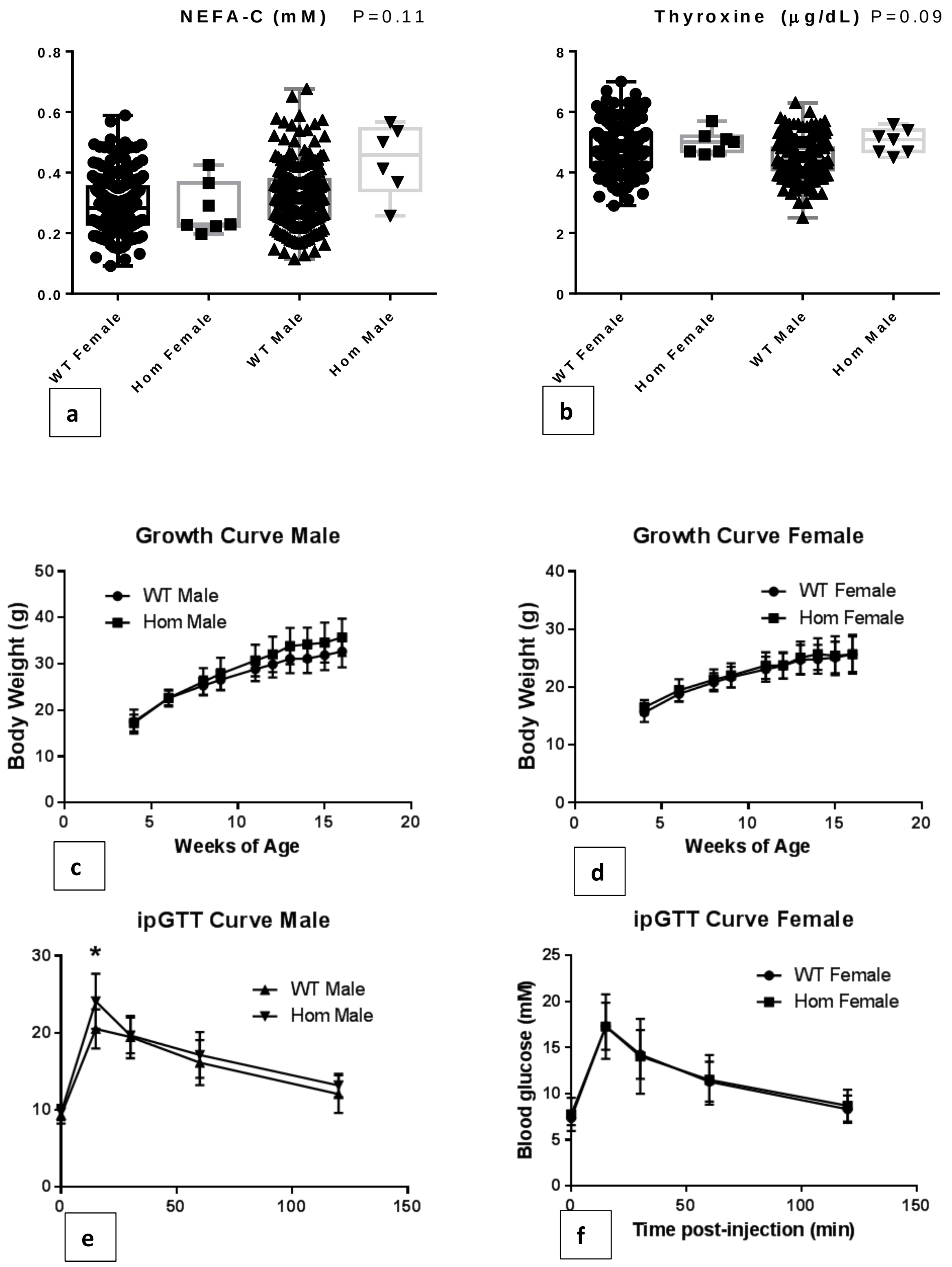
NEFA-C (mM) P=0.11
0.0
0.2
0.4
0.6
0.8
WT Female
Hom Female
WT Male
Hom Male
a
Thyroxine (µg/dL) P=0.09
0
2
4
6
8
WT Female
Hom Female
WT Male
Hom Male
b
Growth Curve Male
Body Weight (g)
WT Male
Hom Male
Weeks of Age
c
Growth Curve Female
Body Weight (g)
WT Female
Hom Female
Weeks of Age
d
ipGTT Curve Male
*
WT Male
Hom Male
e
ipGTT Curve Female
Blood glucose (mM)
WT Female
Hom Female
Time post-injection (min)
f

Supplementary Figure 7, Related to STAR methods– Phenotyping results in wild-type and *Dgki*$^{em1(IMPC)Wtsi}$ homozygous mice. Boxplots showing NEFA-X (a) and thyroxine (b). Growth curves for male (c) and female (d) mice are shown with mean and standard deviation. Results from ipGTT for male (e) and female (f) mice are shown. Wild-type (WT) and *Dgki*$^{em1(IMPC)Wtsi}$ homozygous mice (Hom) results are shown. P-values for genotype effect are shown for (a-b). All genotype effects (combined male and female are non-significant for growth (c-d): week 4 (p=0.530), week 9 (p=0.400), week 13 (p=0.076), week 16 (p=0.101). P-values for genotype effects (combined male and female) for blood glucose concentration (e-f): T0 (p=0.354), T15 (p=0.040), T30 (p=0.913), T60 (p=0.462), T120 (p=0.366). Numbers included are (a) Males: WT n=221, Hom n=7; Females: WT n=220, Hom n=7; (b) Males: WT n=227, Hom n=7; Females: WT n=7, Hom n=222; (c) WT n=230, Hom n=7; (d) WT n=226, Hom n=7; (e) WT n=189, Hom n=6; and (f) WT n=180, Hom n=5.

gene	SCOOP strict	SCOOP LoF	SCOOP syn	gnomAD strict	gnomAD LoF	gnomAD syn	strict / syn scoop	strict / syn gnomad	strict / syn p-value	lof / syn scoop	lof / syn gnomad	lof / syn p-value
DGKI	7	1	6	46	7	102	1.167	0.451	1.05E-01	0.167	0.069	4.78E-01
ERC1	2	0	6	88	15	83	0.333	1.06	1.35E-01	0	0.181	1.65E-01
GSG2	5	4	3	37	21	73	1.667	0.507	1.09E-01	1.333	0.288	5.72E-02
HEPACAM	5	0	1	28	1	39	5	0.718	4.41E-02	NA	0.026	NA
NASP	1	0	9	19	4	76	0.111	0.25	4.12E-01	NA	0.053	NA
PHIP	9	4	5	47	5	129	1.8	0.364	4.96E-03	0.8	0.039	4.93E-04
VIL1	6	4	4	89	17	161	1.5	0.553	1.25E-01	1	0.106	4.99E-03
ZMYM4	7	6	4	58	10	123	1.75	0.472	3.77E-02	1.5	0.081	7.10E-05
ZNF32	4	1	2	8	1	33	2	0.242	2.14E-02	0.5	0.03	9.92E-02

ST3, Related to STAR Methods - ProxECAT results for the 9 genes. Column headers are: gene -gene symbol; strict -number of alternate alleles in STRICT category; LoF - number of alternate alleles in LoF category; syn - number of synonymous alternate alleles; strict / syn - ratio of strict to synonymous alternate alleles; lof / syn - ratio of strict to synonymous alternate alleles; p-value - ProxECAT p-value testing for association with the gene region.

Gene	Analysis	p-value	# variants	Allele counts Cases	Allele counts 1958BC	OR [95% CI] (adjusted)
DGKI	very rare (MAF<0.025%) & STRICT	0.8230	2	1	3	0.76 [0.08-7.32]
DGKI	very rare (MAF<0.025%) & LOF	0.1307	1	1	0	Inf (6.85 [0.28-168.44])
PHIP	very rare (MAF<0.025%) & STRICT	0.9138	3	1	2	1.14 [0.1-12.6]
PHIP	very rare (MAF<0.025%) & LOF	-	0	0	0	-
ZMYM4	very rare (MAF<0.025%) & STRICT	0.1725	3	2	1	4.57 [0.41-50.41]
ZMYM4	very rare (MAF<0.025%) & LOF	-	0	0	0	-

ST4, related to STAR Methods - Results from gene-based analyses in UK10K-Adult Obese versus 1958BC controls for 3 selected genes (*PHIP, DGKI* and *ZMYM4*). There were no LOF or STRICT variants with MAF<0.025% for analysis in *ZNF32*. Column headers are: Gene - Gene symbol; Analysis - Analysis performed; p-value - burden test p-value; # variants - Number of variants contributing to the gene-based test; Allele counts Cases - Allele counts in adult obese cases; Allele counts 1958BC - Allele counts in 1958BC controls; OR [95% CI] (adjusted) - Odds ratio [95% confidence interval]. If allele count was 0 in cases or controls, an adjusted OR and its CI was computed by adding 0.5 to each of the 2 by 2 table cell.

Stage 1			
**Analysis**	**BROAD**	**STRICT**	**LOF**
AC INTERVAL (N=4057)	29	6	0
AC SCOOP (N=927)	14	9	4
AC SCOOP with DD (N=226)	7	4	2
AC SCOOP without DD (N=701)	7	5	2
p-value SCOOP v controls	0.0059	3.67E-05	2.84E-05
p-value SCOOP with DD v controls	1.48E-10	8.67E-07	2.06E-09
p-value SCOOP without DD v controls	0.0880	0.0040	0.0007
OR SCOOP v controls	2.11 [1.12-4]	6.57 [2.34-18.45]	Inf(39.41 [2.12-732.02])
OR SCOOP with DD v controls	4.33 [1.9-9.9]	11.98 [3.38-42.45]	Inf(89.83 [4.31-1871.85])
OR SCOOP without DD v controls	1.4 [0.61-3.19]	4.82 [1.47-15.81]	Inf(28.95 [1.39-602.99])
**Stage 2 (post sequence validation)**			
**Analysis**	**BROAD**	**STRICT**	**LOF**
AC FENLAND (N=2647)	9	3	0
AC SCOOP (N=1810)	20	5	3
AC SCOOP with DD (N=411)	4	2	2
AC SCOOP without DD (N=1399)	16	3	1
p-value SCOOP v controls	0.0032	0.2070	0.0361
p-value SCOOP with DD v controls	0.0210	0.0814	0.0003
p-value SCOOP without DD v controls	0.0021	0.4267	0.1689
OR SCOOP v controls	3.28 [1.49-7.2]	2.45 [0.59-10.27]	Inf(10.26 [0.53-198.57])
OR SCOOP with DD v controls	2.9 [0.89-9.42]	4.34 [0.72-25.95]	Inf(32.28 [1.55-672.63])
OR SCOOP without DD v controls	3.39 [1.5-7.68]	1.9 [0.38-9.43]	Inf(5.69 [0.23-139.57])
**META-ANALYSIS (post sequence validation)**			
**Analysis**	**BROAD**	**STRICT**	**LOF**
AC controls (N=6704)	38	9	0
AC SCOOP (N=2737)	34	14	7
AC SCOOP with DD (N=637)	11	6	4
AC SCOOP without DD (N=2100)	23	8	3
p-value SCOOP v controls	0.0002	9.81E-05	1.23E-05
p-value SCOOP with DD v controls	3.75E-07	3.75E-06	3.19E-10
p-value SCOOP without DD v controls	0.0030	0.0091	0.0006
OR SCOOP v controls	2.49 [1.57-3.96]	4.35 [1.88-10.06]	Inf(39.74 [2.27-695.83])
OR SCOOP with DD v controls	3.81 [1.95-7.46]	8.54 [3.04-23.99]	Inf(95.01 [5.11-1765.21])
OR SCOOP without DD v controls	2.19 [1.3-3.68]	3.18 [1.23-8.25]	Inf(26.95 [1.39-521.79])

ST7, Related to Figure 2 - Results from *PHIP* gene-based analyses stratifying cases according the presence or absence of developmental delay. Column headers: AC -allele count, i.e. number of the rare alleles; N - Total number of alleles; DD - developmental delay,refers to analyses limitesd to obesity cases who also have developmental delay, or to those that are obese without developmental delay (without DD); OR - odds ratio and [95% CI]. If allele count was 0 in cases or controls, an adjusted OR and its CI was computed by adding 0.5 to each of the 2 by 2 table cell; Stage1, Stage 2- refer to analyses performed in Stage 1 and Stage 2 participants with either BROAD, STRICT or LOF tests.
