# Supplementary material for: Exome Sequencing Identifies Genes and Gene Sets Contributing to Severe Childhood Obesity, Linking PHIP Variants to Repressed POMC Transcription
Source: Cell Metab. 2020 Jun 2;31(6):1107–1119.e12. doi: 10.1016/j.cmet.2020.05.007 (PMC7267775; doi:10.1016/j.cmet.2020.05.007)
Supplement: Document S2. Article plus Supplemental Information [file mmc8.pdf]

Cell Metabolism

Article

Exome Sequencing Identifies Genes and Gene Sets Contributing to Severe Childhood Obesity, Linking *PHIP* Variants to Repressed *POMC* Transcription

Graphical Abstract

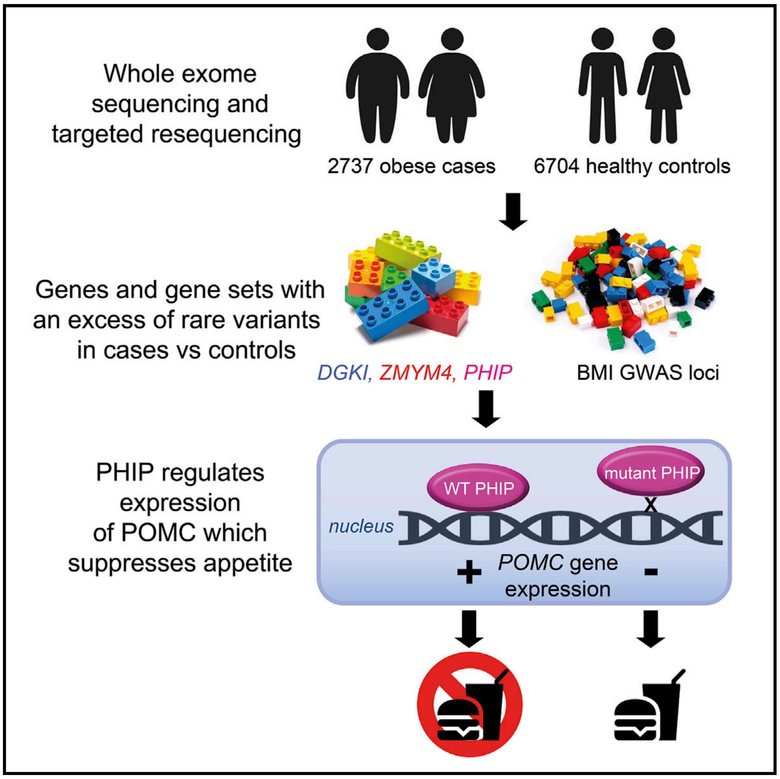

Authors

Gaëlle Marenne, Audrey E. Hendricks, Aliki Perdikari, ..., Eleftheria Zeggini, I. Sadaf Farooqi, Inês Barroso

Correspondence

isf20@cam.ac.uk (I.S.F.),
ines.barroso@exeter.ac.uk (I.B.)

In Brief

Childhood obesity can be caused by penetrant mutations in a number of genes controlling appetite and body weight. Marenne et al. identify three genes with mutations with variable penetrance in a continuum of causality in childhood obesity, and demonstrate that variants in *PHIP* repress *POMC* transcription.

Highlights

- Three genes (*PHIP*, *DGKI*, and *ZMYM4*) are linked to severe childhood obesity
- Wild-type *PHIP* enhances *POMC* transcription, but variants in *PHIP* repress *POMC*
- Rare variants in BMI-associated loci from GWAS are enriched in severe obesity
- Genetic architecture of severe childhood obesity reveals a continuum of causality

Marenne et al., 2020, Cell Metabolism *31*, 1107–1119
June 2, 2020 © 2020 The Author(s). Published by Elsevier Inc.
https://doi.org/10.1016/j.cmet.2020.05.007

Cell Metabolism

CellPress
OPEN ACCESS

Article

Exome Sequencing Identifies Genes and Gene Sets Contributing to Severe Childhood Obesity, Linking *PHIP* Variants to Repressed *POMC* Transcription

Gaëlle Marenne,[1,2] Audrey E. Hendricks,[1,3] Aliki Perdikari,[4] Rebecca Bounds,[4] Felicity Payne,[1] Julia M. Keogh,[4] Christopher J. Lelliott,[1] Elana Henning,[4] Saad Pathan,[4] Sofie Ashford,[4] Elena G. Bochukova,[4] Vanisha Mistry,[4] Allan Daly,[1] Caroline Hayward,[5,6] INTERVAL, UK10K Consortium, Nicholas J. Wareham,[7] Stephen O'Rahilly,[8] Claudia Langenberg,[7] Eleanor Wheeler,[1,7] Eleftheria Zeggini,[1,9] I. Sadaf Farooqi,[4,12,*] and Inês Barroso[1,7,10,11,12,13,*]

[1]Wellcome Sanger Institute, Cambridge, UK
[2]Inserm, Univ Brest, EFS, UMR 1078, GGB, 29200 Brest, France
[3]Mathematical and Statistical Sciences, University of Colorado Denver, Denver, CO, USA
[4]University of Cambridge Metabolic Research Laboratories and NIHR Cambridge Biomedical Research Centre, Wellcome-MRC Institute of Metabolic Science, Addenbrooke's Hospital, Cambridge, UK
[5]MRC Human Genetics Unit, Institute of Genetics and Molecular Medicine, University of Edinburgh, Edinburgh, UK
[6]Generation Scotland, Centre for Genomic and Experimental Medicine, Institute of Genetics and Molecular Medicine, University of Edinburgh, Edinburgh, UK
[7]University of Cambridge MRC Epidemiology Unit, Wellcome Trust-MRC Institute of Metabolic Science, Addenbrooke's Hospital, Cambridge, UK
[8]MRC Metabolic Diseases Unit, Wellcome-MRC Institute of Metabolic Science, Addenbrooke's Hospital, Cambridge, UK
[9]Institute of Translational Genomics, Helmholtz Zentrum München – German Research Center for Environmental Health, Neuherberg, Germany
[10]Present address: Exeter Centre of ExcEllence in Diabetes (ExCEED), University of Exeter Medical School, RILD building, Exeter, UK
[11]Twitter: @InesBarroso4
[12]These authors contributed equally
[13]Lead Contact
*Correspondence: isf20@cam.ac.uk (I.S.F.), ines.barroso@exeter.ac.uk (I.B.)
https://doi.org/10.1016/j.cmet.2020.05.007

SUMMARY

Obesity is genetically heterogeneous with monogenic and complex polygenic forms. Using exome and targeted sequencing in 2,737 severely obese cases and 6,704 controls, we identified three genes (*PHIP*, *DGKI*, and *ZMYM4*) with an excess burden of very rare predicted deleterious variants in cases. In cells, we found that nuclear PHIP (pleckstrin homology domain interacting protein) directly enhances transcription of pro-opiomelanocortin (POMC), a neuropeptide that suppresses appetite. Obesity-associated *PHIP* variants repressed POMC transcription. Our demonstration that PHIP is involved in human energy homeostasis through transcriptional regulation of central melanocortin signaling has potential diagnostic and therapeutic implications for patients with obesity and developmental delay. Additionally, we found an excess burden of predicted deleterious variants involving genes nearest to loci from obesity genome-wide association studies. Genes and gene sets influencing obesity with variable penetrance provide compelling evidence for a continuum of causality in the genetic architecture of obesity, and explain some of its missing heritability.

Context and Significance

Obese children are often stigmatized and experience health problems such as diabetes and heart disease in later life. Finding the cause of their obesity may lead to new treatments. In some cases, faulty genes underly severe childhood obesity. In this study, researchers in the United Kingdom and their colleagues aimed to discover new genes linked to severe childhood obesity and found three candidates. One gene, *PHIP*, affected childhood obesity with learning difficulties. They demonstrate that *PHIP* works by controlling another gene, *POMC*, which is known to regulate appetite. This finding means that children with faults in the gene *PHIP* may benefit from existing treatments. Further studies will be required to fully evaluate these genes in a broader context.

This is an open access article under the CC BY license (http://creativecommons.org/licenses/by/4.0/).

INTRODUCTION

The rising prevalence of obesity is largely driven by the consumption of high-calorie foods and reduced levels of physical activity at work and in leisure time, which contribute to sustained positive energy balance and weight gain. However, family, twin, and adoption studies have consistently demonstrated that 40%–70% of the variation in body weight in a given environment is attributable to genetic variation within the population (Allison et al., 1996). As such, finding even a single gene that contributes to the regulation of body weight is important as it provides insights into the mechanisms underlying the development of obesity and may identify potential targets for future weight loss therapy.

To date, several different approaches have been used to identify genes involved in human energy homeostasis. Candidate gene studies led to the identification of very rare variants that cause monogenic forms of severe obesity mostly by impacting the function of proteins involved in the central leptin-melanocortin pathway (Doche et al., 2012; O'Rahilly and Farooqi, 2008; Saeed et al., 2018; van der Klaauw and Farooqi, 2015). These findings have had diagnostic value for patients and have paved the way for stratified therapy as seen with the treatment of congenital leptin deficiency by recombinant leptin (Farooqi et al., 1999) and of *POMC* and *LEPR* deficiency by the melanocortin 4 receptor (*MC4R*) agonist setmelanotide (Clément et al., 2018; Kühnen et al., 2016).

By focusing on more common forms of genetic variation (minor allele frequency [MAF] > 5%) in population-derived cohorts, genome-wide association studies (GWAS) have identified over 250 loci that are associated with body mass index (BMI) and/or obesity (defined as a BMI > 30 kg/m^2), mostly through modest effects on neuronal genes (Akiyama et al., 2017; Grarup et al., 2018; Justice et al., 2017; Minster et al., 2016; Turcot et al., 2018; Locke et al., 2015). While cumulatively these approaches have provided a framework for understanding the genetic architecture of weight regulation and susceptibility to obesity, a substantial proportion of the heritability of human obesity (including severe childhood-onset obesity) has yet to be explained. There is no biological reason why genetic risk factors for common complex traits/diseases should fall neatly into these two categories, as we (Barroso and McCarthy, 2019) and others (Katsanis, 2016; Marouli et al., 2017) have suggested. We therefore hypothesize that variants that contribute to this "missing heritability" in obesity will include a range of allele frequencies and effect sizes in a continuum of causality (Katsanis, 2016), as seen for other complex traits (Marouli et al., 2017), and explore this in the work we present here.

RESULTS

Rare Variants Implicate Three New Genes in Human Energy Homeostasis

Here, we studied a cohort of European ancestry individuals with severe childhood-onset obesity (SCOOP) in whom known causes of monogenic obesity, such as congenital leptin deficiency and *MC4R* mutations, had been excluded (STAR Methods). Children were recruited into the cohort if they had a BMI standard deviation score (BMI SDS) greater than three and age of onset below 10 years (Wheeler et al., 2013) (STAR Methods). Our study design, focused on early-onset severe obesity, was aimed at increasing power to identify genes with an excess burden of rare, functionally significant variants with moderate to large effects on the phenotype (STAR Methods). We analyzed whole-exome sequencing (WES) data from 927 SCOOP cases (Hendricks et al., 2017; Walter et al., 2015) and 4,057 UK healthy blood donors from the INTERVAL cohort (stage 1; STAR Methods; Figures 1 and S1). To test for different genetic effects, we performed single-variant (Table S1) and three nested gene-based analyses: (1) burden of very rare (MAF < 0.025%) predicted loss-of-function (LOF) variants (LOF analysis), (2) burden of very rare (MAF < 0.025%) predicted deleterious variants by five different *in silico* programs (LOF and missense [STRICT] analysis), and (3) SKAT-O analysis of variants with MAF < 1% and predicted deleterious by a single program (LOF and missense [BROAD] analysis) (Figures 1, S1, and S2; Tables S2A–S2C; STAR Methods).

We identified nine genes with an excess burden of variants from LOF, STRICT, and BROAD analyses in cases versus controls, meeting the arbitrary threshold of $p < 10^{-4}$, which we took forward for stage 2 analysis (Figure 1; STAR Methods; Tables S2A–S2C). Targeted sequencing was performed in an additional unrelated 1,810 SCOOP cases and 2,647 controls from the Fenland cohort (stage 2), and validation of reported variants was undertaken by Sanger sequencing (STAR Methods). Validated variants from stage 1 and stage 2 results were combined in meta-analysis. Directionally consistent association with obesity in stage 2, and stronger p values from stage 1 + 2 in either LOF or STRICT analysis was detected in three genes. For each gene, we highlight the most significant association result: $p_{meta\text{-}LOF} = 1.23 \times 10^{-5}$ *PHIP* (pleckstrin homology domain interacting protein), $p_{meta\text{-}STRICT} = 7.93 \times 10^{-4}$ *DGKI* (diacylglycerol [DAG] kinase iota), and $p_{meta\text{-}LOF} = 3.19 \times 10^{-7}$ *ZMYM4* (zinc-finger-MYM-type-containing 4) (Table 1). *ZMYM4* results are significant after Bonferroni correction for all genes in the genome ($p < 2.5 \times 10^{-6}$). In all, we see strong evidence of winner's curse with much larger odds ratios in stage 1, compared to stage 2 analysis.

In a fourth gene, *ZNF32* (zinc-finger protein 32), very rare predicted deleterious variants were only observed in stage 1 cases ($p_{STRICT} = 2.84 \times 10^{-5}$; Table 1). *ZNF32* is a zinc-finger protein of uncharacterized function and with a ubiquitous expression pattern (Figure S3A). We attempted to gain independent evidence of its role in obesity by generating an engineered mouse mutant; however, homozygous *ZNF32* $^{em1(IMPC)Wtsi}$ do not display any obvious phenotype (data not shown). We present our results here to invite others to explore this gene in their cohorts, but at this stage we feel the evidence is insufficient to link this gene with obesity.

Though not independent, analysis using stage 1 cases and external controls (to increase sample size) also provides support (*PHIP*, $p_{LOF} = 4.9 \times 10^{-4}$; *DGKI*, $p_{STRICT} = 0.10$; *ZMYM4*, $p_{LOF} = 7.10 \times 10^{-5}$; *ZNF32*, $p_{STRICT} = 2.14 \times 10^{-2}$; Table S3; STAR Methods). To further strengthen the evidence for or against the role of these genes in extreme obesity, we examined 431 adults with BMI > 40 kg/m^2 from the UK10K project (approximately equivalent to the BMI > 3 SDs used to define severe obesity in children in SCOOP) and 984 non-overlapping adult controls

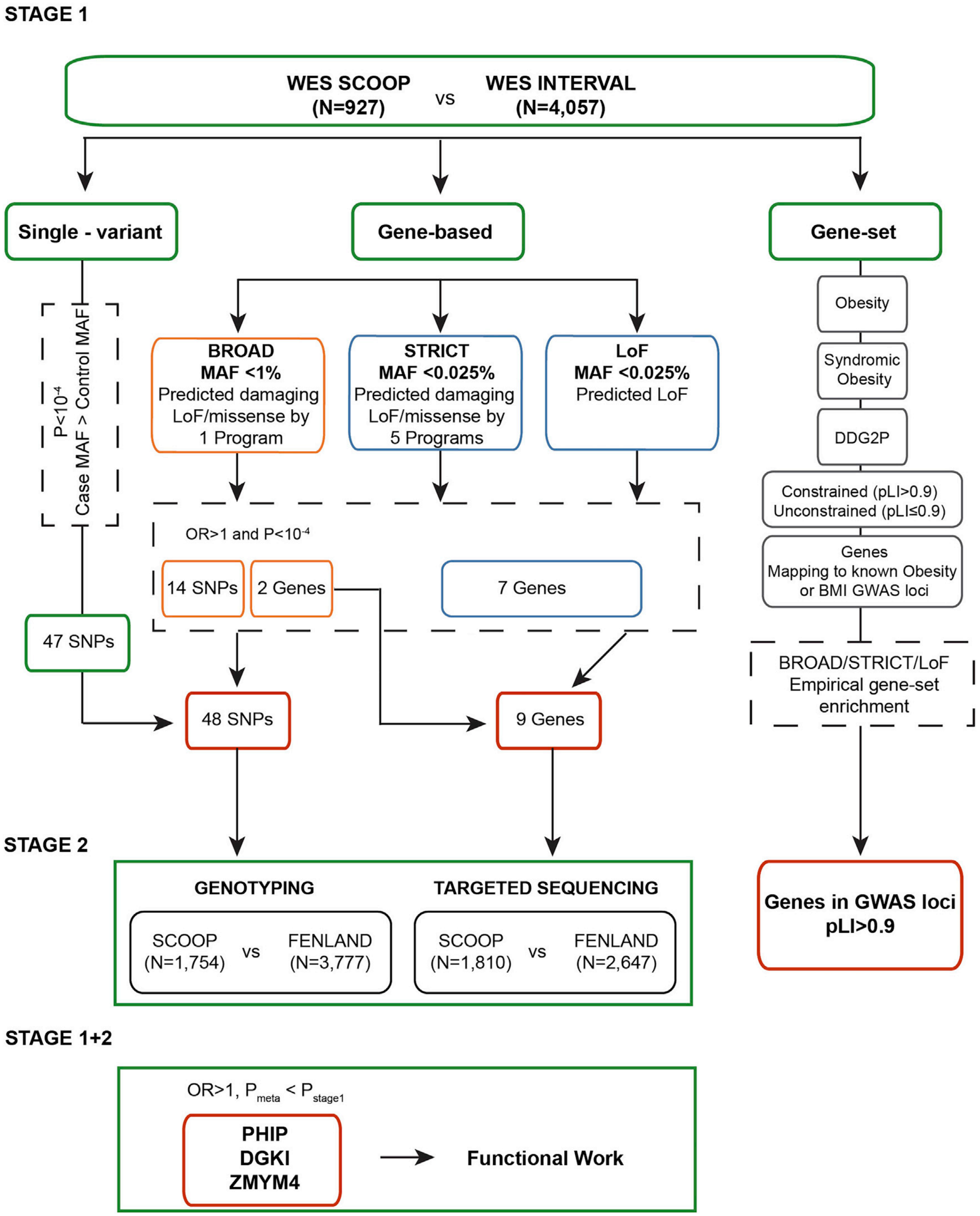

Figure 1. Flow Diagram of Approach and Headline Results

The overall analysis and main results from this study. Whole-exome sequence data from SCOOP and INTERVAL participants were analyzed as single-variant, gene-base, and gene set analyses. Overall, 48 variants from single-variant and gene-based analysis and 9 genes from gene-based analysis were identified and taken forward to stage 2 validation, by genotyping or targeted-sequenced. Combined stage 1 and stage 2 significant results were found in three genes (*PHIP*, *DGKI*, and *ZMYM4*), which were taken for functional follow-up. In parallel, gene set analysis performed on five primary gene sets (obesity, syndromic obesity, DDG2P, Constrained [pLI > 0.9], Unconstrained [pLI $\leq$ 0.9], and Genes Mapping to known Obesity or BMI GWAS loci) is represented. The strongest enrichment, which was detected in loss-of-function constrained genes (pLI > 0.9) mapping to obesity or BMI-associated GWAS loci, is shown (Genes in GWAS loci, pLI > 0.9). Related to Figure S1.

Table 1. Burden of Very Rare Variants (MAF < 0.025%) Enriched in Obese Cases Compared to Controls

Gene	Test	Stage 1 p Value	Stage 1 Number of Variants/Case Alleles/Control Alleles	Stage 1 OR (adj OR [95% CI])	Stage 2 p Value	Stage 2 Number of Variants/Case Alleles/Control Alleles	Stage 2 OR (adj OR [95% CI])	Stage 1 + Stage 2 p Value	Stage 1 + Stage 2 OR (adj OR [95% CI])
PHIP	STRICT	3.67×10^{-5}	13/9/6	6.57 [2.34–18.45]	0.2070	8/5/3	2.45 [0.59–10.27]	9.81×10^{-5}	4.35 [1.88–10.06]
	LOF	2.84×10^{-5}	4/4/0	Inf (39.41 [2.12–732.02])	0.0361	3/3/0	Inf (10.26 [0.53–198.57])	1.23×10^{-5}	Inf (39.74 [2.27–695.83])
DGKI	STRICT	4.95×10^{-6}	9/7/2	15.32 [3.18–73.77]	0.5883	7/4/4	1.47 [0.37–5.86]	7.93×10^{-4}	4.81 [1.78–13.01]
	LOF	0.0364	1/1/0	Inf (13.13 [0.53–322.53])	0.2265	1/1/0	Inf (4.44 [0.18–109.07])	0.0243	Inf (12.36 [0.59–257.51])
ZMYM4	STRICT	0.0160	12/7/10	3.06 [1.17–8.05]	0.1934	6/4/2	2.93 [0.54–16.02]	0.0068	2.70 [1.19–6.12]
	LOF	2.94×10^{-7}	5/6/0	Inf (56.93 [3.21–1010.63])	0.2265	1/1/0	Inf (4.39 [0.18–107.89])	3.19×10^{-7}	Inf (53.48 [3.05–936.37])
ZNF32	STRICT	2.84×10^{-5}	4/4/0	Inf (39.41 [2.12–732.02])	–	0/0/0	–		
	LOF	0.0364	1/1/0	Inf (13.13 [0.53–322.53])	–	0/0/0	–		

Stage 1 included 927 SCOOP obesity cases and 4,057 INTERVAL controls; stage 2 included 1,810 SCOOP obesity cases and 2,647 Fenland controls. Test indicates whether it was a gene burden with very rare variants predicted to be LOF (LOF), or whether it included LOF as well as missense variants predicted deleterious by five *in silico* programs (STRICT). For infinite odds ratio (Inf), we provide an adjusted OR estimate by adding 0.5 to each of the contingency table cells (adj OR). Related to Tables S2 and S6. Details of variants included in the tests are in Table S6C.

from the 1958 Birth Cohort (STAR Methods). We identified between 0 and 2 STRICT or LOF very rare variants in these four genes in adult cases and 0–3 STRICT or LOF very rare variants in adult controls, and while the 95% CIs overlapped estimates from children, they also included the null (Table S4). Single-variant and BROAD stage 1 + 2 combined results did not yield additional significant association results (Tables 2, S5, and S6A; STAR Methods).

Finally, through analysis focused on 43 genes previously known to harbor mutations causal of monogenic/syndromic obesity (Table S8A), we find 12 genes have nominal evidence of a burden of BROAD, STRICT, or LOF very rare variants in our obese cases compared to controls (stage 1 samples, $p < 0.05$; Table S2D).

PHIP Variants Are Associated with Obesity with and without Development Delay

PHIP is of particular interest, as deletions and frameshift mutations in this gene have been reported in patients with developmental delay, intellectual disability, and dysmorphic features, and in some cases, patients were reported to be overweight (de Ligt et al., 2012; Jansen et al., 2018; Webster et al., 2016). In keeping with previous reports, some probands in SCOOP had learning difficulties and dysmorphic features (Table 3). Repeat analysis of our obesity cases stratified by the presence/absence of developmental delay demonstrated a very strong association of a burden of *PHIP* very rare LOF variants with obesity in the presence of developmental delay ($OR_{LOF\_stage1+2} = 95.01$, $CI_{95} = 5.11,1765.21$, $p_{LOF\_stage1+2} = 3.19 \times 10^{-10}$; Figure 2). We also found moderate evidence for association with obesity in the absence of developmental delay ($OR_{LOF\_stage1+2} = 26.95$, $CI_{95} = 1.39,521.79$, $p_{LOF\_stage1+2} = 0.0006$; Figure 2; Table S7). Where samples from one or both parents were available, we found that 3 probands had inherited the variant from an overweight/obese parent (A389T, R409C, and T1506A), 3 variants (R250X, F1414S, and c.3536-4_3540delTTAGATATT) were found *de novo*, and one variant (found in two unrelated probands) was inherited from a normal weight parent (T1506A) (Table 3). The absence of severe obesity in some family members carrying *PHIP* STRICT missense and LOF variants, the absence of developmental delay in some probands with LOF mutations (Table 3), and the presence of STRICT missense variants in control participants without obesity (6/9 controls who were carriers of very rare STRICT variants had a BMI < 30 kg/m^2) suggest variable penetrance.

PHIP Regulates *POMC* Transcription in Cells

We performed experiments in cells to explore the potential mechanisms by which *PHIP* might regulate body weight and to test the functional consequences of the 17 very rare coding variants found in cases and controls (Figure 3A). Human PHIP exists as two isoforms with distinct cellular localizations (Figure 3B). A short cytoplasmic PHIP (104 kDa) isoform interacts with insulin receptor substrate (IRS)-1 and -2 and is required for insulin and insulin-like growth factor (IGF-1) signaling (Farhang-Fallah et al., 2000). A long (230-kDa) PHIP isoform is exclusively localized in the nucleus. Nuclear PHIP (synonyms DDB1- and CUL4-associated factor 14 [DCAF14] or replication initiation determinant protein [REPID]) is known to bind directly to

Table 2. Burden of Rare Variants (MAF < 1%) from BROAD Analysis Enriched in Obese Cases Compared to Controls

	Stage 1			Stage 2			Stage 1 + Stage 2	
Gene	p Value	Number of Variants/Case Alleles/Control Alleles	OR	p Value	Number of Variants/Case Alleles/Control Alleles	OR	p Value	OR
PHIP	0.0059	38/14/29	2.11 [1.12–4]	0.0086	22/18/9	2.95 [1.33–6.57]	4.58×10^{-4}	2.4 [1.5–3.84]
DGKI	0.0134	30/18/40	1.97 [1.13–3.44]	0.8910	27/14/20	1.03 [0.52–2.03]	0.0998	1.35 [0.88–2.07]
ZMYM4	0.071	34/25/70	1.56 [0.99–2.47]	0.4911	25/57/89	0.94 [0.68–1.31]	0.4493	1.38 [1.06–1.8]
ZNF32	0.0033	6/4/2	8.76 [1.6–47.81]	1.0000	3/2/3	0.98 [0.16–5.85]	0.0869	3.53 [1.08–11.58]

Stage 1 included 927 SCOOP cases and 4,057 INTERVAL controls; stage 2 included 1,810 obesity cases and 2,647 Fenland controls.

chromatin to promote initiation of DNA replication and gene transcription (Jang et al., 2018). It also mediates effects on post-natal growth (Li et al., 2010), β cell growth, regulation, and survival (Podcheko et al., 2007). Three variants affected splice donor/acceptor sites and were predicted *in silico* to lead to exon skipping/intron retention and result in LOF of the long isoform of *PHIP* (Figure 3A).

Here, using immunoprecipitation, we showed that cytoplasmic PHIP interacts with IRS-2 in cells stimulated with leptin (Figure S4A); however, this interaction is not required for and does not modulate leptin-mediated phosphorylation of STAT3 or ERK1/2, key signaling pathways involved in energy homeostasis (Figure S4B). We next investigated whether nuclear PHIP could directly affect the transcription of pro-opiomelanocortin (POMC), a neuropeptide that mediates the appetite-suppressing effects of leptin. Using a POMC luciferase reporter assay, we found that wild-type (WT) PHIP potentiated POMC transcription in the absence of leptin (Figure 3C); all PHIP mutants decreased POMC transcription (Figure 3D). Seven mutants repressed POMC transcription below levels seen for the null mutation R250X. In co-transfection experiments with varying concentrations of WT and mutant PHIP, four of these mutants (T289P, D594E, Q1343X, and R1505Q) exert a dominant-negative effect, repressing POMC transcription by WT PHIP in a dose-dependent manner (Figure 3E). Using fluorescent microscopy, we found that most mutants did not alter cellular localization of PHIP, although variants found in cases led to a significant decrease in the ratio of nuclear:cytoplasmic PHIP compared to controls ($p = 0.004$) (Figures 3F–3H, S4, and S5). This finding suggests that a reduction in the amount of nuclear PHIP available to enhance POMC transcription (with or without leptin) may contribute to the development of obesity. Leptin stimulation rescued the effects of some, but not all, PHIP mutants on POMC transcription (Figure S4).

PHIP Variant Carriers Exhibit Maladaptive Behaviors

Phip null mice exhibit a 40% growth deficit by weaning, develop hypoglycemia, and do not survive beyond 4–5 weeks (Li et al., 2010). In keeping with the growth phenotype seen in null mice, 5 of 13 *PHIP* variant carriers on whom data was available (Table 3) were born with low birthweight for gestational age. In some cases, there was evidence of rapid catch-up growth in early childhood (R409C), whereas other probands remained short (Q1343X) (Table 3). In addition, some variant carriers reported hyperphagia and developed insulin resistance and early type 2 diabetes (Table 3). Maladaptive behaviors, reminiscent of those seen in carriers of variants in *SH2B1* (Doche et al., 2012), another PH domain-containing protein involved in leptin and brain-derived neurotrophic factor (BDNF)-mediated signaling, were reported in several probands in this study and in previous clinical case series. Taken together, the combined genetic and functional data provide compelling evidence that *PHIP* is involved in human obesity with and without developmental delay. *PHIP* variants are likely to impact the transcription of multiple downstream target genes, which may in part explain the variability in clinical phenotype observed, which is not simply explained by the results of functional assays used.

DGKI Is Associated with Obesity in Humans and Fat Mass in Mice

Next, we studied *DGKI*, which is expressed in numerous brain regions (hippocampus, hypothalamus, caudate nucleus, and cortex) and in the thyroid (STAR Methods; Figure S3B), making it a plausible DGK isoform to be involved in energy homeostasis and metabolism. DAG kinases terminate DAG signaling and are important regulators of long-term potentiation and long-term depression, cellular mechanisms involved in synaptic plasticity (Lee et al., 2016). Common variants in the *DGKI* gene region have been associated with dyslexia (Matsson et al., 2011); the patient with the nonsense mutation (Q265X) was reported to have speech and language delay (Table 3). Delayed habituation to novel environments has previously been reported in *Dgki* knockout mice (Yang et al., 2011).

Here, we engineered mutant $Dgki^{em1(IMPC)Wtsi}$ mice, and utilizing the Sanger Institute mouse phenotyping pipeline we provide preliminary evidence supporting a role of *Dgki* in energy homeostasis in mice (STAR Methods). Phenotyping of homozygous $Dgki^{em1(IMPC)Wtsi}$ mice suggests these mice have increased fat mass and fat percentage, lower bone mineral density, and higher plasma glycerol (males) (STAR Methods; Figures S6 and S7). However, metabolic phenotyping after exposure to a high-fat diet will be required to further investigate the impact of this gene deletion in energy homeostasis and to gain mechanistic insights.

ZMYM4 Is a Novel Gene Linked to Human Obesity

ZMYM4 encodes a poorly characterized protein with a predicted central zinc-finger domain, a proline-rich region, and a

Table 3. Phenotypes Seen in Carriers of Rare Variants in *PHIP*, *DGKI*, and *ZMYM4*

Variant	Age (Years)	Sex	BMI (SDS)	Height (SDS)	Bwt SDS	Learning Difficulties	Dysmorphic Features	Autistic Features	Hyperactivity	Aggression	Anxiety or Depression	Insulin (pmol/L)	Glucose (mmol/L)
PHIP													
R250X[a]	6	F	25 (3.6)	111 (−0.9)	0.6								
T289P	7.7	F	34 (4.2)	133 (1.3)	1.5	✓	✓					134	3.8
A389T	5.1	F	40 (6.0)	114 (1.1)	2.2							146	4.7
A389T[b]	46	M	54	173								171	5.3
R409C	1.4	F	24 (3.7)	84 (1.6)	−2.9	✓		✓		✓	✓	47	5.1
R409C[b]	34	M	38	175									
R721X	44.3	F	74	150								@	9.1
Q1343X	17.2	F	43 (3.8)	151 (−2.1)	−0.1	✓			✓			208[c]	5.9
F1414S[a]	20.7	M	40	188	−0.9	✓	✓	✓	✓	✓		398[c]	6
K1443T fsX11	7.6	F	35 (4.3)	119 (−1.1)	1.7	✓					✓	42	4.4
T1506A	2.4	F	23 (3.8)	102 (3.8)	1.6						✓	292	5.8
T1506A[b]	44	M	21										
T1506A	15	M	46 (4.0)	168 (−0.1)	0					✓		98	5.1
T1506A[b]	54	M	29										
R1718S	22.7	M	40	170	−1.8							100	5.4
c.823-2A>G	7.9	M	27 (3.5)	135 (1.4)	0.3	✓	✓	✓				57	5.5
c.1524+1G>T	15.5	M	35 (3.2)	176 (0.6)	0.23	✓	✓					154	5.2
c.3536-4_3540 delTTA GATATT[a]	13.1	F	49 (4.2)	161 (0.7)	−0.9	✓						144	5.3
DGKI													
D833N	15.3	F	45 (4.0)	166 (0.5)	−0.2							144	4.6
L430R	9.1	F	26 (2.9)	132 (−0.2)	1.3								
Q265X	3.6	M	22 (3.6)	113 (3.3)	−0.8	✓σ						32	4.7
L192F	12.6	M	31 (3.1)	151 (−0.1)	−0.1							41	3.1
ZMYM4													
N89S	15.5	F	39 (3.5)	161 (−0.4)	0.1	✓						136	4.6
K163R fsx10	11.1	M	28.3 (3.0)	152 (1.2)	0.1							24	4.2
R379C	1.2	F	26 (4.5)	84 (3.0)	0.5							36	
K387N	7.8	M	25 (3.3)	131 (0.8)	0.6	✓						30	3.9

M, male; F, female. BMI (kg/m^2) shown for adults with age- and gender-adjusted standard deviation scores (SDS) shown in brackets for children under 18 years of age at referral. Height in cm (SDS). Birthweight (Bwt) SDS adjusted for gestational age. ✓ indicates reported presence of a phenotype. Fasting values for glucose and insulin reported: normal range for fasting plasma insulin = 0–60 pmol/L. @, on medication for type 2 diabetes. Speech and language delay represented as ✓σ.

[a]*De novo* inheritance established by genotyping both parents.

[b]Rare coding in family member.

[c]Presence of acanthosis nigricans (skin marker of insulin resistance).

C-terminal DUF3504 domain, the latter suggesting it may function as a transcriptional activator or repressor (Kojima and Jurka, 2011). It has a broad tissue expression pattern (STAR Methods; Figure S3C), is predicted to be an LOF intolerant gene (pLi = 1.00) (Lek et al., 2016), and has not been previously linked to obesity or metabolism. LOF intolerant genes are those in which strong negative selection has meant that the gene has fewer LOF mutations in the general population than expected, presumably because of their impact on reproductive fitness. Other than mild learning difficulties, there were no distinctive phenotypes other than severe obesity in variant carriers (Table 3).

To gain further insight into *ZMYM4* function, we generated mice homozygous for the *Zmym4*$^{em1(IMPC)Wtsi}$ allele. In keeping with *ZMYM4* being an LOF intolerant gene, we found that homozygous *Zmym4*$^{em1(IMPC)Wtsi}$ mice are pre-weaning lethal. No homozygous mice were detected from 56 offspring, while 14 from 56 offspring

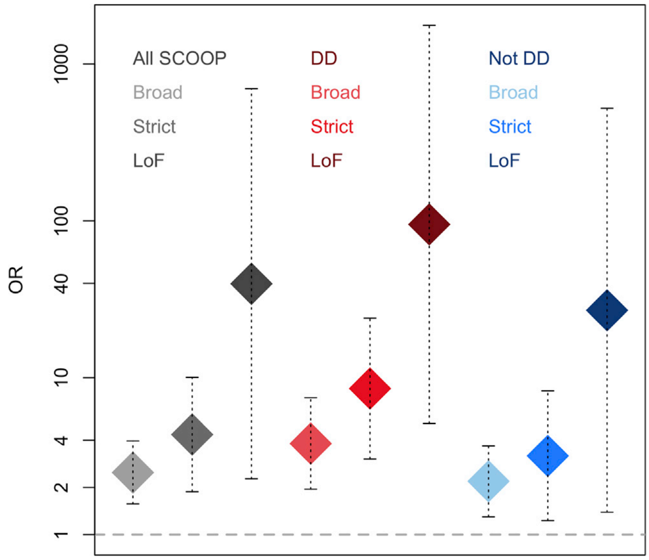

Figure 2. Detailed *PHIP* Association Results

Results for BROAD, STRICT, and LOF analyses for *PHIP* variants are shown overall (gray), in obese patients with developmental delay (red), and in obese patients without developmental delay (blue). ORs are represented by diamonds with 95% CI shown by dashed lines. Across all analyses, there is a trend for greater ORs for increasing stringency of the test (BROAD < STRICT < LOF). Related to Table S7.

would be expected (Fisher's exact test, $p < 0.0001$; STAR Methods). More sophisticated models targeting allele loss in specific tissues or detailed phenotyping in heterozygous *Zmym4*$^{em1(IMPC)Wtsi}$ mice will be required to further elucidate the physiological role of this gene.

Genes in BMI GWAS Loci Are Enriched for Very Rare Predicted Deleterious Variants in Severe Obesity

Lastly, we investigated whether particular groups of genes, as a gene set, were enriched in very rare predicted deleterious variants in severe early-onset obesity cases compared to controls. These analyses can overcome some of the power limitations when testing genes individually: though individual genes may have insufficient evidence of association with severe early-onset obesity, collectively, gene sets may be shown to associate with obesity. We performed analyses on 10 primary gene sets (Table S8; STAR Methods).

In gene set analyses, we found that the set of 157 genes mapping to BMI and obesity GWAS loci (GWAS set) was enriched for very rare functional variants in childhood obesity cases (OR_{STRICT} = 1.18, CI_{95} = 1.03,1.18, $p_{perm\text{-}STRICT}$ = 1.63×10^{-2}; OR_{LOF} = 1.39, CI_{95} = 1.07,1.80, $p_{perm\text{-}LOF}$ = 1.39×10^{-2}; Figure 4A; Table S9A). This provides a compelling rationale for sequencing this group of genes in additional cases and controls, to identify novel variants that may have stronger effects on severe early-onset obesity, and which may yield potential novel drug targets.

We also noted that genes that are normally depleted in LOF mutations in the population, known as LOF intolerant genes (pLI > 0.9) (Lek et al., 2016), were more common in this GWAS set compared to all genes in the genome (33.8% versus 19.1%, $p_{ChiSq\text{-}Ind}$ = 3.11×10^{-6}). In secondary analyses, we found that this subset of 53 LOF intolerant genes among the 157 genes in the GWAS set was further enriched in very rare deleterious variants in our obese cases compared to controls (OR_{STRICT} = 1.34, CI_{95} = 1.14,1.58 $p_{perm\text{-}STRICT}$ = 8.00×10^{-4}; OR_{LOF} = 3.54, CI_{95} = 1.80,6.95, $p_{perm\text{-}LOF}$ = 5.00×10^{-4}) (Figure 4A; Table S9D). The OR for putative LOF variants in this gene set of 53 genes was similar to the estimate for the gene set of monogenic obesity genes (Figure 4A). This suggests that some of these genes may harbor rare variants of potentially larger effect size affecting severe childhood obesity.

LOF Intolerant Genes Are Enriched in Very Rare Deleterious Variants in Obese Cases with Developmental Delay

As severe obesity is not normally reproductive lethal, it was surprising to find that 53 LOF intolerant genes among the GWAS set were particularly enriched for very rare deleterious variants in our cases. To further explore these results, we stratified our cases into those with developmental delay and those without. In this group of 53 LOF intolerant genes in the GWAS set, enrichment was similar whether or not obese cases had developmental delay (Figure 4B; Table S9D).

Next, we noted that the set of all 3,488 LOF intolerant genes in the genome (pLi > 09-set) was also significantly enriched in rare variants in obesity cases (OR_{STRICT} = 1.07, CI_{95} = 1.02,1.13, $p_{perm\text{-}strict}$ = 6.85×10^{-3}; OR_{LOF} = 1.17, CI_{95} = 1.07,1.27, $p_{perm\text{-}LOF}$ = 3.00×10^{-4}; Figure 4A; Table S9A). However, consistent with obesity not normally being reproductive lethal, we find most of this signal to be driven by obesity cases that also have developmental delay (Figure 4B; Tables S9B and S9C), suggesting these genes may yield further diagnostic findings in this set of patients. In contrast, genes that are not LOF intolerant ($pLi \leq 0.9$) show no evidence of enrichment (Figures 4A and 4B; Tables S9A–S9C).

Rare Genetic Variation Associated with Obesity Is in the Most Constrained Gene Sets

Many diseases that are under strong negative selection, such as schizophrenia, have been shown to be enriched for rare, putative deleterious genetic variation in genes that are usually constrained to LOF variation (pLi > 0.9). Given that obesity may not have undergone strong negative selection, we aimed to test whether the contribution of rare, putative deleterious genetic variation was also observed in gene sets regardless of the level of constraint against deleterious variation. To test this, we assessed gene set enrichment among the deciles of missense constrained genes where genes in the top decile are more constrained against missense variation (i.e., less likely to have missense variation in the general population) and genes in the first decile are less constrained to missense variation (i.e., more likely to have missense variation in the general population). We additionally assessed the deciles of LOF constrained genes. We did not find strong evidence of enrichment for rare deleterious variants beyond gene sets with the strongest constraint against missense or LOF variation, suggesting that, at least for extreme clinical obesity such as that studied here, enrichment for rare putative deleterious variants is primarily in genes that

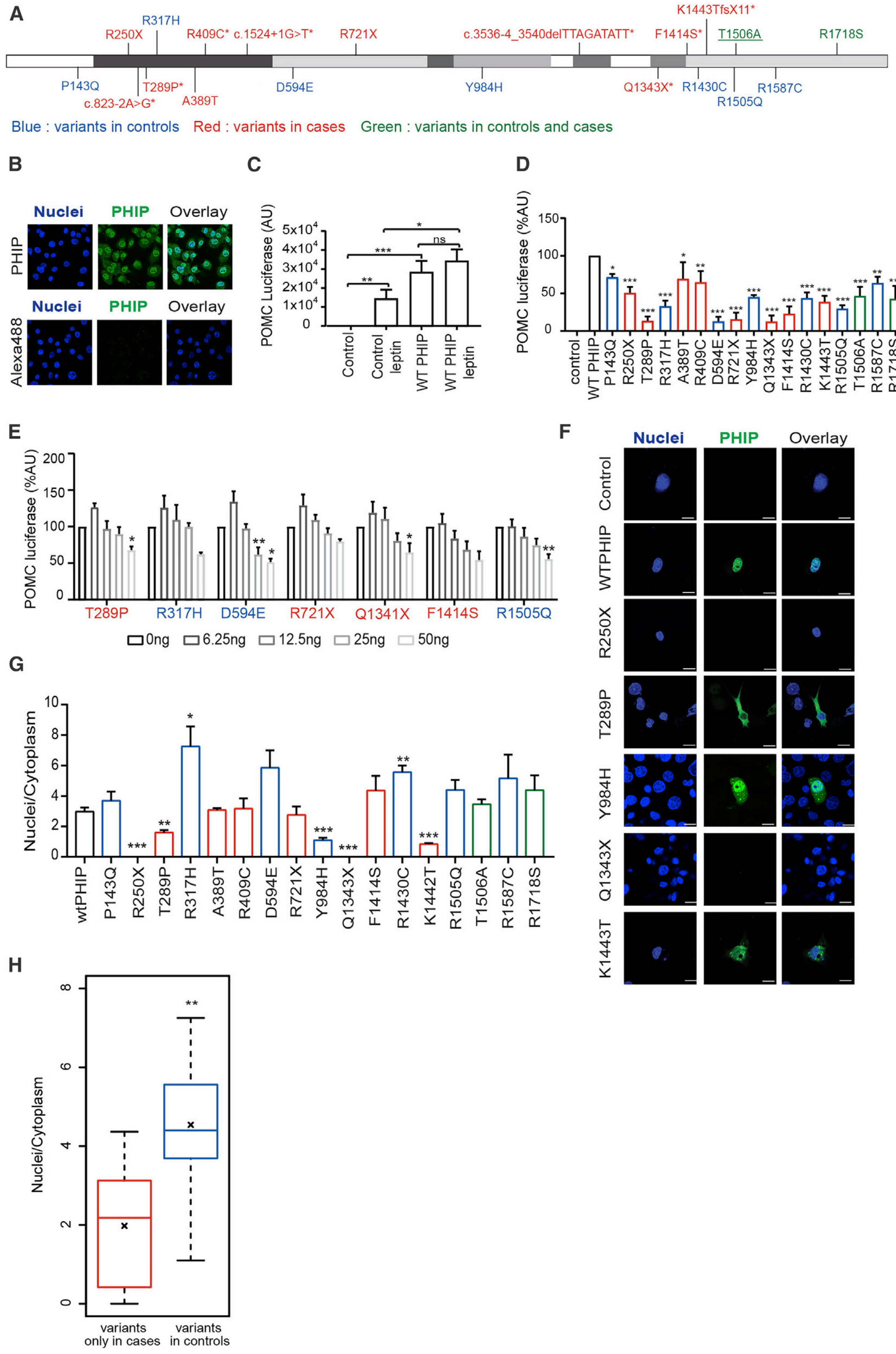

Figure 3. Functional Characterization of Obesity-Associated Variants in *PHIP*

(A) Mapping of human *PHIP* coding and splice-site variants identified in cases only (red), controls only (blue), and in cases and controls (green) on the full-length *PHIP* isoform. One variant was found in two unrelated probands (underlined); some variants were found in probands who also had developmental delay (*).

(legend continued on next page)

CellPress
OPEN ACCESS

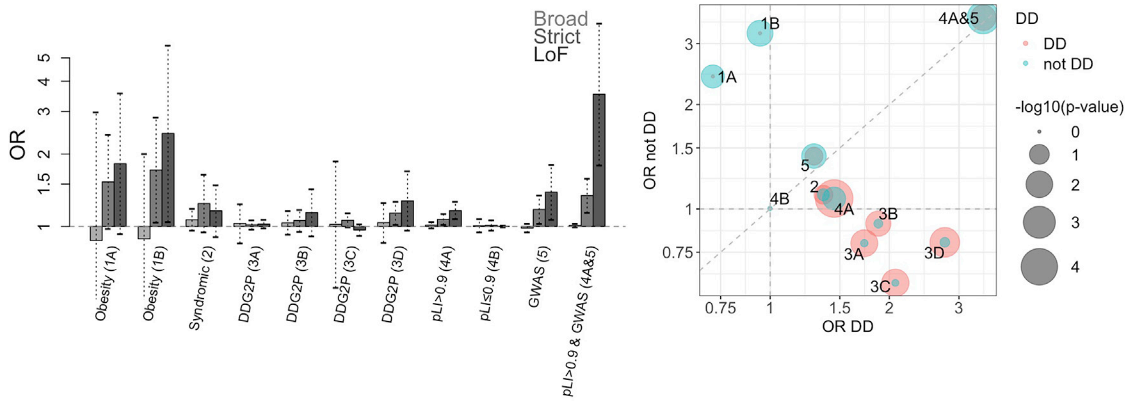

Figure 4. Gene Set Analysis

(A) Gene set enrichment analysis for a burden of BROAD, STRICT, and LOF variants in cases compared to controls across 10 primary gene sets. Obesity 1A (monogenic obesity genes, 11 genes), Obesity 1B (9 genes, monogenic obesity genes where *LEP* and *MC4R* were removed), Syndromic 2 (syndromic obesity genes, 32 genes), DDG2P 3A (developmental disorder dominant genes causal through missense or LOF mutations, 360 genes), DDG2P 3B (developmental disorder dominant genes causal through LOF mutations 274 genes), DDG2P 3C (developmental disorders with brain abnormalities dominant genes causal through missense or LOF mutations, 187 genes), DDG2P 3D (developmental disorders with brain abnormalities dominant genes causal through LOF mutations 147 genes), pLI > 0.9 4A (LOF intolerant genes, 3,488 genes), pLI $\leq$ 0.9 4B (LOF tolerant genes, 14,753 genes), GWAS 5 (genes mapping to BMI/obesity GWAS loci based on GWAS catalog data, 157 genes). Results for a combined subcategory of pLI > 0.9 and GWAS (4A and 5, 53 genes) are also shown. Gene sets are described in Table S8 and detailed gene set results are in Table S9. Odds ratios (ORs, y axis) and 95% CI (dashed lines) are shown for each gene set and analysis.

(B) Odds ratios (ORs) for obesity cases with developmental delay (DD, x axis) and ORs for obesity cases without developmental delay (not DD, y axis) are shown for each of the gene sets shown in (A). p value strength is indicated by size of the circle, for obesity DD (pink) and obesity not DD (blue) in semi-transparent circles. LOF intolerant genes overall (4A) and all of DDG2P (3A–3D) have larger ORs and are more significantly associated with obesity DD. In contrast, GWAS genes that are also LOF intolerant (4A&5) show equivalent ORs and statistical association with obesity DD and obesity not DD. Related to Tables S8 and S9.

are strongly constrained against deleterious variation (Table S9E).

DISCUSSION

Most genetic studies of obesity have to date identified very rare, likely fully penetrant monogenic mutations causal of severe forms of obesity, or common low-impact alleles influencing BMI and other measures of adiposity in large population-based cohorts. Here, by focusing our efforts on a clinical cohort of very severe early-onset obesity cases of unknown cause, and combining genetic evidence with functional work in cells and *in vivo*, we provide evidence for a continuum of causality in the genetic architecture of obesity. We identify three genes (*PHIP*, *DGKI*, and *ZMYM4*) newly implicated in obesity, harboring very rare predicted deleterious alleles, with intermediate effects and penetrance between those identified through family or large-population scale efforts.

These findings have potential diagnostic implications. While *PHIP* has previously been linked to a complex syndrome of developmental delay, and some patients have been noted to be overweight, the gene is not currently included on any gene panel for diagnostic testing in obesity, nor is *PHIP* screening recommended in obesity syndromes. The patients we identified all presented with severe obesity—some did have developmental delay, but not all. We consider that this finding plus the associated molecular work identifying a mechanism by which disruption of *PHIP* can cause obesity (by disrupting transcription of *POMC*) establishes that some patients may present with obesity alone. While we find a broad spectrum of mutations, this alone cannot explain the divergence in phenotypic spectrum seen. It is likely that as *PHIP* affects transcriptional regulation, specific mutations may exert a variable effect clinically. Our data suggest that *PHIP* should be included in genetic testing recommended in clinical guidelines as part of the assessment of severe childhood-onset obesity, particularly in the presence

(B) Representative confocal fluorescence microscopy images showing PHIP localization in the nucleus and the cytoplasm of COS7 cells. Blue, nuclei; green, antibody against endogenous PHIP. Scale bar, 100 μm.

(C) Overexpression of WT PHIP potentiates POMC transcription in basal and leptin-stimulated conditions. n = 15, mean + SEM, two-tailed unpaired t test with Welch's correction, *p < 0.05, **p < 0.01, ***p < 0.001.

(D) Effect of human PHIP mutants on POMC transcription compared to WT in basal conditions. n = 4–6, mean + SEM, ordinary one-way ANOVA with multiple comparisons to WT, *p < 0.05, **p < 0.01, ***p < 0.001.

(E) Dominant-negative effect of human PHIP mutants in POMC transcription in basal conditions. n = 3, mean + SEM, ordinary one-way ANOVA with multiple comparisons to 0 ng, *p < 0.05, **p < 0.01, ***p < 0.001.

(F) Representative confocal fluorescence microscopy images showing cytoplasmic protein localization of PHIP mutants in basal conditions.

(G) Nuclei to cytoplasm ratio of PHIP subcellular localization. n = 3, mean + SEM, two-tailed unpaired t test with Welch's correction, *p < 0.05, **p < 0.01, ***p < 0.001.

(H) Nuclear to cytoplasmic ratio in variants found only in cases in comparison to controls. Lines represent medians and crosses represent means. Dashed lines span from the minimum to maximum values. Wilcoxon rank-sum test, **p = 0.004. Related to Figures S4 and S5.

of developmental delay (Styne et al., 2017). These findings may also inform the mechanism-based treatment of *PHIP* variant carriers with a melanocortin receptor agonist currently in clinical trials, which leads to significant weight loss in severe obesity due to complete POMC deficiency (Kühnen et al., 2016). A subset of *PHIP* variant carriers may also benefit from treatment with recombinant leptin, leptin mimetics, or sensitizers.

Little is known of the biological function of *DGKI* or *ZMYM4*, or the mechanism through which they influence energy homeostasis. Their discovery is therefore important as it provides new avenues for future biological exploration, and establishes a new link between these genes and as yet undescribed molecular pathways implicated in human metabolism and energy homeostasis.

Our results also demonstrate the challenge in identifying novel molecular links between heterogeneous complex diseases and very rare variants that may not be fully penetrant. From a statistical standpoint, the burden of proof is high, possibly requiring in the order of tens of thousands of cases to attain established Bonferroni multiple-testing correction thresholds (Zuk et al., 2014). Nonetheless, failure to meet the stringent Bonferroni threshold does not preclude the possibility of interesting and relevant results, especially when combined with other functional evidence, which is what we have sought to do to increase confidence in our findings. This challenge has been similarly documented in other heterogeneous disorders such as autism (Yuen et al., 2015; C Yuen et al., 2017) and schizophrenia (Singh et al., 2017), where single genes with a burden of rare damaging variants in cases compared to controls have also not attained the required statistical burden of proof. Nonetheless, the results of those studies have provided important insights into the genetic etiology of those disorders. Namely, in the schizophrenia study, Singh and colleagues demonstrated that a group of 3,488 genes previously shown to be intolerant to LOF mutations were enriched with a burden of rare deleterious variants in cases, identifying this group of genes as a whole as important in schizophrenia.

This result is analogous to our gene set analyses findings. We provide for the first time evidence that a group of 157 genes mapping nearest to BMI-associated GWAS loci are enriched in very rare deleterious variants in our cases. Furthermore, we demonstrate that a subset of 53 out of those 157 genes (that have evidence of being LOF intolerant genes) is even more enriched in very rare functional variants in obese cases, compared to controls achieving effect estimates similar in size to the group of well-established monogenic obesity genes. This set of 53 genes is therefore a primary target to study in more detail in future large-scale obesity sequencing studies.

Last, we demonstrate that collectively the 3,488 genes intolerant to LOF (pLi > 0.9-set) are enriched in very rare predicted deleterious variants in severe early-onset obese patients who also have developmental delay, suggesting that further novel discoveries that are clinically relevant may be made by studying this particular subgroup of individuals and genes.

Limitations of Study

Our study has a number of limitations. First, all the newly described genes linked to childhood obesity are driven mostly from our discovery dataset and, aside from *ZMYM4*, neither *PHIP* nor *DGKI* meet Bonferroni correction for all genes in the genome. Consequently, these findings merit replication in additional cases with severe childhood-onset obesity and also in adult obesity cases and other ancestries, to further investigate the impact of these genes in obesity more broadly. Second, while we observe enrichment of very rare predicted deleterious variants in *PHIP*, in cases with severe childhood obesity in the absence of developmental delay, this observation merits additional investigation in additional obesity cases (with and without developmental delay) to identify possible genotype-phenotype correlations. Third, the *DGKI* mouse exhibits increased fat mass only in males; further work is therefore needed to evaluate the apparent gender dimorphism, and to gain insights into the mechanism underlying the effect on body composition we observe. Overall, our results highlight the challenges in robustly linking very rare variants with incomplete penetrance to a complex and heterogeneous phenotype such as obesity.

To conclude, we demonstrate that WES in clinically ascertained severe-childhood onset obesity, and follow-up in additional unrelated cases, identifies genes and gene sets newly linked to obesity. Further investigation of the molecular mechanisms affected by rare obesity-associated variants in cells, model organisms, and humans may identify and validate potential targets for weight loss therapy.

STAR★METHODS

Detailed methods are provided in the online version of this paper and include the following:

- KEY RESOURCES TABLE
- RESOURCE AVAILABILITY
  - Lead Contact
  - Materials Availability
  - Data and Code Availability
- EXPERIMENTAL MODEL AND SUBJECT DETAILS
  - Human Studies
  - Samples
  - Childhood Obesity Cases
  - Adult Obesity Cases
  - Population Controls
  - Mice
- METHOD DETAILS
  - Cell Culture
  - Cloning of PHIP Human Variants
  - Subcellular Localization of Endogenous PHIP
  - Luciferase POMC Transcription Activation Assay
  - Dominant Negative Luciferase POMC Transcription Activation Assay
  - Subcellular Localization of Human PHIP Variants
  - *In Vitro* Immunoprecipitation Assay
  - Western Blotting
- QUANTIFICATION AND STATISTICAL ANALYSIS
  - Sequencing
  - Stage 1 Data
  - Stage 2 Data
  - Validation Sanger Sequencing
  - Adult Obesity Cases and 1958BC Data
  - Variant Annotation

- Single-variant Association Analyses
- Gene-based Association Analyses
- Genotyping
- Gene Set Enrichment
- Gene Sets
- Functional Work Statistical Analysis
- Mouse Statistical Analysis
- Expression Data

SUPPLEMENTAL INFORMATION

Supplemental Information can be found online at https://doi.org/10.1016/j.cmet.2020.05.007.

ACKNOWLEDGMENTS

The authors would like to thank the patients and their families for their participation as well as the physicians who recruited patients to the Genetics of Obesity Study (GOOS). Participants in the INTERVAL randomized controlled trial were recruited with the active collaboration of NHS Blood and Transplant England (https://www.nhsbt.nhs.uk/), which has supported field work and other elements of the trial. DNA extraction and genotyping were co-funded by the National Institute for Health Research (NIHR), the NIHR BioResource (https://bioresource.nihr.ac.uk/), and the NIHR (Cambridge Biomedical Research Centre at the Cambridge University Hospitals NHS Foundation Trust). Sequencing was supported by the Wellcome Trust grant number 206194. The academic coordinating center for INTERVAL was supported by core funding from NIHR Blood and Transplant Research Unit in Donor Health and Genomics (NIHR BTRU-2014-10024), UK Medical Research Council (MR/L003120/1), British Heart Foundation (SP/09/002, RG/13/13/30194, and RG/18/13/33946), and the NIHR (Cambridge Biomedical Research Centre at the Cambridge University Hospitals NHS Foundation Trust). A complete list of the investigators and contributors to the INTERVAL trial is provided in Di Angelantonio et al. (2017). The academic coordinating center would like to thank blood donor center staff and blood donors for participating in the INTERVAL trial. The views expressed are those of the authors and not necessarily those of the NHS, the NIHR, or the Department of Health and Social Care. This work was also supported by Health Data Research UK, which is funded by the UK Medical Research Council, Engineering and Physical Sciences Research Council, Economic and Social Research Council, Department of Health and Social Care (England), Chief Scientist Office of the Scottish Government Health and Social Care Directorates, Health and Social Care Research and Development Division (Welsh Government), Public Health Agency (Northern Ireland), British Heart Foundation, and Wellcome. The Fenland Study (10.22025/2017.10.101.00001) is funded by the Medical Research Council (MC_UU_12015/1). We are grateful to all the volunteers and to the general practitioners and practice staff for assistance with recruitment. We thank the Fenland Study Investigators; Fenland Study co-ordination team; and the Epidemiology Field, Data, and Laboratory teams. We further acknowledge support for genomics and metabolomics from the Medical Research Council (MC_PC_13046). TwinsUK is funded by the Wellcome Trust, Medical Research Council, European Union, the National Institute for Health Research (NIHR)-funded BioResource, Clinical Research Facility, and Biomedical Research Centre based at Guy's and St Thomas' NHS Foundation Trust in partnership with King's College London. This study makes use of the ICR1000 UK exome dataset generated by Professor Nazneen Rahman's team in the Division of Genetics & Epidemiology at The Institute of Cancer Research, London. The Genotype-Tissue Expression (GTEx) Project was supported by the Common Fund of the Office of the Director of the National Institutes of Health, and by NCI, NHGRI, NHLBI, NIDA, NIMH, and NINDS. The expression data described in this manuscript were obtained from the GTEx Portal on 26 February 2019. We thank Wellcome Sanger Institute DNA Pipelines team managers Richard Rance and Emma Gray, and Matt Mayho for technical support, as well as the staff of DNA Pipelines Operations, Sample Management, and Technical Administration for their contribution to this work. We would also like to thank members of the Sanger Institute Mouse Pipelines teams (Mouse Informatics, Molecular Technologies, Genome Engineering Technologies, Mouse Production Team, and Mouse Phenotyping) and the Research Support Facility for the provision and management of the mice. Microscopy was performed in the Wellcome-MRC IMS Image Core Facility and clinical studies were performed in the Wellcome-MRC IMS Translational Research Facility. I.B. and E.Z. acknowledge funding from Wellcome (WT206194), and I.S.F. was supported by Wellcome (207462/Z/17/Z), the NIHR Cambridge Biomedical Research Centre, an NIHR Senior Investigator Award, and the Bernard Wolfe Health Neuroscience Endowment. A.P. was supported by the Swiss National Science Foundation (P2EZP3_175090). Core Wellcome Sanger facilities are funded by Wellcome (WT098051 and WT206194), and the UK10K WES sequencing was funded by Wellcome (WT091310). The IMS Image Core and IMS Translational Research Facility are funded by Wellcome (208363/Z/17/Z).

AUTHOR CONTRIBUTIONS

I.B. and I.S.F. conceived the experiments and oversaw the study; G.M., A.E.H., and A.P. designed and performed most of the experiments and analyses; R.B., F.P., J.M.K., E.H., C.J.L., S.P., S.A., E.G.B., V.M., and A.D. contributed to participant recruitment and consenting, phenotyping, sequence analysis, or mouse experiments. N.J.W., S.O.R., C.L., C.H., and I.S.F. were responsible for contributing cohorts and samples. G.M., A.E.H., A.P., E.W., I.S.F., and I.B. drafted the manuscript. All authors critically reviewed, edited, and approved the final version of the manuscript.

DECLARATION OF INTERESTS

The authors declare no competing interests.

Received: December 17, 2019
Revised: March 6, 2020
Accepted: May 9, 2020
Published: June 2, 2020

REFERENCES

Abecasis, G.R., Altshuler, D., Auton, A., Brooks, L.D., Durbin, R.M., Gibbs, R.A., Hurles, M.E., and McVean, G.A.; 1000 Genomes Project Consortium (2010). A map of human genome variation from population-scale sequencing. Nature *467*, 1061–1073.

Akiyama, M., Okada, Y., Kanai, M., Takahashi, A., Momozawa, Y., Ikeda, M., Iwata, N., Ikegawa, S., Hirata, M., Matsuda, K., et al. (2017). Genome-wide association study identifies 112 new loci for body mass index in the Japanese population. Nat. Genet. *49*, 1458–1467.

Allison, D.B., Kaprio, J., Korkeila, M., Koskenvuo, M., Neale, M.C., and Hayakawa, K. (1996). The heritability of body mass index among an international sample of monozygotic twins reared apart. Int. J. Obes. Relat. Metab. Disord. *20*, 501–506.

Balduzzi, S., Rücker, G., and Schwarzer, G. (2019). How to perform a meta-analysis with R: a practical tutorial. Evid. Based Ment. Health *22*, 153–160.

Barroso, I., and McCarthy, M.I. (2019). The genetic basis of metabolic disease. Cell *177*, 146–161.

C Yuen, R.K., Merico, D., Bookman, M., L Howe, J., Thiruvahindrapuram, B., Patel, R.V., Whitney, J., Deflaux, N., Bingham, J., Wang, Z., et al. (2017). Whole genome sequencing resource identifies 18 new candidate genes for autism spectrum disorder. Nat. Neurosci. *20*, 602–611.

Clément, K., Biebermann, H., Farooqi, I.S., Van der Ploeg, L., Wolters, B., Poitou, C., Puder, L., Fiedorek, F., Gottesdiener, K., Kleinau, G., et al. (2018). MC4R agonism promotes durable weight loss in patients with leptin receptor deficiency. Nat. Med. *24*, 551–555.

Clifton, E.A., Day, F.R., De Lucia Rolfe, E., Forouhi, N.G., Brage, S., Griffin, S.J., Wareham, N.J., and Ong, K.K. (2017). Associations between body mass index-related genetic variants and adult body composition: the Fenland cohort study. Int. J. Obes. *41*, 613–619.

de Ligt, J., Willemsen, M.H., van Bon, B.W., Kleefstra, T., Yntema, H.G., Kroes, T., Vulto-van Silfhout, A.T., Koolen, D.A., de Vries, P., Gilissen, C., et al. (2012). Diagnostic exome sequencing in persons with severe intellectual disability. N. Engl. J. Med. *367*, 1921–1929.

DePristo, M.A., Banks, E., Poplin, R., Garimella, K.V., Maguire, J.R., Hartl, C., Philippakis, A.A., del Angel, G., Rivas, M.A., Hanna, M., et al. (2011). A framework for variation discovery and genotyping using next-generation DNA sequencing data. Nat. Genet. *43*, 491–498.

Di Angelantonio, E., Thompson, S.G., Kaptoge, S., Moore, C., Walker, M., Armitage, J., Ouwehand, W.H., Roberts, D.J., and Danesh, J.; INTERVAL Trial Group (2017). Efficiency and safety of varying the frequency of whole blood donation (INTERVAL): a randomised trial of 45 000 donors. Lancet *390*, 2360–2371.

Doche, M.E., Bochukova, E.G., Su, H.W., Pearce, L.R., Keogh, J.M., Henning, E., Cline, J.M., Saeed, S., Dale, A., Cheetham, T., et al. (2012). Human SH2B1 mutations are associated with maladaptive behaviors and obesity. J. Clin. Invest. *122*, 4732–4736.

Farhang-Fallah, J., Yin, X., Trentin, G., Cheng, A.M., and Rozakis-Adcock, M. (2000). Cloning and characterization of PHIP, a novel insulin receptor substrate-1 pleckstrin homology domain interacting protein. J. Biol. Chem. *275*, 40492–40497.

Farooqi, I.S., Jebb, S.A., Langmack, G., Lawrence, E., Cheetham, C.H., Prentice, A.M., Hughes, I.A., McCamish, M.A., and O'Rahilly, S. (1999). Effects of recombinant leptin therapy in a child with congenital leptin deficiency. N. Engl. J. Med. *341*, 879–884.

Gentleman, R.C., Carey, V.J., Bates, D.M., Bolstad, B., Dettling, M., Dudoit, S., Ellis, B., Gautier, L., Ge, Y., Gentry, J., et al. (2004). Bioconductor: open software development for computational biology and bioinformatics. Genome Biol. *5*, R80.

Grarup, N., Moltke, I., Andersen, M.K., Dalby, M., Vitting-Seerup, K., Kern, T., Mahendran, Y., Jørsboe, E., Larsen, C.V.L., Dahl-Petersen, I.K., et al. (2018). Loss-of-function variants in ADCY3 increase risk of obesity and type 2 diabetes. Nat. Genet. *50*, 172–174.

Hendricks, A.E., Bochukova, E.G., Marenne, G., Keogh, J.M., Atanassova, N., Bounds, R., Wheeler, E., Mistry, V., Henning, E., Körner, A., et al.; Understanding Society Scientific Group; EPIC-CVD Consortium; UK10K Consortium (2017). Rare variant analysis of human and rodent obesity genes in individuals with severe childhood obesity. Sci. Rep. *7*, 4394.

Hendricks, A.E., Billups, S.C., Pike, H.N.C., Farooqi, I.S., Zeggini, E., Santorico, S.A., Barroso, I., and Dupuis, J. (2018). ProxECAT: Proxy External Controls Association Test. A new case-control gene region association test using allele frequencies from public controls. PLoS Genet. *14*, e1007591.

Hodgkins, A., Farne, A., Perera, S., Grego, T., Parry-Smith, D.J., Skarnes, W.C., and Iyer, V. (2015). WGE: a CRISPR database for genome engineering. Bioinformatics *31*, 3078–3080.

Huang, J., Howie, B., McCarthy, S., Memari, Y., Walter, K., Min, J.L., Danecek, P., Malerba, G., Trabetti, E., Zheng, H.F., et al.; UK10K Consortium (2015). Improved imputation of low-frequency and rare variants using the UK10K haplotype reference panel. Nat. Commun. *6*, 8111.

Jang, S.M., Zhang, Y., Utani, K., Fu, H., Redon, C.E., Marks, A.B., Smith, O.K., Redmond, C.J., Baris, A.M., Tulchinsky, D.A., and Aladjem, M.I. (2018). The replication initiation determinant protein (RepID) modulates replication by recruiting CUL4 to chromatin. Nat. Commun. *9*, 2782.

Jansen, S., Hoischen, A., Coe, B.P., Carvill, G.L., Van Esch, H., Bosch, D.G.M., Andersen, U.A., Baker, C., Bauters, M., Bernier, R.A., et al. (2018). A genotype-first approach identifies an intellectual disability-overweight syndrome caused by PHIP haploinsufficiency. Eur. J. Hum. Genet. *26*, 54–63.

Jun, G., Flickinger, M., Hetrick, K.N., Romm, J.M., Doheny, K.F., Abecasis, G.R., Boehnke, M., and Kang, H.M. (2012). Detecting and estimating contamination of human DNA samples in sequencing and array-based genotype data. Am. J. Hum. Genet. *91*, 839–848.

Justice, A.E., Winkler, T.W., Feitosa, M.F., Graff, M., Fisher, V.A., Young, K., Barata, L., Deng, X., Czajkowski, J., Hadley, D., et al. (2017). Genome-wide meta-analysis of 241,258 adults accounting for smoking behaviour identifies novel loci for obesity traits. Nat. Commun. *8*, 14977.

Karp, N.A., Melvin, D., and Mott, R.F.; Sanger Mouse Genetics Project (2012). Robust and sensitive analysis of mouse knockout phenotypes. PLoS ONE *7*, e52410.

Karp, N.A., Speak, A.O., White, J.K., Adams, D.J., Hrabé de Angelis, M., Hérault, Y., and Mott, R.F. (2014). Impact of temporal variation on design and analysis of mouse knockout phenotyping studies. PLoS ONE *9*, e111239.

Katsanis, N. (2016). The continuum of causality in human genetic disorders. Genome Biol. *17*, 233.

Kojima, K.K., and Jurka, J. (2011). Crypton transposons: identification of new diverse families and ancient domestication events. Mob. DNA *2*, 12.

Kühnen, P., Clément, K., Wiegand, S., Blankenstein, O., Gottesdiener, K., Martini, L.L., Mai, K., Blume-Peytavi, U., Grüters, A., and Krude, H. (2016). Proopiomelanocortin deficiency treated with a melanocortin-4 receptor agonist. N. Engl. J. Med. *375*, 240–246.

Kurbatova, N., Mason, J.C., Morgan, H., Meehan, T.F., and Karp, N.A. (2015). PhenStat: a tool kit for standardized analysis of high throughput phenotypic data. PLoS ONE *10*, e0131274.

Lee, S., Emond, M.J., Bamshad, M.J., Barnes, K.C., Rieder, M.J., Nickerson, D.A., Christiani, D.C., Wurfel, M.M., and Lin, X.; NHLBI GO Exome Sequencing Project—ESP Lung Project Team (2012). Optimal unified approach for rare-variant association testing with application to small-sample case-control whole-exome sequencing studies. Am. J. Hum. Genet. *91*, 224–237.

Lee, S., Teslovich, T.M., Boehnke, M., and Lin, X. (2013). General framework for meta-analysis of rare variants in sequencing association studies. Am. J. Hum. Genet. *93*, 42–53.

Lee, D., Kim, E., and Tanaka-Yamamoto, K. (2016). Diacylglycerol kinases in the coordination of synaptic plasticity. Front. Cell Dev. Biol. *4*, 92.

Lek, M., Karczewski, K.J., Minikel, E.V., Samocha, K.E., Banks, E., Fennell, T., O'Donnell-Luria, A.H., Ware, J.S., Hill, A.J., Cummings, B.B., et al.; Exome Aggregation Consortium (2016). Analysis of protein-coding genetic variation in 60,706 humans. Nature *536*, 285–291.

Li, H. (2014). Toward better understanding of artifacts in variant calling from high-coverage samples. Bioinformatics *30*, 2843–2851.

Li, S., Francisco, A.B., Han, C., Pattabiraman, S., Foote, M.R., Giesy, S.L., Wang, C., Schimenti, J.C., Boisclair, Y.R., and Long, Q. (2010). The full-length isoform of the mouse pleckstrin homology domain-interacting protein (PHIP) is required for postnatal growth. FEBS Lett. *584*, 4121–4127.

Liu, X., Jian, X., and Boerwinkle, E. (2011). dbNSFP: a lightweight database of human nonsynonymous SNPs and their functional predictions. Hum. Mutat. *32*, 894–899.

Liu, X., Jian, X., and Boerwinkle, E. (2013). dbNSFP v2.0: a database of human non-synonymous SNVs and their functional predictions and annotations. Hum. Mutat. *34*, E2393–E2402.

Locke, A.E., Kahali, B., Berndt, S.I., Justice, A.E., Pers, T.H., Day, F.R., Powell, C., Vedantam, S., Buchkovich, M.L., Yang, J., et al.; LifeLines Cohort Study; ADIPOGen Consortium; AGEN-BMI Working Group; CARDIOGRAMplusC4D Consortium; CKDGen Consortium; GLGC; ICBP; MAGIC Investigators; MuTHER Consortium; MIGen Consortium; PAGE Consortium; ReproGen Consortium; GENIE Consortium; International Endogene Consortium (2015). Genetic studies of body mass index yield new insights for obesity biology. Nature *518*, 197–206.

Loveday, C., Tatton-Brown, K., Clarke, M., Westwood, I., Renwick, A., Ramsay, E., Nemeth, A., Campbell, J., Joss, S., Gardner, M., et al.; Childhood Overgrowth Collaboration (2015). Mutations in the PP2A regulatory subunit B family genes PPP2R5B, PPP2R5C and PPP2R5D cause human overgrowth. Hum. Mol. Genet. *24*, 4775–4779.

Marchini, J., and Howie, B. (2010). Genotype imputation for genome-wide association studies. Nat. Rev. Genet. *11*, 499–511.

Marouli, E., Graff, M., Medina-Gomez, C., Lo, K.S., Wood, A.R., Kjaer, T.R., Fine, R.S., Lu, Y., Schurmann, C., Highland, H.M., et al.; EPIC-InterAct Consortium; CHD Exome+ Consortium; ExomeBP Consortium; T2D-Genes Consortium; GoT2D Genes Consortium; Global Lipids Genetics Consortium; ReproGen Consortium; MAGIC Investigators (2017). Rare and low-frequency coding variants alter human adult height. Nature *542*, 186–190.

CellPress
OPEN ACCESS

Matsson, H., Tammimies, K., Zucchelli, M., Anthoni, H., Onkamo, P., Nopola-Hemmi, J., Lyytinen, H., Leppanen, P.H., Neuhoff, N., Warnke, A., et al. (2011). SNP variations in the 7q33 region containing DGKI are associated with dyslexia in the Finnish and German populations. Behav. Genet. *41*, 134–140.

McLaren, W., Gil, L., Hunt, S.E., Riat, H.S., Ritchie, G.R., Thormann, A., Flicek, P., and Cunningham, F. (2016). The Ensembl variant effect predictor. Genome Biol. *17*, 122.

Minster, R.L., Hawley, N.L., Su, C.T., Sun, G., Kershaw, E.E., Cheng, H., Buhule, O.D., Lin, J., Reupena, M.S., Viali, S., et al. (2016). A thrifty variant in CREBRF strongly influences body mass index in Samoans. Nat. Genet. *48*, 1049–1054.

Moore, C., Sambrook, J., Walker, M., Tolkien, Z., Kaptoge, S., Allen, D., Mehenny, S., Mant, J., Di Angelantonio, E., Thompson, S.G., et al. (2014). The INTERVAL trial to determine whether intervals between blood donations can be safely and acceptably decreased to optimise blood supply: study protocol for a randomised controlled trial. Trials *15*, 363.

O'Rahilly, S., and Farooqi, I.S. (2008). Human obesity: a heritable neurobehavioral disorder that is highly sensitive to environmental conditions. Diabetes *57*, 2905–2910.

Pigeyre, M., Yazdi, F.T., Kaur, Y., and Meyre, D. (2016). Recent progress in genetics, epigenetics and metagenomics unveils the pathophysiology of human obesity. Clin. Sci. (Lond.) *130*, 943–986.

Podcheko, A., Northcott, P., Bikopoulos, G., Lee, A., Bommareddi, S.R., Kushner, J.A., Farhang-Fallah, J., and Rozakis-Adcock, M. (2007). Identification of a WD40 repeat-containing isoform of PHIP as a novel regulator of beta-cell growth and survival. Mol. Cell. Biol. *27*, 6484–6496.

Price, A.L., Patterson, N.J., Plenge, R.M., Weinblatt, M.E., Shadick, N.A., and Reich, D. (2006). Principal components analysis corrects for stratification in genome-wide association studies. Nat. Genet. *38*, 904–909.

Purcell, S., Neale, B., Todd-Brown, K., Thomas, L., Ferreira, M.A., Bender, D., Maller, J., Sklar, P., de Bakker, P.I., Daly, M.J., and Sham, P.C. (2007). PLINK: a tool set for whole-genome association and population-based linkage analyses. Am. J. Hum. Genet. *81*, 559–575.

Purcell, S.M., Moran, J.L., Fromer, M., Ruderfer, D., Solovieff, N., Roussos, P., O'Dushlaine, C., Chambert, K., Bergen, S.E., Kähler, A., et al. (2014). A polygenic burden of rare disruptive mutations in schizophrenia. Nature *506*, 185–190.

Saeed, S., Bonnefond, A., Tamanini, F., Mirza, M.U., Manzoor, J., Janjua, Q.M., Din, S.M., Gaitan, J., Milochau, A., Durand, E., et al. (2018). Loss-of-function mutations in ADCY3 cause monogenic severe obesity. Nat. Genet. *50*, 175–179.

Samocha, K.E., Robinson, E.B., Sanders, S.J., Stevens, C., Sabo, A., McGrath, L.M., Kosmicki, J.A., Rehnström, K., Mallick, S., Kirby, A., et al. (2014). A framework for the interpretation of de novo mutation in human disease. Nat. Genet. *46*, 944–950.

Singh, T., Kurki, M.I., Curtis, D., Purcell, S.M., Crooks, L., McRae, J., Suvisaari, J., Chheda, H., Blackwood, D., Breen, G., et al.; Swedish Schizophrenia Study; INTERVAL Study; DDD Study; UK10 K Consortium (2016). Rare loss-of-function variants in SETD1A are associated with schizophrenia and developmental disorders. Nat. Neurosci. *19*, 571–577.

Singh, T., Walters, J.T.R., Johnstone, M., Curtis, D., Suvisaari, J., Torniainen, M., Rees, E., Iyegbe, C., Blackwood, D., McIntosh, A.M., et al.; INTERVAL Study; UK10K Consortium (2017). The contribution of rare variants to risk of schizophrenia in individuals with and without intellectual disability. Nat. Genet. *49*, 1167–1173.

Styne, D.M., Arslanian, S.A., Connor, E.L., Farooqi, I.S., Murad, M.H., Silverstein, J.H., and Yanovski, J.A. (2017). Pediatric obesity-assessment, treatment, and prevention: an endocrine society clinical practice guideline. J. Clin. Endocrinol. Metab. *102*, 709–757.

Turcot, V., Lu, Y., Highland, H.M., Schurmann, C., Justice, A.E., Fine, R.S., Bradfield, J.P., Esko, T., Giri, A., Graff, M., et al.; CHD Exome+ Consortium; EPIC-CVD Consortium; ExomeBP Consortium; Global Lipids Genetic Consortium; GoT2D Genes Consortium; EPIC InterAct Consortium; INTERVAL Study; ReproGen Consortium; T2D-Genes Consortium; MAGIC Investigators; Understanding Society Scientific Group (2018). Protein-altering variants associated with body mass index implicate pathways that control energy intake and expenditure in obesity. Nat. Genet. *50*, 26–41.

Van der Auwera, G.A., Carneiro, M.O., Hartl, C., Poplin, R., Del Angel, G., Levy-Moonshine, A., Jordan, T., Shakir, K., Roazen, D., Thibault, J., et al. (2013). From FastQ data to high confidence variant calls: the Genome Analysis Toolkit best practices pipeline. Curr. Protoc. Bioinformatics *43*, 1–33.

van der Klaauw, A.A., and Farooqi, I.S. (2015). The hunger genes: pathways to obesity. Cell *161*, 119–132.

Walter, K., Min, J.L., Huang, J., Crooks, L., Memari, Y., McCarthy, S., Perry, J.R., Xu, C., Futema, M., Lawson, D., et al.; UK10K Consortium (2015). The UK10K project identifies rare variants in health and disease. Nature *526*, 82–90.

Wang, C., Zhan, X., Liang, L., Abecasis, G.R., and Lin, X. (2015). Improved ancestry estimation for both genotyping and sequencing data using projection procrustes analysis and genotype imputation. Am. J. Hum. Genet. *96*, 926–937.

Webster, E., Cho, M.T., Alexander, N., Desai, S., Naidu, S., Bekheirnia, M.R., Lewis, A., Retterer, K., Juusola, J., and Chung, W.K. (2016). De novo *PHIP*-predicted deleterious variants are associated with developmental delay, intellectual disability, obesity, and dysmorphic features. Cold Spring Harb. Mol. Case Stud. *2*, a001172.

Wheeler, E., Huang, N., Bochukova, E.G., Keogh, J.M., Lindsay, S., Garg, S., Henning, E., Blackburn, H., Loos, R.J., Wareham, N.J., et al. (2013). Genome-wide SNP and CNV analysis identifies common and low-frequency variants associated with severe early-onset obesity. Nat. Genet. *45*, 513–517.

White, J.K., Gerdin, A.K., Karp, N.A., Ryder, E., Buljan, M., Bussell, J.N., Salisbury, J., Clare, S., Ingham, N.J., Podrini, C., et al.; Sanger Institute Mouse Genetics Project (2013). Genome-wide generation and systematic phenotyping of knockout mice reveals new roles for many genes. Cell *154*, 452–464.

Yang, J., Seo, J., Nair, R., Han, S., Jang, S., Kim, K., Han, K., Paik, S.K., Choi, J., Lee, S., et al. (2011). DGKι regulates presynaptic release during mGluR-dependent LTD. EMBO J. *30*, 165–180.

Yuen, R.K., Thiruvahindrapuram, B., Merico, D., Walker, S., Tammimies, K., Hoang, N., Chrysler, C., Nalpathamkalam, T., Pellecchia, G., Liu, Y., et al. (2015). Whole-genome sequencing of quartet families with autism spectrum disorder. Nat. Med. *21*, 185–191.

Zuk, O., Schaffner, S.F., Samocha, K., Do, R., Hechter, E., Kathiresan, S., Daly, M.J., Neale, B.M., Sunyaev, S.R., and Lander, E.S. (2014). Searching for missing heritability: designing rare variant association studies. Proc. Natl. Acad. Sci. USA *111*, E455–E464.

STAR★METHODS

KEY RESOURCES TABLE

REAGENT or RESOURCE	SOURCE	IDENTIFIER
**Antibodies**		
Rabbit anti-PHIP	Proteintech	Cat # 20933-1-AP; RRID: AB_10733522
Goat anti rabbit secondary antibody Alexa Fluor 488	Thermo Fisher Scientific	Cat# A11034; RRID: AB_2576217
Mouse anti-HA tag (6E2)	Cell Signaling	Cat# 2367; RRID: AB_10691311
Normal Rabbit IgG	Cell Signaling	Cat# 2729; RRID: AB_1031062
Rabbit anti-IRS2 (L1326)	Cell Signaling	Cat# 3089; RRID: AB_2125771
Rabbit anti-PHIP	Abcam	Cat# ab86244; RRID: AB_1925318
Goat anti-rabbit IgG-HRP	Dako	Cat# P0448; RRID: AB_2617138
Rabbit anti-p44/42 MAPK (Erk1/2) (137F5)	Cell Signaling Technology	Cat# 4695; RRID: AB_390779
Rabbit anti-Phospho-p44/42 MAPK (Erk1/2) (Thr202/Tyr204)	Cell Signaling Technology	Cat# 9101; RRID: AB_331646
Rabbit anti-STAT3	Cell Signaling Technology	Cat# 4904; RRID: AB_331269
Rabbit anti-Phospho STAT3 (pY705)	Cell Signaling Technology	Cat# 9131; RRID: AB_331586
Rabbit anti-βActin	Cell Signaling Technology	Cat# 4967; RRID: AB_330288
**Bacterial and Virus Strains**		
XL10-Gold	Agilent	Cat# 200315
**Chemicals, Peptides, and Recombinant Proteins**		
Lipofectamine 2000	GIBCO	Cat#11668
Formaldehyde	Fisher Chemicals	F/150/PB17
Triton X-100	BDH	306324N
Human Recombinant E.coli Leptin	EMD Millipore	429700
Protein A Sepharose	Abcam	ab193256
DAPI	Invitrogen	D1306
**Critical Commercial Assays**		
Steadylite Plus Reporter Gene Assay System	Perkin Elmer	6066759
Sample-to-SNP kit	ThermoFisher	4403081
**Experimental Models: Cell Lines**		
HEK293	ATCC	CRL-1573; RRID: CVCL_0045
COS7	culture collection by Alan Tunnacliffe	N/A
**Experimental Models: Organisms/Strains**		
Dgki (EM:11471), Zmym4 (EM:11435) and Znf32/Zfp637 (EM:11616)	Infrafrontier (https://www.infrafrontier.eu/) or they can be obtained from WSI directly: mouseinterest@sanger.ac.uk	EM:11471, EM:11435, EM:11616
**Recombinant DNA**		
Human N-HA-PHIP-WT in pCDNA3.1(+) vector	This paper	N/A
Human N-HA-PHIP variants in pCDNA3.1(+) vector	This paper	N/A
**Software and Algorithms**		
Prism 7	Graph Pad Software	https://www.graphpad.com/scientific-software/prism/
WGE CRISPR tool	(Hodgkins et al., 2015)	N/A

(Continued on next page)

Continued

REAGENT or RESOURCE	SOURCE	IDENTIFIER
GATK Haplotype Caller (v3.2)	(DePristo et al., 2011; Van der Auwera et al., 2013)	https://github.com/broadinstitute/gatk/releases
VerifyBamID (v1.0)	(Jun et al., 2012)	https://genome.sph.umich.edu/wiki/VerifyBamID
EIGENSTRAT v4.2	(Price et al., 2006)	https://www.hsph.harvard.edu/alkes-price/software/
Meta, R package v4.9	(Balduzzi et al., 2019)	N/A
PhenStat, R package version 2.18.0	(Kurbatova et al., 2015)	Available from Bioconductor (Gentleman et al., 2004)
PLINK	(Purcell et al., 2007)	http://zzz.bwh.harvard.edu/plink/
PLINK/SEQ	N/A	https://atgu.mgh.harvard.edu/plinkseq/index.shtml
ProxECAT	(Hendricks et al., 2018)	N/A
SNPtest v2.5	(Marchini and Howie, 2010)	https://mathgen.stats.ox.ac.uk/genetics_software/snptest/snptest.html
Variant Effect Predictor (VEP) version 79 with the dbNSFP plug-in (dbNSFPv2.9, Feb 3, 2015	(McLaren et al., 2016; Liu et al., 2011, 2013)	http://www.ensembl.org/vep; https://sites.google.com/site/jpopgen/dbNSFP
SKAT, R package version 1.1	(Lee et al., 2012)	Available from CRAN (https://cran.r-project.org/web/packages/SKAT/index.html)
MetaSKAT, R package version 0.60	(Lee et al., 2013)	Available from CRAN (https://cran.r-project.org/web/packages/MetaSKAT/index.html)

RESOURCE AVAILABILITY

Lead Contact

Further information and requests for reagents may be directed to and will be fulfilled by the Lead Contact, Inês Barroso (ines.barroso@exeter.ac.uk).

Materials Availability

Plasmids generated in this study are available from the lead contact. Mouse lines generated in this study will be available from Infrafrontier (https://www.infrafrontier.eu/) or they can be obtained from WSI directly, mouseinterest@sanger.ac.uk. Dgki (EM:11471), Zmym4 (EM:11435) and Znf32/Zfp637 (EM:11616). There are currently some restrictions to the availability of mouse lines from Infrafrontier due to the patent issues surrounding CRISPR-generated mice, however these restrictions are being resolved and in the interim mouse lines are available directly from Wellcome Sanger Institute by contacting mouseinterest@sanger.ac.uk.

Data and Code Availability

SCOOP and INTERVAL WES data are accessible from the European Genome-phenome Archive- EGA: EGAS00001000124 and EGA: EGAS00001000825, respectively. Adult obesity WES data from UK10K Generation Scotland and TwinsUK are available from EGA under accession codes EGA: EGAS00001000242 and EGA: EGAS00001000306, respectively. 1958 Birth Cohort WES data is available from the EGA under accession code EGA: EGAS00001000971. All other data are available in the manuscript or the supplementary materials.

EXPERIMENTAL MODEL AND SUBJECT DETAILS

Human Studies

All studies were approved by the Cambridge Local Research Ethics Committees and all participants and their parents (for children below the age of 16) gave written informed consent. All research was conducted in line with the principles outlined in the Declaration of Helsinki.

Samples

Stage 1 used whole-exome sequence data from participants in the SCOOP and INTERVAL studies (details below). Stage 2 included targeted sequence and genotype data obtained for additional SCOOP participants and participants from the FENLAND study (details

below). Whole-exome sequence adult obesity data was obtained for adult obesity participants in the UK10K project (Walter et al., 2015) and 1958 Birth Cohort participants were used as controls, but only data for four genes surviving combined stage1+stage2 analysis were analyzed.

Childhood Obesity Cases

The Severe Childhood Onset Obesity Project (SCOOP) cohort (Wheeler et al., 2013), includes ~4800 British individuals of European ancestry with childhood onset obesity (BMI standard deviation score (SDS) > 3; onset of obesity before the age of 10 years). SCOOP individuals likely to have congenital leptin deficiency, a treatable cause of severe obesity, were excluded by measurement of serum leptin, and individuals with mutations in the melanocortin 4 receptor gene (*MC4R*) (the most common genetic form of penetrant obesity) were excluded by prior Sanger sequencing. All participants had age < 10y at the time of recruitment, sex distribution was: Female 548 (59.12%), Male 379 (40.88%).

In this study, SCOOP participants were included in stage 1 and stage 2 analyses. Stage 1 analysis comprised 982 SCOOP individuals with whole-exome sequence (WES) data obtained as part of the UK10K consortium project (Walter et al., 2015; Hendricks et al., 2017). WES sequence data can be obtained from the European Genome-phenome Archive (EGA) under study accession code EGAS00001000124 (Walter et al., 2015; Hendricks et al., 2017). Stage 2 analyses included 1,816 SCOOP participants selected from a total of 2,819 participants with existing sequence data on ~1,300 genes (Walter et al., 2015). Selection of stage 2 samples was based on: i) presence of good quality sequence data (proxy for good quality DNA); ii) European ancestry as defined by principal component analysis on off-target variants (LASER 2.0 algorithm (Wang et al., 2015) and; iii) unrelated to SCOOP stage 1 samples. All participants had age < 10y at the time of recruitment, sex distribution was: Female 951 (52.37%), Male 865 (47.63%).

Adult Obesity Cases

WES data obtained as part of the UK10K consortium project (Walter et al., 2015) was available for 366 Generation Scotland and 65 TwinsUK unrelated participants with BMI > 40, and good quality sequence data. These data are available from EGA under accession codes EGA: EGAS00001000242 and EGA: EGAS00001000306. All TwinsUK participants were female, but age information was not available to us at the time of analysis. Age and sex of GS samples were not available to us at the time of analysis. The effect of age and sex was not considered in this study as our discovery study design did not allow us to investigate these parameters due to both power issues (low power for stratified analysis), and the fact that the discovery phase contrasted obese prepubertal children with age < 10y at recruitment with control adults.

Population Controls

Stage 1 analysis included 4,502 participants from the INTERVAL cohort. The INTERVAL cohort consists of 50,000 predominantly healthy blood donors in the UK (Moore et al., 2014). All individuals were genotyped using the UK Biobank Axiom Array (Affymetrix Axiom Biobank Array) and imputed using a combined UK10K-1000G Phase 3 imputation panel (Huang et al., 2015). A subset of 4,502 individuals were selected for whole-exome sequencing, as previously described (Singh et al., 2016), of which 4,499 survived QC and were used as controls in this study. Information on age and sex was available to us for 4,045 of the 4,057 participants (99.70%): Age mean (SD): 43.51 (14.31); Sex Female 1,994 (49.30%), Male 2,051 (50.70%). Further details on the INTERVAL study can be obtained at https://www.intervalstudy.org.uk/.

Stage 2 analysis included participants from Phase 1 of the Fenland Study. The Fenland Study is a population-based cohort of 12,435 participants born between 1950 and 1975, recruited from participating General Practices from around the Cambridgeshire region in the UK. Exclusion criteria were: clinically diagnosed diabetes mellitus, inability to walk unaided, terminal illness (life expectancy of ≱1 year at the time of recruitment), clinically diagnosed psychotic disorder, pregnancy or lactation (Clifton et al., 2017). Participants were aged 29-64 years and 53.8% were female. For the stage 2 genotyping (see below) we used 3,800 randomly selected Fenland Study participants (age range 29-64, of which sex information was available to us for 3,777 that passed QC: Female 2,040 (54.01%), Male 1,737 (45.99%)), of which a subset of 2,660 (age range 29-64, of which sex was available to us for 2,627: Female 1,392 (52.99%), Male 1,235 (47.01%)) was randomly selected for the stage 2 targeted gene sequencing (see below). Further details on the Fenland Study, including a technical summary, can be found here: http://www.mrc-epid.cam.ac.uk/research/studies/fenland/

Association analysis with adult obesity used 1,000 participants from the 1958 Birth Cohort as controls. The 1958 Birth Cohort Collection is a population-based collection of all individuals born in a week in 1958 in the UK (http://www.cls.ioe.ac.uk). Whole-exome data were obtained from EGA under accession code EGA: EGAS00001000971. Age and sex information of participants included in this study was not available at the time of analysis and was not considered in this study as our discovery study design did not allow us to investigate these parameters due to both power issues (low power for stratified analysis), and the fact that the discovery phase contrasted obese prepubertal children with age < 10y at recruitment with control adults. Briefly, genomic DNA was used to prepare DNA libraries using the Illumina TruSeq sample preparation kit. DNA was fragmented using Covaris technology and libraries were prepared without gel size selection. Target enrichment was performed in pools of six libraries using the Illumina TruSeq Exome Enrichment kit. Captured DNA libraries were PCR amplified using the supplied paired-end PCR primers. Sequencing was performed with an Illumina HiSeq2000 (v3 flow cell, one pool per lane) generating 2x100-bp reads (Loveday et al., 2015).

gnomAD controls were the non-Finnish control exome samples (N = 21,384). GnomAD v2.1 data was downloaded on October 12, 2018 from https://gnomad.broadinstitute.org/downloads

Mice

Animals, Housing and Husbandry

All mice were maintained in specific pathogen-free facilities in individually ventilated cages at standard temperature (19-23°C) and humidity (55% ± 10%), on a 12 h dark, 12 h light cycle (lights on 0730–190) and fed a breeder's chow diet (LabDiet 5021-3, 9% crude fat content, 21% kcal as fat, 0.276ppm cholesterol, LabDiet, London, UK).

All mice were given water and diet *ad libitum*, unless otherwise stated. Mice were maintained in a specific pathogen free unit on a 12 h light: 12 h dark cycle with lights off at 7:30pm and no twilight period. The ambient temperature was 21 ± 2°C and the humidity was 55 ± 10%. Mice were typically housed for phenotyping using a stocking density of 3-5 mice per cage (overall dimensions of caging: (L x W x H) 365 × 207 × 140mm, floor area 530cm^2) in individually ventilated caging (Tecniplast Seal Safe Plus GM500) receiving 60 air changes per h. In addition to Aspen bedding substrate, standard environmental enrichment of two nestlets, a cardboard tunnel and three wooden chew blocks was provided. Mice stocking density was typically 3-5 mice per cage. The care and use of mice was performed in accordance with UK Home Office regulations, UK Animals (Scientific Procedures) Act of 1986 under a UK Home Office license (P77453634) and which were reviewed regularly by the WTSI Animal Welfare and Ethical Review Body. Animal welfare was assessed routinely for all mice involved. Mouse lines for the genes used in this study [Dgki (EM:11471), Zmym4 (EM:11435) and Znf32/Zfp637 (EM:11616)] can be ordered from Infrafrontier (https://www.infrafrontier.eu/).

Generation of Dgki$^{em1(IMPC)Wtsi}$ Mutant Mice

C57BL/6N mouse zygotes were injected cytoplasmically with Cas9 mRNA (50ng/μl) from Trilink Biotechnologies and two pairs of *in vitro* transcribed gRNAs (6.25ng/μl each) flanking a critical exon. gRNAs were identified using the WGE CRISPR tool (Hodgkins et al., 2015) and were selected based on their off-target scores to minimize potential off target damage.

Sequence	Chromosome	Chromosome Start	Chromosome End
GCACTGATCCAACAATTTGGTGG	6	37049961	37049983
ATATTATGGCCATATTACGGAGG	6	37050131	37050153
CCTGTAGACTGTCCCAAATCCAT	6	37050402	37050424
CCTGAGTAGTTCCATTAGACTTA	6	37050669	37050691

The zygotes were transferred into the oviduct of pseudopregnant females the same day of microinjection. G0 founder mosaic offspring were identified using a combination of end point PCR and gene-specific 'loss of WT allele' (LoA) qPCR assay designed to the region of the genome predicted to be deleted. G0 founder mice were mated to C57BL/6N mice to establish G1's for further breeding to C57BL/6N mice. Genomic DNA from pups produced by cytoplasmic injection of CRISPR/Cas9 reagents was isolated from an ear punch of two week old pups using the Sample-to-SNP kit (ThermoFisher, 4403081).

Endpoint PCR Primer Pairs and Expected Size Bands

Assay type	Assay	Forward Primer	Reverse Primer	Expected Size Band (bp)
Standard PCR	Wild type	Dgki_DF1	Dgki_ER1	363
Standard PCR	Wild type	Dgki_EF1	Dgki_DR1	566
Standard PCR	Mutant	Dgki_DF1	Dgki_DR1	286

Primer Name	Primer Sequence (5′ > 3′)
Dgki_DF1	GTCTCCAAAATCAGACACGCA
Dgki_EF1	ACAAAAGGCATTTTTCCCACC
Dgki_ER1	GGTACCTGAATCCACGGCAA
Dgki_DR1	ATGACATAGCCTGGCCACTT

LoA qPCR Primers

Target	Forward Primer Seq.	Reverse Primer Seq.	Probe Primer Seq.
Dgki	AGATCAAAGACTTGCCGTGGAT	CCTTTATAGGGAACCAAAGTCCTACA	CAGGTACCACATAAAC

Viability of the line is assessed by genotyping a minimum of 28 offspring from heterozygous intercrosses. For *Dgki*$^{em1(IMPC)Wtsi}$, this gave 23 homozygous mice from 95 offspring (Expected: 24 from 95 offspring, Fisher- exact test, p = 1.0). Mice were allocated to the pipeline randomly by Mendelian Inheritance.

Generation of Zmym4$^{em1(IMPC)Wtsi}$ Mutant Mice

C57BL/6N mouse zygotes were injected cytoplasmically with Cas9 mRNA (50ng/µl) from Trilink Biotechnologies and *in vitro* transcribed gRNAs (6.25ng/µl each) flanking a critical exon. gRNAs were identified using the WGE CRISPR tool (Hodgkins et al., 2015) and were selected based on their off-target scores to minimize potential off target damage.

Sequence	Chromosome	Chromosome Start	Chromosome End
CCACTATTCGGCTAAAAGATGCA	4	126910579	126910601
CGTAATGCATGTACAGAAACTGG	4	126910605	126910627
CCACCCTCTTGGTATATTAAAGG	4	126911331	126911353

The zygotes were transferred into the oviduct of pseudopregnant females the same day of microinjection. G0 founder mosaic offspring were identified using a combination of end point PCR and gene-specific 'loss of WT allele' (LoA) qPCR assay designed to the region of the genome predicted to be deleted. G0 founder mice were mated to C57BL/6N mice to establish G1's for further breeding to C57BL/6NTac mice. Genomic DNA from pups produced by cytoplasmic injection of CRISPR/Cas9 reagents was isolated from an ear punch of two week old pups using the Sample-to-SNP kit (ThermoFisher, 4403081).

Endpoint PCR Primer Pairs and Expected Size Bands

Assay type	Assay	Forward Primer	Reverse Primer	Expected Size Band (bp)
Standard PCR	Wild type	Zmym4_DF1	Zmym4_ER1	274
Standard PCR	Wild type	Zmym4_EF1	Zmym4_DR1	531
Standard PCR	Mutant	Zmym4_DF1	Zmym4_DR1	98

Primer Name	Primer Sequence (5′ > 3′)
Zmym4_DF1	CCACCACCCAGCCTAAAAGA
Zmym4_EF1	CCCCCACAGTTCTCACAACA
Zmym4_ER1	TCAGGGGAGTTGAAACCTTGG
Zmym4_DR1	GGTGCTCTTACCCACTGAGC

LoA qPCR Primers

Target	Forward Primer Seq.	Reverse Primer Seq.	Probe Primer Seq.
Zmym4	TGAGGTGACACACATTGAACTACAA	CAGTGCCATGTGCTGCAAAT	ACTTCTTAGACTGCCCCTC

Viability of the line is assessed by genotyping a minimum of 28 offspring from heterozygous intercrosses. For *Zmym4*$^{em1(IMPC)Wtsi}$ this gave 0 homozygous mice from 56 offspring (Expected: 14 from 56 offspring, Fisher- exact test, $p < 0.0001$).

Primary Standardized Phenotyping Pipeline

Mice underwent primary standardized phenotyping from 4 weeks of age and had only previously undergone earclip biopsies to identify the mice and establish genotype before entering the pipeline. Mice were randomly assigned to cohorts by Mendelian inheritance by colony managers that were separate from the team performing the phenotyping. *Dgki* homozygous mutant mice on the C57BL/6NTac background were tested in 7 batches (2 batches of 2 females each, 1 batch of 1 male, 1 batch of 1 female, 1 batch of 2 females and 1 male, 1 batch of 2 males and 1 batch of 3 males). All 14 *Dgki* mutant mice completed the pipeline without loss due to welfare or health concerns. No health concerns or adverse effects were noted during or outside of procedures. With each batch of mutant mice, a cohort of typically 7 age and sex matched (though not littermate) wild-type C57BL/6NTac mice were also phenotyped, to provide longitudinal control values as described in the Quantification and Statistical Analysis section. Key phenotyping results are shown in Figures S6 and S7. Data collected include weight curves, basic behavioral/morphological assessment at 9 weeks of age, intraperitoneal glucose tolerance test (ipGTT) at 13 weeks and body composition assessment by dual emission X-ray absorptiometry (DEXA) at 14 weeks of age using a modified version of the MGP pipeline detailed previously (White et al., 2013), using a mouse breeder's chow (LabDiets 5021, 9% crude fat content, 21% kcal as fat, 0.276ppm cholesterol, LabDiet, London, UK) instead of a high fat diet. At 16 weeks, random-fed mice were anesthetized using 100 mg/kg Ketamine and 10 mg/kg Xylazine and blood was collected retroorbitally. Samples were used to measure total blood counts, clinical chemistry parameters and for analysis by flow cytometry. Death was confirmed by cervical dislocation and heart removal. As a high throughput screen where genes are selected for study without hypothesis and mice are studied in multiple batches and alongside mice with mutations for different genes, there is limited

room for personal bias to influence the results. For mouse management purposes, the cages have both genotype and allele information and hence the intraperitoneal glucose tolerance test (ipGTT) and Dual-energy X-ray absorptiometry (DEXA) screens are run unblinded. Basic behavioral/morphological assessments were run with the technician blinded to the specific allele of the mouse, but not whether the mouse was wild type or a mutant. Necropsies of mice were performed by staff blinded to genotype and allele. Analysis of blood samples for clinical chemistry were run blind using a barcode system. Typically, necropsies and blood collections are performed from 9am-12pm, fasting for GTT starts 9am with the sample collection beginning at 1pm. DEXA and behavioral analysis could be performed at any time between 9am and 4pm. The order of cages and mice treated and assessed in any given procedure was not predetermined. All data was collected using a bespoke mouse and data management system to allow QC of data under predefined conditions, and data was only analyzed once all data from that line had been collected. The standard operating procedures can be found at IMPReSS (https://www.mousephenotype.org/impress).

Dual-energy X-ray absorptiometry: DEXA was performed using an Ultrafocus 100 (Faxitron Bioptics LLC, Tuscon, Arizona, USA) under isofluorane anesthesia (IsoFlo, Zoetis UK Ltd., London, UK), in order to minimize recovery times while immobilizing the mouse for data collection. Nose to tail base length measurements were performed using a ruler with 1mm graduations prior to DEXA measurement. Parameters measured included fat mass (g), fat percentage estimate (%), lean mass (g), bone mineral density (mg/cm^2), and bone mineral content (g). Internal calibration was performed prior to any imaging.

Intraperitoneal Glucose tolerance test: Mice were single-housed and fasted for 4 h. Approximately 0.5mm of the tail tip was removed with a scalpel blade and a fasting blood sample (T0) was directly taken (Accu-chek Aviva, Roche, Indianapolis, IN). Mice were then injected with 2g/kg glucose intraperitoneally and further blood samples were taken at 15 (T15), 30 (T30), 60 (T60) and 120 (T120) minutes post-glucose injection. Area under the curve (AUC) was calculated using the trapezoid method, where glucose at T0 was used as the baseline value for the mouse. Two female and one male homozygous mice had their T15 to T120 data removed from analysis due to glucose injection failures.

Clinical Chemistry (CC): Blood was collected from animals in the random-fed state between 08:30 and 10:30. Mice were anesthetized using 100 mg/kg Ketamine and 10 mg/kg Xylazine and blood was collected into heparinized pediatric tubes (Kabe Labortechnik GmbH, Numbrecht, Germany) using the retro-orbital route, followed by heart removal and cervical dislocation. This anesthesia/administration combination allowed the collection of a sufficient amount of non-hemolyzed blood, particularly for electrolyte parameters that may be strongly affected by hemolysis. Heparinized whole-blood samples were centrifuged at 5,000 rcf for 10 min at 4°C, and the plasma was collected and stored at 4°C until analysis, always within 1 h of collection. Plasma variables were assessed at room temperature using an AU480 chemistry analyzer (Beckman Coulter, High Wycombe, UK). Glucose levels were not analyzed from the clinical chemistry screen due to the rapid increase in plasma glucose under Ketamine/Xylazine based anesthesia.

METHOD DETAILS

Cell Culture

HEK293 (XX female) cells were cultured in high glucose Dulbecco's modified eagle medium (DMEM, GIBCO, 41965) supplemented with 10% fetal bovine serum (GIBCO, 10270, South America origin), 1% GlutaMAX (100X) (GIBCO, 35050), and 100 units/mL penicillin and 100 μg/mL streptomycin (Sigma-Aldrich, P0781) at 37°C, 5% CO2. COS-7 (XY male) cells were cultured in low glucose Dulbecco's modified Eagle's medium (Sigma, D6046) supplemented with 10% fetal bovine serum, 1% GlutaMAX, 100 IU/mL penicillin and 100 ng/mL streptomycin at 37°C, 5% CO2.

Cloning of PHIP Human Variants

PHIP cDNA constructs containing an N-terminal HA tag in pCDNA3.1 (+) vector (Invitrogen) were used throughout the study (NM_017934.7). Site-directed mutagenesis was performed using Q5 site-directed mutagenesis kit (NEB, E0554S) according to the manufacturer's protocols. All constructs were verified with Sanger sequencing.

Subcellular Localization of Endogenous PHIP

COS-7 cells were seeded in black clear bottom CellCarrier-96 Ultra Microplates (Perkin Elmer, 6055302) coated with Poly-D-Lysine solution (Sigma, A-003-E) (20.000 cells/well). After 24 h, cells were fixed with 4% Formaldehyde (Fisher Chemicals, F/150/PB17) in Phosphate-buffered saline (PBS) for 20 min at room temperature, permeabilized with 0.2% Triton X-100 (BDH, 306324N) for 30 min at room temperature, blocked for 1 h in 3% Bovine Serum Albumine (BSA) (Sigma, A7906) at room temperature, and incubated overnight at 4°C with Rabbit anti-PHIP (Proteintech, 20933-1-AP) in 1:100 dilution in 3% BSA or without antibody as a negative control. Cells were washed three times with PBS for 5 min, incubated with goat anti rabbit secondary antibody Alexa Fluor 488 (Thermo Fisher Scientific, A11034) in 1:200 dilution in 3% BSA for 1 h at room temperature, washed 2 times with PBS for 5 min, incubated with DAPI (Invitrogen, D1306) in 1:500 dilution in PBS for 10 min and kept in PBS. Cells were imaged in the Opera Phenix High Content Screening Confocal system (Perkin Elmer).

Luciferase POMC Transcription Activation Assay

HEK293 cells were seeded into white 96-well plates coated with Poly-D-Lysine (40,000 cells/well) and transiently transfected the next day with 100ng/well plasmid encoding either empty pcDNA3.1(+) vector (negative control), WT or mutant PHIP plasmid, combined with 50ng/well plasmid for Leptin Receptor, 50ng/well plasmid for POMC luciferase and 10ng/well plasmid for STAT3 using

Lipofectamine 2000 (Thermo Fisher Scientific, 11668019) in serum-free Opti-MEM I medium (GIBCO, 31985) according to the manufacturer's protocols. After 6 h transfection media was replaced by full DMEM. The next day cells were incubated overnight with starvation media (DMEM no FBS) with or without the presence of 200ng/mL Leptin (Human Recombinant E.coli, EMD Millipore, 429700). Quantitation of firefly luciferase activity was performed using the Steadylite Plus Reporter Gene Assay System (Perkin Elmer, 6066759) according to the manufacturer's protocol.

Dominant Negative Luciferase POMC Transcription Activation Assay

HEK293 cells were seeded in white 96-well plates coated with Poly-D-Lysine (40,000 cells/well) and transiently transfected the next day with 50ng/well plasmid encoding empty pcDNA3.1(+) vector together with 50ng/well WT PHIP plasmid or 50ng/well WT PHIP plasmid together with different concentrations of mutant PHIP plasmid and combined with 50ng/well plasmid for Leptin Receptor, 50ng/well plasmid for POMC luciferase and 10ng/well plasmid for STAT3 using Lipofectamine 2000 in Opti-MEM I medium according to the manufacturer's protocols. After 6 h, transfection media was replaced by full DMEM. The next day cells were incubated overnight with starvation media (DMEM no FBS) with or without the presence of 200ng/mL Leptin. Quantitation of firefly luciferase activity was performed using the Steadylite Plus Reporter Gene Assay System (Perkin Elmer, 6066759) according to the manufacturer's protocol.

Subcellular Localization of Human PHIP Variants

COS-7 cells were seeded into black clear bottom CellCarrier-96 Ultra Microplates coated with Poly-D-Lysine (20.000 cells/well) or into glass coverslips in 12-well plates coated with Poly-D-Lysine (150.000 cells/well). Cells were transiently transfected with 100ng/well plasmid encoding either empty pcDNA3.1(+) vector (negative control), WT or mutant PHIP plasmid, combined with 50ng/well plasmid for Leptin Receptor, and 10ng/well plasmid for STAT3 using Lipofectamine 2000 in Opti-MEM I medium according to the manufacturer's protocols. After 6 h, transfection media was replaced by full DMEM. The next day cells were incubated overnight with starvation media and stimulated with 200ng/mL Leptin for 10 min. Cells were immediately fixed with 4% Formaldehyde in PBS for 20 min at room temperature, permeabilized with 0.2% Triton X-100 for 30 min at room temperature, blocked for 1 h in 3% BSA at room temperature and incubated overnight at 4°C with Mouse anti-HA tag (6E2) (Cell Signaling, 2367) in 1:100 dilution in 3% BSA. Cells were washed three times with PBS for 5 min, incubated with goat anti mouse secondary antibody Alexa Fluor 488 in 1:200 dilution in 3% BSA for 1 h at room temperature, washed 2 times with PBS for 5 min, incubated with DAPI in 1:500 dilution in PBS for 10 min and kept in PBS. Cells in the 96 well plates were imaged in the Opera Phenix High Content Screening Confocal system, obtaining 9 images per well. Quantification of nuclear and cytoplasmic localization was performed with the Harmony software (Perkin Elmer) using the Alexa 488 signal and nucleus to cytoplasm ratio was calculated by dividing the number of cells/well with positive signal in the nucleus (normalized to total number of cells in the well) to the number of cells/well with positive signal in the cytoplasm (normalized to total number of cells in the well). Slides were imaged using a Leica SP8 confocal microscope (Leica Microsystems). In both experiments images were processed using FIJI.

In Vitro Immunoprecipitation Assay

HEK293 cells stably expressing the leptin receptor were seeded in 10cm cell culture dishes coated with Poly-D-Lysine (500.000 cells/well). Cells were starved overnight, stimulated with 200ng/mL insulin (Sigma, i9278) or leptin for 15 min and lysed with cell lysis buffer containing 50 mM Tris, 50 mM KCL, 10 mM EDTA, 1% NP-40, supplied with protease inhibitor cocktail (Roche cOmplete, Mini Protease Inhibitor Cocktail, 11836153001) and phosphatase inhibitor cocktail A (Roche PhosSTOP, PHOSS-RO). Samples were sonicated 30seconds on/60 s off for 4 times at 4°C using the Diagenode Bioruptor+ (Diagenode) and collected by centrifugation at 14000 rpm for 20 min at 4oC. An aliquot was kept as input and the rest was aliquoted in comparable amounts of protein (1mg/sample) and incubated with 20μl per sample of Protein Beads A (Protein A Sepharose, Abcam, ab193256) for 2 h at 4°C (pre-clean up). Samples were span at 800rpm for 30 s and incubated overnight with 5μl of normal Rabbit IgG (Cell Signaling, 2729) or Rabbit anti-IRS2 (L1326) (Cell Signaling, 3089). The next day Protein Beads A were blocked with 5% BSA and 1% NP40 for 2 h at 4°C, incubated with the sample and antibody mix for 3 h at 4°C and washed 4 times with Tris-buffered saline (TBS) supplemented with 0.1% Tween 20 (TBS-T) at room temperature. Beads were eluted in 20μl PBS, 3μl Bolt reducing agent (Thermo, B0009) and 7μl Bolt LDS sample buffer (Thermo, B0007) for 10 min at room temperature followed by 10 min at 95°C and final centrifugation for 2.5 min at 8000 rpm. For the input 20μl per sample were resuspended in Bolt LDS sample buffer and Bolt reducing agent and heated for 10 min at 95°C. Equal volume of samples were loaded and protein electrophoresis was performed using Bolt 4%–12% Bis-Tris Plus gels (Thermo, NW04125BOX) and transferred onto nitrocellulose membrane using an iBLOT (Thermo, IB301001). After blocking with 5% milk solution in TBS-T for 1 h at room temperature, membranes were probed overnight at 4°C using Rabbit anti-PHIP (Abcam, ab86244) at 1:200 dilution in 5% milk in TBS-T. Cells were washed three times with TBS-T for 10 min at room temperature with gentle shaking and incubated with secondary antibody, Goat anti-rabbit IgG-HRP (Dako, P0448) diluted 1:2000 in 5% milk in TBS-T for 1 h at room temperature. Bands were developed using enhanced chemiluminescence (ECL) substrate (Promega, W1015) and images were captured with an ImageQuant LAS 4000 (GE Healthcare). The band intensity of western blots was quantified using FIJI.

Western Blotting

HEK293 cells stably expressing the leptin receptor were seeded in 6 well plates coated with Poly-D-Lysine (50.000 cells/well). Cells were starved overnight, stimulated with 200ng/mL leptin for indicated periods of time and lysed in radio-immunoprecipitation assay

buffer (RIPA) (Sigma, R0278) supplemented with protease and phosphatase inhibitors. Cells were harvested by centrifugation at 14.000 rpm for 30 min and prepared for electrophoresis as described previously. Membranes were blocked with 5% BSA solution in TBS-T for 1 h at room temperature and probed overnight at 4°C using Rabbit anti-p44/42 MAPK (Erk1/2) (137F5) at 1:1000 dilution (Cell Signaling Technology, 4695), Rabbit anti-Phospho-p44/42 MAPK (Erk1/2) (Thr202/Tyr204) at 1:1000 dilution (Cell Signaling Technology, 9101), Rabbit anti-STAT3 at 1:1000 dilution (Cell Signaling Technology, 4904), Rabbit anti-Phospho STAT3 (pY705) (Cell Signaling Technology, 9131) at 1:1000 dilution, Rabbit anti-PHIP at 1:1000 dilution (Proteintech, 20933-1-AP) and Rabbit anti-βActin (Cell Signaling Technology, 4967) at 1:5000 dilution all prepared in the blocking buffer. Cells were washed three times with TBS-T for 10 min at room temperature with gentle shaking and were incubated with secondary antibody, Goat anti-rabbit IgG-HRP (Dako, P0448) diluted 1:2000 in 5% BSA in TBS-T for 1 h at room temperature. Bands were developed as described previously.

QUANTIFICATION AND STATISTICAL ANALYSIS

Sequencing

All genetic data were on build GRCh37 coordinates.

Stage 1 Data

Stage 1 analysis included existing sequence data generated as part of other whole-exome sequencing efforts (details below).

Sequencing and Variant QC

Details of sequencing and variant calling for the SCOOP cases, as part of the UK10K exomes, and INTERVAL controls can be found elsewhere (Hendricks et al., 2018; Walter et al., 2015; Singh et al., 2016). Briefly, single–sample variant calling using GATK Haplotype Caller (v3.2) was performed on the union of Agilent v3 and v5 targets plus a 100 base pair flanking region on 9,795 UK10K and INTERVAL samples, including SCOOP cases (N = 982) and INTERVAL controls (N = 4,499). The called variants were then merged into 200 sample batches and were joint-called using GenotypeVCFs and default settings (DePristo et al., 2011; Van der Auwera et al., 2013). To ensure high-quality variant calls across all datasets and sequencing batches, only variants with at least 7x coverage in at least 80% of samples were called. We applied further variant QC keeping only variants with a calibrated VQSR tranche above 99.75% sensitivity, missingness < 20%, Hardy-Weinberg equilibrium $\chi 2$ p value > 10E-8, mean genotype quality $\geq$ 30, and variants in low-complexity regions as described here (Li, 2014). Further, individual genotypes were set to missing if any of the following were true: GQ < 30, alternate allele read depth (DP1)<2, allelic balance (AB, proportion of reads supporting one of the alleles of the genotype) < 0.2, or AB > 0.8.

Sample QC

We used VerifyBamID (v1.0) (Jun et al., 2012) and a threshold of $\geq$ 3% to identify contaminated samples, principal components calculated from the 1000Genomes Phase I integrated call set (Abecasis et al., 2010) using EIGENSTRAT v4.2 (Price et al., 2006) to identify non-Europeans, and pairwise identity by descent estimates from PLINK v1.07 (Purcell et al., 2007) with a threshold of $\geq$ 0.125 to identify related individuals. We also removed samples with a mean read depth lower than 12. This process resulted in 927 SCOOP cases and 4,057 INTERVAL controls for stage 1 analysis. Among these 927 SCOOP cases, 226 were diagnosed with developmental delay in addition to obesity.

Stage 2 Data

Stage 2 data included targeted sequencing data generated within this study and obtained from additional cases and controls, unrelated to stage 1 cases and controls.

Targeted Sequencing and Variant QC

Targeted sequencing was performed at the Wellcome Sanger Institute (WTSI). DNA samples (300ng), genomic (Fenland) or whole-genome amplified (SCOOP), were pooled in up to 384 uniquely indexed paired-end libraries using the Illumina TruSeq Custom Amplicon Library Preparation Kit according to the manufacturer's instructions, ensuring a mix of SCOOP and Fenland samples in each 384-well pool. Amplicons (in the range 220bp-280bp) were designed using Illumina's tool DesignStudio, to cover exons, UTRs and intron/exon boundaries of the 9 genes of interest, based on UCSC hg19. In total, 146 target regions were covered. Sequencing was performed using 384-plexing and paired-end 300 cycles on Illumina MiSeq v2 to give bi-directional coverage of all amplicons.

Data were aligned to the 1000 Genomes Project Phase 2 GRCh37 human reference genome sequence (hs37d5). The CRAM files produced from these alignments were converted to BAM format, *removing* duplicate flagging (SamTools v1.3 and BioBamBam2 v2.0.65). Variant calling was performed using Genome Analysis Toolkit (GATK) v3.6 HaplotypeCaller to call germline SNPs and indels via local re-assembly of haplotypes, and GenotypeGVCFs to perform joint genotyping on all samples together. Variants were annotated with the NCBI dbSNP database build 149, limiting to target regions ± 100bp.

Genotypes were set to missing if their depth was lower than 15, or if their genotype quality (GQ) was lower than 20 for SNPs and lower than 60 for indels. For heterozygous genotypes, we further considered the allele balance (AB). Heterozygous genotypes with AB outside of [15% - 85%] range were set to missing. Heterozygous genotypes with moderate AB in the ranges of [15% - 35%] or [65%–85%] were required to have a depth greater than 25, otherwise the genotype was set to missing. Indels genotypes with depth greater than 2000 were also set to missing.

Variants were removed as part of the quality control process if: i) they mapped more than 100bp away from of any target region; ii) call rate was lower than 90% among cases or among controls; iii) difference in call rates between cases and controls was greater than 2.5%; iv) they failed any GATK hard filtering (QualByDepth > 2 ; FisherStrand > 60 for SNPs or > 200 for indels ; RMSMappingQuality < 40 for SNPs or < 10 for indels; MappingQualityRankSumTest < −12.5; ReadPosRankSumTest < −8 for SNPs or < −20 for indels; StrandOddsRatio > 3.0 for SNPs or > 10.0 for indels); v) window size for filtering adjacent gaps (GapWin) was lower than 3; vi) within 5bp around a gap to be filtered (SnpGap, for SNP). This process led to 858 variants of good quality in the 9 genes of interest.

Sample QC

Of the 1,816 SCOOP samples sequenced five had a missing rate greater than 15% and one had a mean depth below 12. Those six samples were excluded, leaving 1,810 SCOOP cases for downstream analyses. Following the same process, 10 FENLAND samples were excluded for having a missing rate greater than 15%, and three a mean depth below 12. This left 2,647 FENLAND controls for stage 2 analysis.

Validation Sanger Sequencing

Sanger sequencing was performed to validate singleton and doubleton variants identified in stage 1 and stage 2 analyses from genes with promising results (details below). Briefly, customized PCR primers were designed ± 250 bp surrounding the variant; sequencing was performed using BigDye Terminator v3 kit (Applied BioSystems) and analyzed by capillary electrophoresis on an ABI3730 DNA Analyzer platform (Applied Biosystems), according to the manufacturers' instructions. Familial segregation analysis of variants was performed where family samples were available and where family members consented to genetic studies. In total 42 singleton/doubleton variants (48 genotypes) identified by LOF and STRICT analysis in stage 1 analysis were validated by Sanger sequencing in the original cases and controls. All variants confirmed and all 9 genes were taken for stage 2 targeted sequencing. As targeted sequencing was done on genome-amplified case DNA, 19 variants from cases from stage 2 analysis in four genes (*DGKI*, *PHIP*, *ZMYM4* and *ZNF32*) with promising stage 1+2 LOF and STRICT results (Table S6) were taken for sequence validation, of which 13 confirmed. Stage 2 variants seen in controls in the same four genes were also taken for validation: 9 variants were confirmed and 1 doubleton variant was not (Table S6B). Final meta-analysis results of stage 1+ stage 2 validated variants are shown in Table 1.

Adult Obesity Cases and 1958BC Data

Although data were available exome-wide, we only performed lookup of data for four genes that had survived our combined stage1+2 childhood obesity analysis.

Joint Calling, Sequencing and Variant QC

BAM files from the 1958 Birth Cohort and the Adult obesity cases were converted into gVCF format files using HaplotypeCaller version 3.2-2-gec30cee of the Genome Analysis Toolkit (GATK) from the Broad Institute. The regions processed using HaplotyeCaller were restricted to the intersection of the Aigilent V3 and Illumina TruSeq baits, plus 100bp padding at both 3′ and 5′ ends of the baits. The resulting gVCF files were combined in batches of 200 into multi-sample gVCF files using the GATK tool CombineGVCFs. These multi-sample gVCFs were in turn further combined and variants called using the GATK tool GenotypeGVCFs so as to produce a single VCF file containing the genotypes of all the samples included in the study. Quality scores for SNPs described in the VCF file were improved by running GATK VariantRecalibrator, and subsequently applying these scores to the VCF file using GATK ApplyRecalibration.

Variants were removed as part of the quality control process if: i) call rate was lower than 90% among Generation Scotland cases or among TwinsUK cases or among controls; ii) difference in call rates in at least one of the three comparisons was greater than 2.5% (Generation Scotland cases versus TwinsUK cases or Generation Scotland cases versus controls or TwinsUK cases versus controls); iii) they failed any GATK hard filtering available (QualByDepth > 2 ; FisherStrand > 60 for SNPs or > 200 for indels ; RMSMapping-Quality < 40 for SNPs or < 10 for indels; MappingQualityRankSumTest < −12.5; ReadPosRankSumTest < −8 for SNPs or < −20 for indels); iv) window size for filtering adjacent gaps (GapWin) was lower than 3; v) within 5bp around a gap to be filtered (SnpGap, for SNP). This process led to 1,128,931 exome-wide variants of good quality.

Sample QC

Sample quality control was done across good quality variants. We identified one control sample with a missing rate greater than 5%; 13 control samples showing outlier relatedness values with most of the cases and controls, pointing to potential contamination; and two control samples being non-European, based on the principal component analysis. No cases were further excluded. This left 431 adult obesity cases and 984 control samples for analysis.

Variant Annotation

Variant frequency was annotated with respect to the UK10K-cohort reference panel and each of the four global populations in the 1000 Genomes Phase 1 reference panel: African population (YRI, LWK, ASW), American population (MXL, CLM, PUR), Asian population (CHB, CHS, JPT) and European population (CEU, TSI, FIN, GBR, IBS). Variants were annotated for functional consequences and damaging scores using the Ensembl Variant Effect Predictor (VEP) version 79 with the dbNSFP plug-in (dbNSFPv2.9, Feb 3, 2015) (McLaren et al., 2016; Liu et al., 2011, 2013). Allelic changes were defined as loss-of-function (LOF analysis, see below) if the VEP consequence in protein-coding transcripts was among splice_donor_variant, splice_acceptor_variant, stop_gained, frameshift_variant. For allelic changes annotated as missense (VEP consequence in protein-coding transcripts), we further considered five

tools available in dbNSFP to classify the change as damaging: SIFT prediction "damaging," PolyPhen2 HDIV predictions "probably damaging" and "possibly damaging," PolyPhen2 HVAR predictions "probably damaging" and "possibly damaging," LRT prediction "deleterious," and MutationTaster prediction "disease causing automatic" or "disease causing." Allelic changes were defined as strictly damaging (STRICT analysis, see below) if the VEP consequence in protein-coding transcripts was LOF as defined above or stop_lost or initiator_codon_variant or missense classified as damaging by all 5 prediction tools: this category aims to identify allelic changes that are likely to be damaging. Allelic changes were defined as broadly damaging (BROAD analysis, see below) if it was strictly damaging or missense classified as damaging by at least one of the 5 prediction tools: this category aims to discard allelic changes that are likely to be benign.

Single-variant Association Analyses

Stage 1 and Stage 2 Analyses

Case-control association analysis was performed using SNPtest v2.5 with the -newml option, which implements a likelihood ratio test. Analyses were done unadjusted for age and sex because of insufficient power for stratified analysis and, since all obese participants were prepubertal with age < 10yr at recruitment, the study was not designed to address the effect of age or sex. In stage 1, single-variant analysis was performed on all variants regardless of minor allele count (MAC) or imputation quality. Forty-seven variants with p value $< 10^{-4}$ and with case MAF > control MAF (Table S1), as well as an additional 14 variants driving gene-based analyses (based on 10 genes from BROAD analysis) were prioritized for stage 2 follow-up (Figure S1; Table S2C). After LD pruning using PLINK (parameters:–clump-p1 0.0001–clump-p2 0.2–clump-r2 0.5–clump-kb 500), 53 variants remained. Of these, five variants failed assay design resulting in 48 variants that were assayed on the stage 2 samples. Four SNPs were removed due to having a call rate below 80% in either SCOOP cases or Fenland controls resulting in 44 SNPs for analysis in stage 2.

Stage1+2 Meta-Analysis

Fixed-effects meta-analysis, combining the original and replication samples, was performed using the R package Meta and function *metagen*. No single variant passed single-variant genome-wide significance threshold (p value $< 5\times10^{-8}$) after meta-analysis (Table S5).

Gene-based Association Analyses

Stage 1, Stage 2 and Adult Obesity versus Controls

Gene-based association analyses were performed using a nested approach considering three gene-burden tests filtering for variants with different MAF and with different *in silico* variant function predictions. Analyses were done unadjusted for age and sex because of insufficient power for stratified analysis and, since all obese participants were prepubertal with age < 10yr at recruitment, the study was not designed to address the effect of age or sex. Manhattan and QQ plots for the three gene-burden tests are shown in Figure S2. The same approach was used in stage 1 (SCOOP versus INTERVAL, Table S2), stage 2 (SCOOP versus FENLAND, Table S6), and adult obesity (Generation Scotland & TwinsUK versus 1958BC, Table S4).

Rare alleles were defined as alleles having a frequency lower than 1% in each of the four 1000G populations and in the UK10K-Cohort reference panel, and also in at least one of the analysis groups. Namely, for stage 1 analysis, we required the alleles to be rare (< 1%) across 4,057 INTERVAL control samples, or rare across the 927 SCOOP samples, or rare across the 431 adult obesity cases. For stage 2, we required the allele to be rare (< 1%) across 2,647 FENLAND controls, or rare across 1,810 SCOOP samples. For adult obesity versus 1958BC, we required the allele to be rare (< 1%) across 984 1958BC controls, or rare across 431 adult obesity samples.

Similar to rare allele definition, very rare alleles were defined as alleles having a frequency lower than 0.025% in each of the four 1000G populations and in the UK10K-Cohort reference panel, and also in at least one of the analysis group. The MAF < 0.025% was chosen to focus on variants likely to be private to any given family or clan, and avoid definitions based on "not seen in public databases" which change over time.

We performed three nested gene-burden tests. 1- The LOF analysis considered variants with very rare alleles (MAF < 0.025%) for which the change was categorized as being LOF as described above: 6,160 genes with at least 2 variants were analyzed. 2- The STRICT analysis considered variants with very rare alleles (MAF < 0.025%) for which the variants were classified as having a deleterious effect by five *in silico* prediction programmes as described above: 13,496 genes with at least 2 variants were analyzed. 3- The BROAD analysis considered variants with rare alleles (MAF < 1%) for which the change was categorized as broadly damaging as described above: 17,885 genes with at least 2 variants were analyzed.

Gene-based analyses were performed using the *SKATBinary* function from the R package SKAT (version 1.1. 4, April 1, 2016) (Lee et al., 2012). The p values were computed without adjustment (method.bin = "UA"). LOF and STRICT analyses were performed using the burden test implemented in *SKATBinary* (method = "Burden"), while the BROAD analysis (rare and broadly damaging allelic changes) was performed using SKAT-O (method = "SKATO"). All other *SKATBinary* options were set to their default value. Stage 1 yielded seven genes based on LOF and STRICT analysis with OR > 1 and p value $< 10^{-4}$ (Table S2), which were selected for stage 2 targeted sequencing. In addition, BROAD analysis yielded 10 genes with OR > 1 and p value $< 10^{-4}$ (Table S2), of 10 genes, 8 genes were driven by 12 variants based on leave-one-out analysis (see below) and 2 genes were influenced by 2 variants, but signal remained after leave-one-out analysis so were also taken for stage 2 targeted sequencing (Table S2; Figures S1 and 1).

Leave-one-out Analyses

To identify gene-based results driven by one or more variants, we applied the following leave-one-out strategy: 1- among the variants seen more than twice in our stage 1 sample (cases and controls together), we identified the variant with the lowest single-variant analysis p value whenever it is nominally significant ($p < 0.05$); 2- we removed this variant and performed the stage 1 gene-based test again. We repeated steps 1 and 2 until the stage 1 gene-based p value was above 0.1 or there were no additional variants seen more than twice and with a single-variant analysis p value < 0.05. For genes that were driven by one or two single variants (8 genes, Table S2; Figure S1), we genotyped single variants. Otherwise, we sequenced the coding region of the gene.

Stage1+2 Meta-Analyses

Gene-based meta-analysis was performed for 9 genes selected from stage 1 analysis and taken forward for targeted sequencing in stage 2. This analysis was performed using the *MetaSKAT_wZ* function from the R package MetaSKAT (version 0.60, August 17, 2015) (Lee et al., 2013). For the LOF and STRICT analyses, we use the option r.corr = 1 to run a burden test, while for broad analysis, we used the option method = "optimal" to run SKAT-O. All other *MetaSKAT_wZ* options were set to their default value.

Association Analysis Using External Controls

We used ProxECAT (Hendricks et al., 2018), to perform case-control analysis of the burden of LOF and STRICT very rare variants in the gene region for 927 SCOOP cases versus 21,384 external common controls of non-Finnish European descent from gnomAD. ProxECAT tests for a difference in the ratio of very rare functional variants (LOF or STRICT) to very rare synonymous variants between cases and controls. Comparing the ratio of functional to synonymous variants enables the inclusion of external controls, but can reduce power to detect an association. As this analysis uses the same set of cases from the stage 1 analysis, it is not a true replication, but can provide more evidence for or against an association given the different control sample. This can be especially helpful for genes where there were no rare minor alleles identified in the controls from the stage 1 analysis (i.e., STRICT: *ZNF32*, *HEPACAM*; LOF: *ZMYM4, PHIP, VIL1*) (Table S3).

Genotyping

Based on results from the stage 1 single-point analysis (47 variants, see below) and gene-based tests (14 variants, see below) (Figure S1), 53 variants were selected to take forward to stage 2 in an additional 1,810 SCOOP and 3,800 randomly-selected Fenland samples. Of the 53 variants, 48 assays were successfully designed for Agena genotyping (Agena Bioscience) across 2 plexes. Four SNPs failed QC resulting in 44 SNPs for single variant analysis. Sixty-two SCOOP cases and 23 Fenland controls with a call rate below 0.9 were removed, resulting in 1,754 SCOOP cases and 3,777 Fenland controls for single-variant analysis in the stage 2 dataset.

Gene Set Enrichment

A summary of gene sets used is in Table S8.

Gene Sets

Obesity and Syndromic Obesity Gene Set

Genes known to harbor causal, highly penetrant mutations involved in human obesity were taken from Table 1 in Pigeyre et al. (2016) (Tables S8A and S8B).

Developmental Disorder Gene2Phenotype Gene Set

The Developmental Disorder Gene2Phenotype (DDG2P) online system curates genes related to developmental delay and the strength of evidence for the association between the gene and developmental delay. More details can be found here: https://www.ebi.ac.uk/gene2phenotype. DDG2P gene annotation from July 23, 2017 was used for analysis (Tables S8B and S8C).

Constrained and Unconstrained Gene Set

All constraint metrics were from ExAC release 0.3.1 (Samocha et al., 2014) file: *fordist_cleaned_nonpsych_z_pli_rec_null_data.txt*. Two gene sets were created a constrained gene set where pLI > 09 (Tables S8B and S8D) and an unconstrained gene set with genes with pLI $\leq$ 0.9.

Curated List of Known Obesity or BMI Association Genes

The NHGRI-EBI GWAS catalog (https://www.ebi.ac.uk/gwas/, accessed on 25th July 2017) was used to extract a list of obesity/BMI signals, reaching genome-wide significance (p value < $5x10^{-8}$) in Europeans using the search terms "Body Mass Index," "Childhood body mass index," "Obesity," "Obesity (extreme)" and "Obesity (early onset extreme)." Using the reported gene for each signal, we identified 157 unique genes (Tables S8B and S8E).

We performed gene set enrichment analysis similar to previous analyses (Purcell et al., 2014; Singh et al., 2016). Gene set analysis was performed on five primary groupings, each of which had subsets, resulting in 10 primary gene sets for analysis (Table S9B). We repeated primary analysis in patients with obesity and developmental delay (Table S9C) and with obesity alone (Table S9D). A secondary analysis was performed to assess genes that were both LOF constrained (i.e., pLI > 0.9) and GWAS (Table S9E).

Briefly, using PLINK/SEQ (https://atgu.mgh.harvard.edu/plinkseq/index.shtml) we calculated gene region test-statistics for an enrichment of genetic variants in cases compared to controls. For each gene, we evaluated the three analysis groupings used in the gene-based tests: BROAD (MAF < 1% and broadly damaging), STRICT (MAF < 0.025% and strictly damaging), and LOF (MAF < 0.025% and LoF). We then used the SMP utility to calculate the gene set enrichment while controlling for exome-wide differences between cases and controls. Twenty thousand case control permutations were used to estimate the empirical gene set

enrichment p value. We report both the nominal p value and the adjusted p value, which adjusts for all ten primary gene sets investigated within each analysis grouping (i.e., BROAD, STRICT, and LOF). A Chi-square test of Independence was used to compare the overlap of genes in the GWAS gene set that were also LOF constrained.

Functional Work Statistical Analysis

Distributions of average nuclei/cytoplasm ratio were compared between *PHIP* functionally studied variants seen only in cases and variants seen in controls by a Wilcoxon rank sum test.

Mouse Statistical Analysis

For all analyses, the individual mouse was considered the experimental unit within the studies. All mutant data (7 females and 7 males) from the pipeline was collected across multiple batches and were compared to a year's worth of overlapping control data collected on mice from the same genetic background (up to 226 females and 231 males) for DEXA, ipGTT and CC (total 43 parameters). As a high-throughput project, the target sample size of 14 mutant animals per strain is relatively low. This was determined after a community-wide debate to find the lowest sample size that would balance resource usage while detecting phenotypic abnormalities. For analysis of continuous data, the current work uses linear mixed models to allow modeling of multiple sources of variability on a phenotype. The approach was previously discussed by Karp and colleagues (Karp et al., 2012) as a way of integrating factors like genotype (G), sex (S) and genotype*sex (G*S) when performing phenotypic analysis of the mice. These factors are assumed to have a fixed values. Given our multi-batch experimental design strategy, linear mixed models also allow us to include batch effects as a random effect, with the assumption that animals from the same batch will have correlated phenotypes. This arises from factors such as technician skill, reagent lot, cage environment, maternal ability and genotype, and litter size. In the study of Karp and colleagues (Karp et al., 2012) two equations were put forward: Equation 1 (below) includes G, S and G*S and batch effect as described. Equation 2 is similar to Equation 1 but includes body weight as an additional factor affecting phenotype.

$$Y \sim Genotype + Sex + Genotype*Sex + (1|Batch) \quad \text{(Equation 1)}$$

We used Equation 1 here since body weight is one of the phenotypes that we are interested in measuring and since body weight does not scale linearly (or at all) with many of the parameters that we are testing in our global phenotypic screen. Analysis were performed using PhenStat (Kurbatova et al., 2015), an R package version 2.18.0 available from Bioconductor (Gentleman et al., 2004). The package's mixed model framework was used as default except the argument equationType was set to withoutWeight and dataPointsThreshold was set to 2. The genotype contribution test p value was adjusted for multiple testing to control the false discovery rate to 5%. This statistical method has been studied through simulations and resampling studies (Karp et al., 2014) and found to be robust and reliable with a multi-batch workflow, where the knockout mice are split into multiple phenotyping batches. All statistics are shown in the corresponding figures (Figures S6 and S7) and corresponding figure legends.

Expression Data

Expression data and corresponding plots (Figure S3) were obtained from https://gtexportal.org/home/.

Cell Metabolism, Volume *31*

Supplemental Information

Exome Sequencing Identifies Genes and Gene Sets Contributing to Severe Childhood Obesity, Linking *PHIP* Variants to Repressed *POMC* Transcription

Gaëlle Marenne, Audrey E. Hendricks, Aliki Perdikari, Rebecca Bounds, Felicity Payne, Julia M. Keogh, Christopher J. Lelliott, Elana Henning, Saad Pathan, Sofie Ashford, Elena G. Bochukova, Vanisha Mistry, Allan Daly, Caroline Hayward, INTERVAL, UK10K Consortium, Nicholas J. Wareham, Stephen O'Rahilly, Claudia Langenberg, Eleanor Wheeler, Eleftheria Zeggini, I. Sadaf Farooqi, and Inês Barroso

Supplementary Figures and Tables

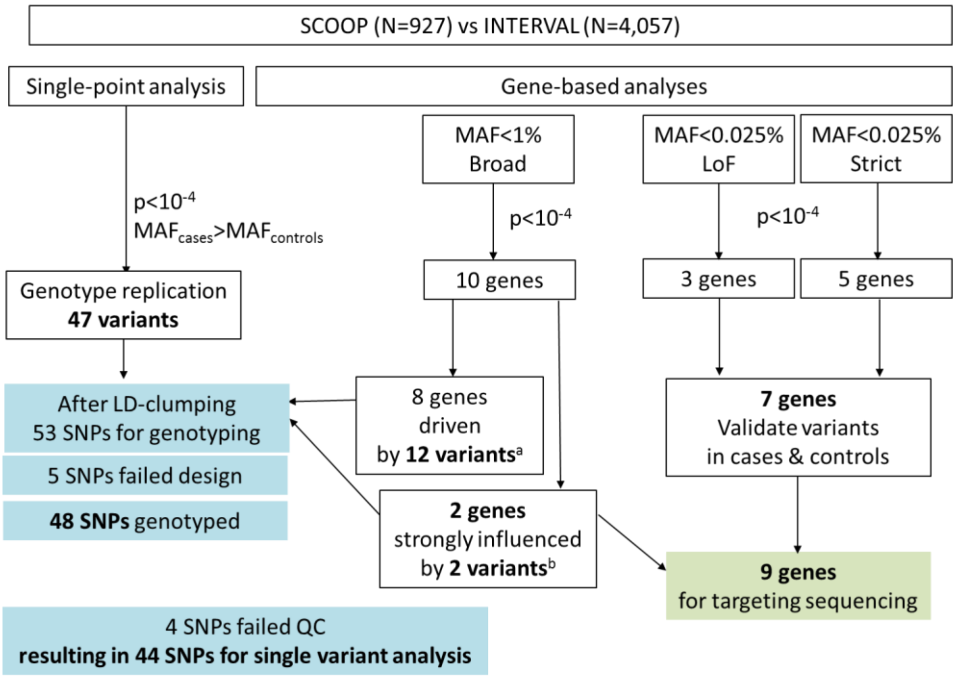

Supplementary Figure 1, Related to Figure 1 – Flow Diagram Single-point and Gene-based Analyses. Flow diagram showing the different analysis and main association results based on stage 1 analysis with 927 SCOOP cases and 4,057 INTERVAL participants as controls.

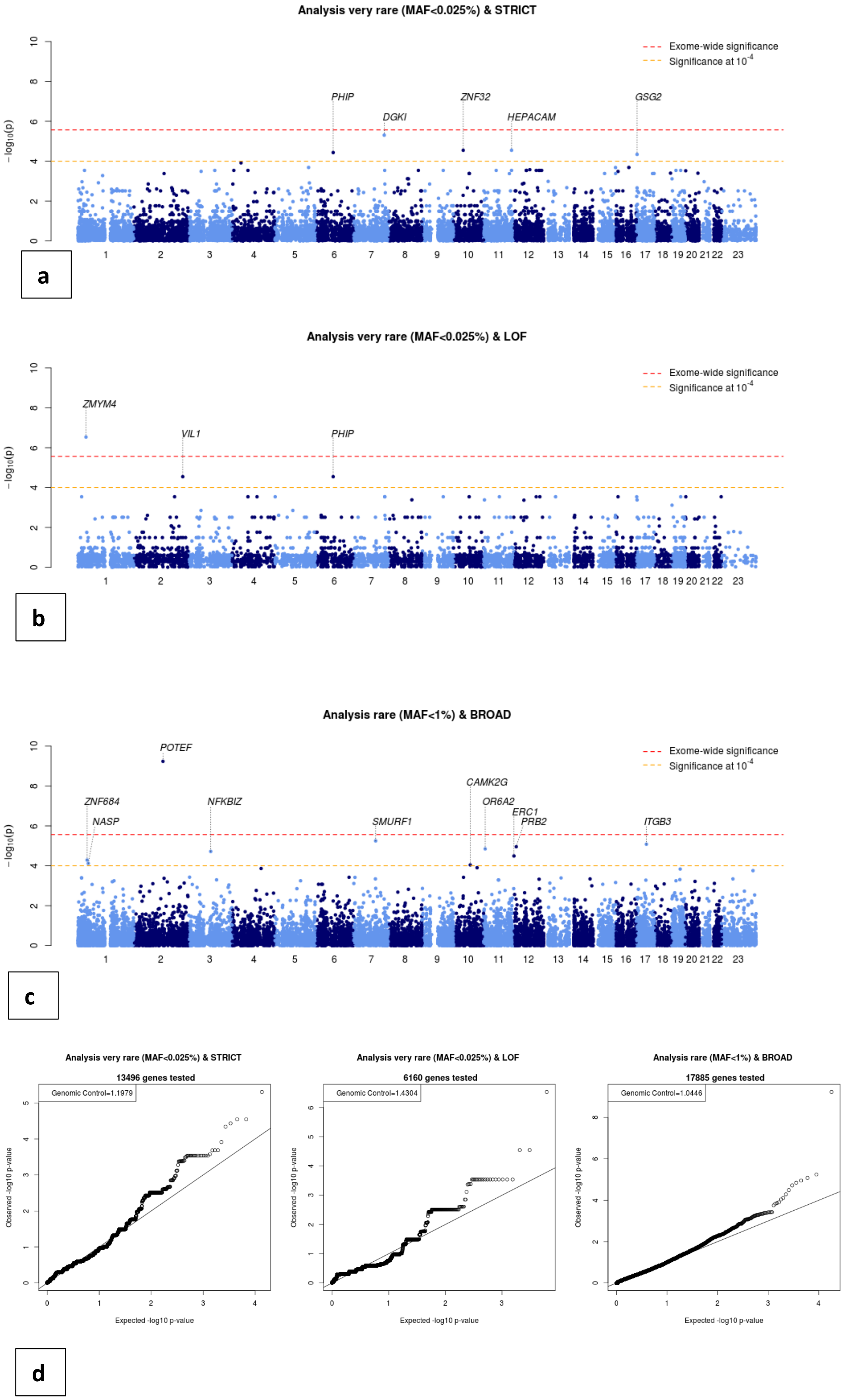

Analysis very rare (MAF<0.025%) & STRICT
Exome-wide significance
Significance at 10^-4
PHIP
DGKI
ZNF32
HEPACAM
GSG2
−log10(p)
a
Analysis very rare (MAF<0.025%) & LOF
ZMYM4
VIL1
PHIP
b
Analysis rare (MAF<1%) & BROAD
POTEF
ZNF684
NASP
NFKBIZ
SMURF1
CAMK2G
OR6A2
ERC1
PRB2
ITGB3
c
13496 genes tested
Genomic Control=1.1979
6160 genes tested
Genomic Control=1.4304
17885 genes tested
Genomic Control=1.0446
Observed -log10 p-value
Expected -log10 p-value
d

Supplementary Figure 2, Related to STAR Methods – Manhattan and QQ plots of gene-based association results. Manhattan plot STRICT analysis (a), Manhattan plot LOF analysis (b) Manhattan plot BROAD analysis (c). Genes taken forward for validation in stage 2 analysis are annotated. QQ plots of association results for gene-based analysis (d): left (STRICT analysis), middle (LOF analysis), right (BROAD analysis).

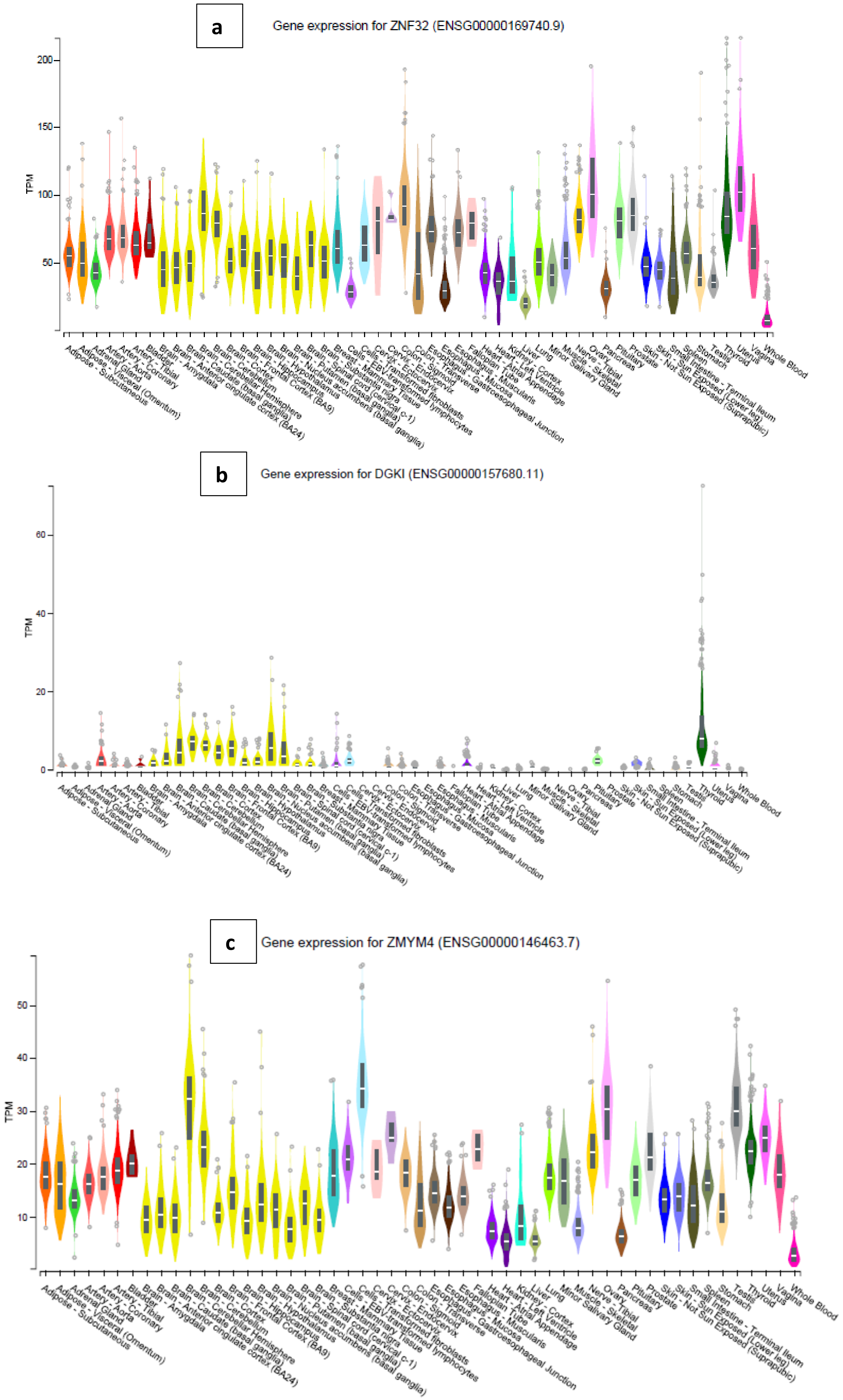
a
Gene expression for ZNF32 (ENSG00000169740.9)
TPM
200
150
100
50
Adipose - Subcutaneous
Adipose - Visceral (Omentum)
Adrenal Gland
Artery - Aorta
Artery - Coronary
Artery - Tibial
Bladder
Brain - Amygdala
Brain - Anterior cingulate cortex (BA24)
Brain - Caudate (basal ganglia)
Brain - Cerebellar Hemisphere
Brain - Cerebellum
Brain - Cortex
Brain - Frontal Cortex (BA9)
Brain - Hippocampus
Brain - Hypothalamus
Brain - Nucleus accumbens (basal ganglia)
Brain - Putamen (basal ganglia)
Brain - Spinal cord (cervical c-1)
Brain - Substantia nigra
Breast - Mammary Tissue
Cells - EBV-transformed lymphocytes
Cells - Transformed fibroblasts
Cervix - Ectocervix
Cervix - Endocervix
Colon - Sigmoid
Colon - Transverse
Esophagus - Gastroesophageal Junction
Esophagus - Mucosa
Esophagus - Muscularis
Fallopian Tube
Heart - Atrial Appendage
Heart - Left Ventricle
Kidney - Cortex
Liver
Lung
Minor Salivary Gland
Muscle - Skeletal
Nerve - Tibial
Ovary
Pancreas
Pituitary
Prostate
Skin - Not Sun Exposed (Suprapubic)
Skin - Sun Exposed (Lower leg)
Small Intestine - Terminal Ileum
Spleen
Stomach
Testis
Thyroid
Uterus
Vagina
Whole Blood
b
Gene expression for DGKI (ENSG00000157680.11)
TPM
60
40
20
0
Adipose - Subcutaneous
Adipose - Visceral (Omentum)
Adrenal Gland
Artery - Aorta
Artery - Coronary
Artery - Tibial
Bladder
Brain - Amygdala
Brain - Anterior cingulate cortex (BA24)
Brain - Caudate (basal ganglia)
Brain - Cerebellar Hemisphere
Brain - Cerebellum
Brain - Cortex
Brain - Frontal Cortex (BA9)
Brain - Hippocampus
Brain - Hypothalamus
Brain - Nucleus accumbens (basal ganglia)
Brain - Putamen (basal ganglia)
Brain - Spinal cord (cervical c-1)
Brain - Substantia nigra
Breast - Mammary Tissue
Cells - EBV-transformed lymphocytes
Cells - Transformed fibroblasts
Cervix - Ectocervix
Cervix - Endocervix
Colon - Sigmoid
Colon - Transverse
Esophagus - Gastroesophageal Junction
Esophagus - Mucosa
Esophagus - Muscularis
Fallopian Tube
Heart - Atrial Appendage
Heart - Left Ventricle
Kidney - Cortex
Liver
Lung
Minor Salivary Gland
Muscle - Skeletal
Nerve - Tibial
Ovary
Pancreas
Pituitary
Prostate
Skin - Not Sun Exposed (Suprapubic)
Skin - Sun Exposed (Lower leg)
Small Intestine - Terminal Ileum
Spleen
Stomach
Testis
Thyroid
Uterus
Vagina
Whole Blood
c
Gene expression for ZMYM4 (ENSG00000146463.7)
TPM
50
40
30
20
10
Adipose - Subcutaneous
Adipose - Visceral (Omentum)
Adrenal Gland
Artery - Aorta
Artery - Coronary
Artery - Tibial
Bladder
Brain - Amygdala
Brain - Anterior cingulate cortex (BA24)
Brain - Caudate (basal ganglia)
Brain - Cerebellar Hemisphere
Brain - Cerebellum
Brain - Cortex
Brain - Frontal Cortex (BA9)
Brain - Hippocampus
Brain - Hypothalamus
Brain - Nucleus accumbens (basal ganglia)
Brain - Putamen (basal ganglia)
Brain - Spinal cord (cervical c-1)
Brain - Substantia nigra
Breast - Mammary Tissue
Cells - EBV-transformed lymphocytes
Cells - Transformed fibroblasts
Cervix - Ectocervix
Cervix - Endocervix
Colon - Sigmoid
Colon - Transverse
Esophagus - Gastroesophageal Junction
Esophagus - Mucosa
Esophagus - Muscularis
Fallopian Tube
Heart - Atrial Appendage
Heart - Left Ventricle
Kidney - Cortex
Liver
Lung
Minor Salivary Gland
Muscle - Skeletal
Nerve - Tibial
Ovary
Pancreas
Pituitary
Prostate
Skin - Not Sun Exposed (Suprapubic)
Skin - Sun Exposed (Lower leg)
Small Intestine - Terminal Ileum
Spleen
Stomach
Testis
Thyroid
Uterus
Vagina
Whole Blood

Supplementary Figure 3, Related to STAR Methods – GTEX expression profiles of select genes.
ZNF32 (a), *DGKI* (b), *ZMYM4* (c).

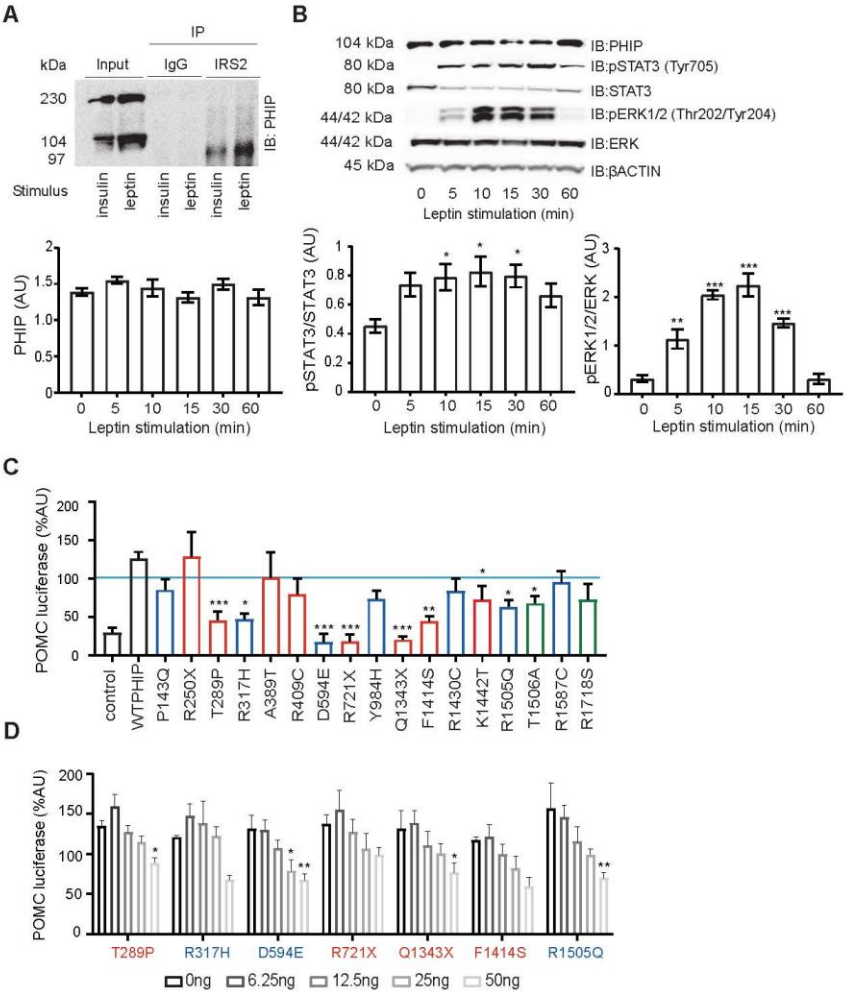

Supplementary Figure 4, Related to Figure 3 - Functional characterisation of WT and mutant forms of PHIP. A) Representative western blot depicting the interaction between endogenous PHIP and IRS2 upon insulin and leptin stimulation. The 230kDa and 104kDa endogenous PHIP isoforms were used as input as shown. B) Representative western blot of endogenous PHIP, pSTAT3 and pERK upon leptin stimulation and quantification of PHIP protein expression and STAT3 and ERK phosphorylation in different time points. n= 4, mean+/-SEM, ordinary one-way ANOVA multiple comparisons to time point 0min, *p<0.05, **p<0.01, ***p<0.001. C) Effect of human PHIP mutants on POMC transcription compared to WT in leptin stimulated conditions, n=4-6, mean + SEM, ordinary one-way ANOVA multiple comparisons to WT, *p<0.05, **p<0.01, ***p<0.001. Blue line : WT in basal conditions D) Dominant negative effect of human PHIP mutants in POMC transcription in leptin stimulated conditions, n=3, mean + SEM, ordinary one-way ANOVA multiple comparisons to 0ng *p<0.05, **p<0.01, ***p<0.001.

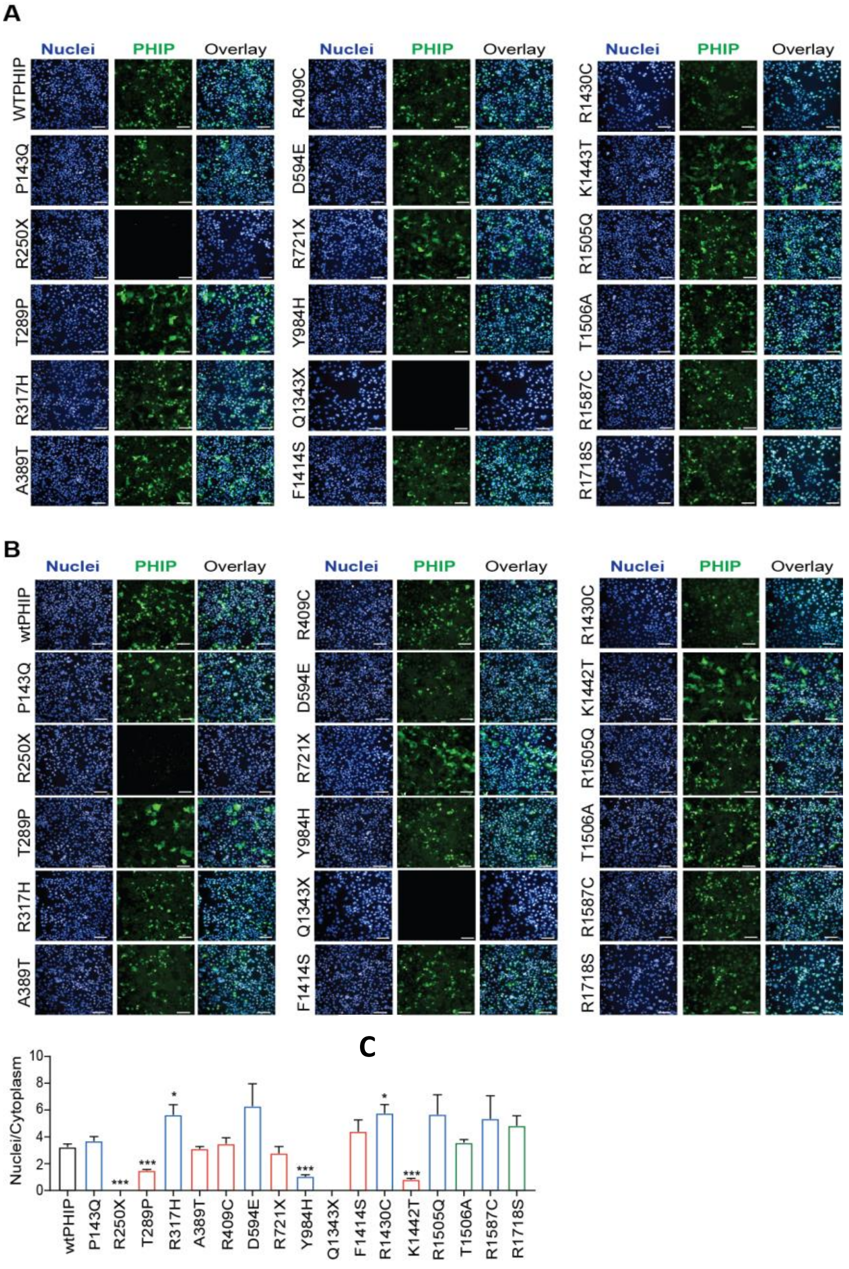
A
Nuclei
PHIP
Overlay
WTPHIP
P143Q
R250X
T289P
R317H
A389T
R409C
D594E
R721X
Y984H
Q1343X
F1414S
R1430C
K1443T
R1505Q
T1506A
R1587C
R1718S
B
Nuclei
PHIP
Overlay
wtPHIP
P143Q
R250X
T289P
R317H
A389T
R409C
D594E
R721X
Y984H
Q1343X
F1414S
R1430C
K1442T
R1505Q
T1506A
R1587C
R1718S
C
Nuclei/Cytoplasm
0
2
4
6
8
10
*
***
*
***
***
***
wtPHIP
P143Q
R250X
T289P
R317H
A389T
R409C
D594E
R721X
Y984H
Q1343X
F1414S
R1430C
K1442T
R1505Q
T1506A
R1587C
R1718S

Supplementary Figure 5, Related to Figure 3 - Cellular localisation of WT and mutant forms of PHIP. A-B) Representative confocal fluorescence microscopy images showing protein localization of PHIP mutants in basal (A) and leptin stimulated (B) conditions and (C) quantification of nuclei to cytoplasm ratio of PHIP subcellular localization for leptin stimulated conditions, n=3, mean + SEM, two-tailed unpaired T-test with Welch's correction, *p<0.05, **p<0.01, ***p<0.001.

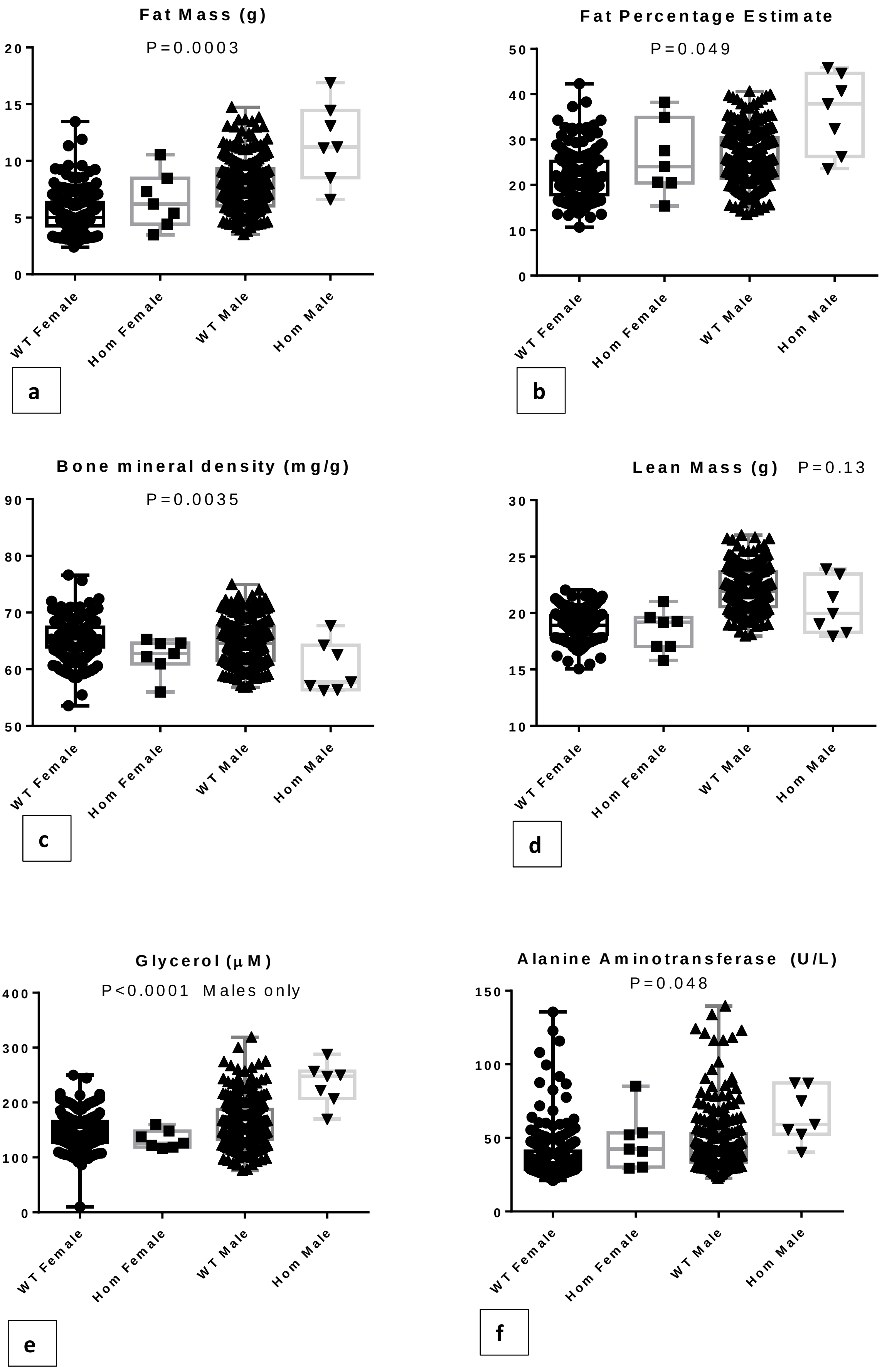
Fat Mass (g)
P=0.0003
0
5
10
15
20
WT Female
Hom Female
WT Male
Hom Male
a
Fat Percentage Estimate
P=0.049
0
10
20
30
40
50
WT Female
Hom Female
WT Male
Hom Male
b
Bone mineral density (mg/g)
P=0.0035
50
60
70
80
90
WT Female
Hom Female
WT Male
Hom Male
c
Lean Mass (g) P=0.13
10
15
20
25
30
WT Female
Hom Female
WT Male
Hom Male
d
Glycerol (μM)
P<0.0001 Males only
0
100
200
300
400
WT Female
Hom Female
WT Male
Hom Male
e
Alanine Aminotransferase (U/L)
P=0.048
0
50
100
150
WT Female
Hom Female
WT Male
Hom Male
f

Supplementary Figure 6, Related to STAR methods– Phenotyping results in wild-type and *Dgki*$^{em1(IMPC)Wtsi}$ homozygous mice. Boxplots showing fat mass (a), fat percentage (b), bone mineral density (c), lean mass (d), glycerol (e) and alanine aminotransferase (f). Wild-type (WT) and *Dgki*$^{em1(IMPC)Wtsi}$ homozygous mice (Hom) results are shown. P-values for genotype effect are shown. Numbers included in (a-d) are Males: WT n=229, Hom n=7; Females: WT n=225, Hom n=7, (e) M: WT n=228, Hom n=7; Females: WT n=223, Hom n=7; (f) Males: WT n=227, Hom n=7; Females: WT n=222, Hom n=7.

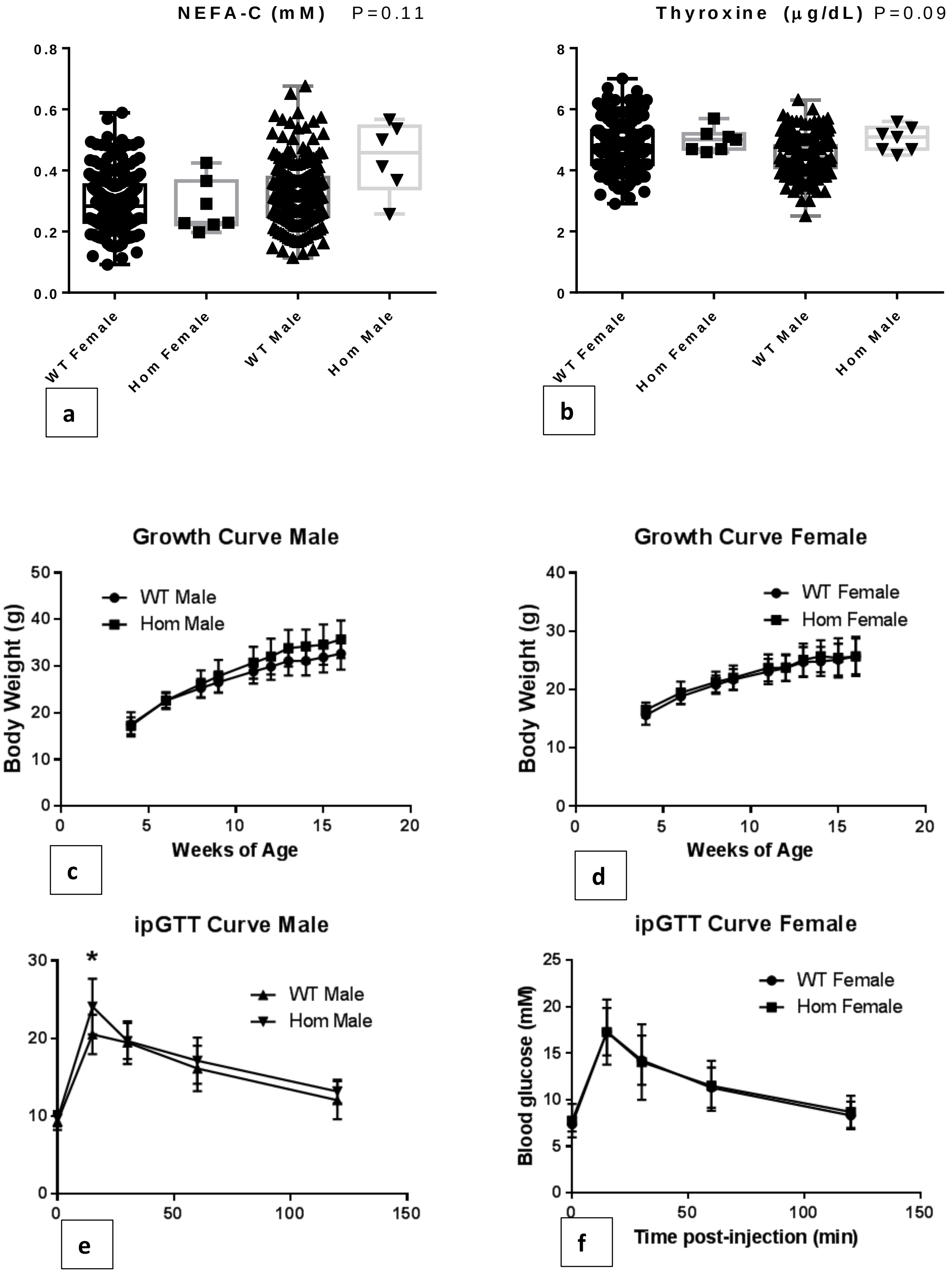

NEFA-C (mM) P=0.11
0.0
0.2
0.4
0.6
0.8
WT Female
Hom Female
WT Male
Hom Male
a
Thyroxine (µg/dL) P=0.09
0
2
4
6
8
WT Female
Hom Female
WT Male
Hom Male
b
Growth Curve Male
Body Weight (g)
WT Male
Hom Male
Weeks of Age
c
Growth Curve Female
Body Weight (g)
WT Female
Hom Female
Weeks of Age
d
ipGTT Curve Male
*
WT Male
Hom Male
e
ipGTT Curve Female
Blood glucose (mM)
WT Female
Hom Female
Time post-injection (min)
f

Supplementary Figure 7, Related to STAR methods– Phenotyping results in wild-type and *Dgki*$^{em1(IMPC)Wtsi}$ homozygous mice. Boxplots showing NEFA-X (a) and thyroxine (b). Growth curves for male (c) and female (d) mice are shown with mean and standard deviation. Results from ipGTT for male (e) and female (f) mice are shown. Wild-type (WT) and *Dgki*$^{em1(IMPC)Wtsi}$ homozygous mice (Hom) results are shown. P-values for genotype effect are shown for (a-b). All genotype effects (combined male and female are non-significant for growth (c-d): week 4 (p=0.530), week 9 (p=0.400), week 13 (p=0.076), week 16 (p=0.101). P-values for genotype effects (combined male and female) for blood glucose concentration (e-f): T0 (p=0.354), T15 (p=0.040), T30 (p=0.913), T60 (p=0.462), T120 (p=0.366). Numbers included are (a) Males: WT n=221, Hom n=7; Females: WT n=220, Hom n=7; (b) Males: WT n=227, Hom n=7; Females: WT n=7, Hom n=222; (c) WT n=230, Hom n=7; (d) WT n=226, Hom n=7; (e) WT n=189, Hom n=6; and (f) WT n=180, Hom n=5.

gene	SCOOP			gnomAD			strict / syn			lof / syn		
	strict	LoF	syn	strict	LoF	syn	scoop	gnomad	p-value	scoop	gnomad	p-value
DGKI	7	1	6	46	7	102	1.167	0.451	1.05E-01	0.167	0.069	4.78E-01
ERC1	2	0	6	88	15	83	0.333	1.06	1.35E-01	0	0.181	1.65E-01
GSG2	5	4	3	37	21	73	1.667	0.507	1.09E-01	1.333	0.288	5.72E-02
HEPACAM	5	0	1	28	1	39	5	0.718	4.41E-02	NA	0.026	NA
NASP	1	0	9	19	4	76	0.111	0.25	4.12E-01	NA	0.053	NA
PHIP	9	4	5	47	5	129	1.8	0.364	4.96E-03	0.8	0.039	4.93E-04
VIL1	6	4	4	89	17	161	1.5	0.553	1.25E-01	1	0.106	4.99E-03
ZMYM4	7	6	4	58	10	123	1.75	0.472	3.77E-02	1.5	0.081	7.10E-05
ZNF32	4	1	2	8	1	33	2	0.242	2.14E-02	0.5	0.03	9.92E-02

ST3, Related to STAR Methods - ProxECAT results for the 9 genes. Column headers are: gene -gene symbol; strict -number of alternate alleles in STRICT category; LoF - number of alternate alleles in LoF category; syn - number of synonymous alternate alleles; strict / syn - ratio of strict to synonymous alternate alleles; lof / syn - ratio of strict to synonymous alternate alleles; p-value - ProxECAT p-value testing for association with the gene region.

Gene	Analysis	p-value	# variants	Allele counts Cases	Allele counts 1958BC	OR [95% CI] (adjusted)
DGKI	very rare (MAF<0.025%) & STRICT	0.8230	2	1	3	0.76 [0.08-7.32]
DGKI	very rare (MAF<0.025%) & LOF	0.1307	1	1	0	Inf (6.85 [0.28-168.44])
PHIP	very rare (MAF<0.025%) & STRICT	0.9138	3	1	2	1.14 [0.1-12.6]
PHIP	very rare (MAF<0.025%) & LOF	-	0	0	0	-
ZMYM4	very rare (MAF<0.025%) & STRICT	0.1725	3	2	1	4.57 [0.41-50.41]
ZMYM4	very rare (MAF<0.025%) & LOF	-	0	0	0	-

ST4, related to STAR Methods - Results from gene-based analyses in UK10K-Adult Obese versus 1958BC controls for 3 selected genes (*PHIP, DGKI* and *ZMYM4*). There were no LOF or STRICT variants with MAF<0.025% for analysis in *ZNF32*. Column headers are: Gene - Gene symbol; Analysis - Analysis performed; p-value - burden test p-value; # variants - Number of variants contributing to the gene-based test; Allele counts Cases - Allele counts in adult obese cases; Allele counts 1958BC - Allele counts in 1958BC controls; OR [95% CI] (adjusted) - Odds ratio [95% confidence interval]. If allele count was 0 in cases or controls, an adjusted OR and its CI was computed by adding 0.5 to each of the 2 by 2 table cell.

Stage 1			
**Analysis**	**BROAD**	**STRICT**	**LOF**
AC INTERVAL (N=4057)	29	6	0
AC SCOOP (N=927)	14	9	4
AC SCOOP with DD (N=226)	7	4	2
AC SCOOP without DD (N=701)	7	5	2
p-value SCOOP v controls	0.0059	3.67E-05	2.84E-05
p-value SCOOP with DD v controls	1.48E-10	8.67E-07	2.06E-09
p-value SCOOP without DD v controls	0.0880	0.0040	0.0007
OR SCOOP v controls	2.11 [1.12-4]	6.57 [2.34-18.45]	Inf(39.41 [2.12-732.02])
OR SCOOP with DD v controls	4.33 [1.9-9.9]	11.98 [3.38-42.45]	Inf(89.83 [4.31-1871.85])
OR SCOOP without DD v controls	1.4 [0.61-3.19]	4.82 [1.47-15.81]	Inf(28.95 [1.39-602.99])
**Stage 2 (post sequence validation)**			
**Analysis**	**BROAD**	**STRICT**	**LOF**
AC FENLAND (N=2647)	9	3	0
AC SCOOP (N=1810)	20	5	3
AC SCOOP with DD (N=411)	4	2	2
AC SCOOP without DD (N=1399)	16	3	1
p-value SCOOP v controls	0.0032	0.2070	0.0361
p-value SCOOP with DD v controls	0.0210	0.0814	0.0003
p-value SCOOP without DD v controls	0.0021	0.4267	0.1689
OR SCOOP v controls	3.28 [1.49-7.2]	2.45 [0.59-10.27]	Inf(10.26 [0.53-198.57])
OR SCOOP with DD v controls	2.9 [0.89-9.42]	4.34 [0.72-25.95]	Inf(32.28 [1.55-672.63])
OR SCOOP without DD v controls	3.39 [1.5-7.68]	1.9 [0.38-9.43]	Inf(5.69 [0.23-139.57])
**META-ANALYSIS (post sequence validation)**			
**Analysis**	**BROAD**	**STRICT**	**LOF**
AC controls (N=6704)	38	9	0
AC SCOOP (N=2737)	34	14	7
AC SCOOP with DD (N=637)	11	6	4
AC SCOOP without DD (N=2100)	23	8	3
p-value SCOOP v controls	0.0002	9.81E-05	1.23E-05
p-value SCOOP with DD v controls	3.75E-07	3.75E-06	3.19E-10
p-value SCOOP without DD v controls	0.0030	0.0091	0.0006
OR SCOOP v controls	2.49 [1.57-3.96]	4.35 [1.88-10.06]	Inf(39.74 [2.27-695.83])
OR SCOOP with DD v controls	3.81 [1.95-7.46]	8.54 [3.04-23.99]	Inf(95.01 [5.11-1765.21])
OR SCOOP without DD v controls	2.19 [1.3-3.68]	3.18 [1.23-8.25]	Inf(26.95 [1.39-521.79])

ST7, Related to Figure 2 - Results from *PHIP* gene-based analyses stratifying cases according the presence or absence of developmental delay. Column headers: AC -allele count, i.e. number of the rare alleles; N - Total number of alleles; DD - developmental delay,refers to analyses limitesd to obesity cases who also have developmental delay, or to those that are obese without developmental delay (without DD); OR - odds ratio and [95% CI]. If allele count was 0 in cases or controls, an adjusted OR and its CI was computed by adding 0.5 to each of the 2 by 2 table cell; Stage1, Stage 2- refer to analyses performed in Stage 1 and Stage 2 participants with either BROAD, STRICT or LOF tests.
